# Differential modulation of mutant CALR and JAK2 V617F-driven oncogenesis by HLA genotype in myeloproliferative neoplasms

**DOI:** 10.3389/fimmu.2024.1427810

**Published:** 2024-09-16

**Authors:** Velizar Shivarov, Gergana Tsvetkova, Ilina Micheva, Evgueniy Hadjiev, Jasmina Petrova, Anela Ivanova, Galia Madjarova, Milena Ivanova

**Affiliations:** ^1^ Department of Experimental Research, Medical University Pleven, Pleven, Bulgaria; ^2^ Department of Clinical Hematology, Alexandrovska University Hospital, Medical University Sofia, Sofia, Bulgaria; ^3^ Department of Clinical Hematology, Saint Marina University Hospital, Medical University Varna, Varna, Bulgaria; ^4^ Department of Physical Chemistry, Faculty of Chemistry and Pharmacy, Sofia University “St. Kl. Ohridski”, Sofia, Bulgaria; ^5^ Department of Clinical Immunology, Alexandrovska University Hospital, Medical University Sofia, Sofia, Bulgaria

**Keywords:** immunoediting, HLA, MPN, mutation, CALR, JAK2 V617F, neoantigen

## Abstract

It has been demonstrated previously that human leukocyte antigen class I (*HLA-I*) and class II (*HLA-II*) alleles may modulate JAK2 V617F and CALR mutation (CALRmut)-associated oncogenesis in myeloproliferative neoplasms (MPNs). However, the role of immunogenetic factors in MPNs remains underexplored. We aimed to investigate the potential involvement of *HLA* genes in CALRmut+ MPNs. High-resolution genotyping of *HLA-I* and *-II* loci was conducted in 42 CALRmut+ and 158 JAK2 V617F+ MPN patients and 1,083 healthy controls. A global analysis of the diversity of HLA-I genotypes revealed no significant differences between CALRmut+ patients and controls. However, one *HLA-I* allele (C*06:02) showed an inverse correlation with presence of CALR mutation. A meta-analysis across independent cohorts and healthy individuals from the 1000 Genomes Project confirmed an inverse correlation between the presentation capabilities of the *HLA-I* loci for JAK2 V617F and CALRmut-derived peptides in both patients and healthy individuals. scRNA-Seq analysis revealed low expression of *TAP1* and *CIITA* genes in CALRmut+ hematopoietic stem and progenitor cells. In conclusion, the *HLA-I* genotype differentially restricts JAK2 V617F and CALRmut-driven oncogenesis potentially explaining the mutual exclusivity of the two mutations and differences in their presentation latency. These findings have practical implications for the development of neoantigen-based vaccines in MPNs.

## 1 Introduction

Our understanding of the biology of myeloproliferative neoplasms (MPNs) without Philadelphia chromosome justifies their further investigation as a model of the clonal evolution of myeloid malignancies over prolonged periods of time (1). The three major entities, essential thrombocythemia (ET), polycythemia vera (PV), and primary myelofibrosis (PMF), are driven by mutations in just three genes: *JAK2*, *CALR*, and *MPL*. These mutations originate at the level of hematopoietic stem cells (HSCs) but, depending on the intrinsic and extrinsic factors, can lead to differential skewing of hematopoiesis predominantly into one of the myeloid lineages presenting clinically with one of the three phenotypes (2). In some cases, these mutations appear early in ontogenesis and are also prevalent in an asymptomatic state of clonal hematopoiesis of indeterminate potential (CHIP), which may evolve into overt malignancy over a long period of time (3). The distribution of MPN-associated mutations in CHIP is not identical to that in MPNs suggesting that different mutations lead to different evolutionary trajectories (4–9). The putative role of immune surveillance as a significant contributor to early oncogenesis in MPNs is becoming increasingly recognized (10). The proposed pathways for its evasion include downregulation of genes from the antigen processing and presentation pathway through human leukocyte antigen (HLA) class I and II pathways (11) and upregulation of negative immune checkpoint ligands (12, 13). In addition, we showed that there might exist HLA class I (*HLA-I*) alleles that are protective against the development of JAK2 V617F-driven MPNs (14). Other groups reported similar observations on HLA allele distribution in CALR mutated (CALRmut) MPNs (15). However, some reports questioned whether JAK2 V617F and CALRmut-derived neoantigens can be presented in MPN patients at all (16), although T cells specific for such neoantigens are consistently identified in healthy individuals (17). All these observations suggest that immunogenetic factors contribute significantly to the pathogenesis of MPNs in a driver mutation-dependent manner, but the precise mechanisms of immunoediting require further investigations.

Therefore, the primary objective of our study was to investigate the distribution of *HLA-I* and *-II* alleles in CALRmut+ MPN patients from the Bulgarian population and to provide additional evidence for HLA-mediated restriction of CALRmut-driven oncogenesis. Furthermore, any findings from Bulgarian patients were intended to be compared with those from previous studies and to be further fostered by molecular and bioinformatic analyses, which could support the hypothesis of HLA-mediated restriction. The secondary objectives of our work are to analyze *HLA-I* and *-II* pathway genes in blood cells harboring CALR mutation, as downregulation of those pathways had been recognized as one of the main mechanisms for immune escape by cancer cells.

## 2 Materials and methods

### 2.1 Enzyme-linked immunosorbent spot (ELISpot) assay

Functional *in vitro* measurements of antigen-specific Т-cell immune response against CALR and JAK2 mutant proteins were performed by ELISpot method using Interferon-γ ELiSpot Basis kit (Autoimmune Diagnostika, GmbH, Germany). Peripheral blood lymphocytes were isolated by gradient centrifugation on Pancoll separation medium (PAN Biotech, GmbH, Germany) and adapted in Panserin 413 media (PAN Biotech, GmbH, Germany) to a concentration of 2 × 10^6^/ml. Two peptides containing JAK2 V617F (LVLNYGVCF and VLNYGVCFC) and CALRmut (RMMRTKMRM and SPARPRTSC), used in HLA-epitope binding, were selected for ELISpot assay. Briefly, 100 μl of the cell suspension (2 × 10^5^ cells) was distributed into the test plate fitted with membranes coated with anti-human IFN-γ antibody and mixed with 100 μl of positive control (Pokeweed mitogen), negative control (medium), or each of the selected peptides at a concentration of 2 μg/ml assayed in duplicate. The peptide concentrations were determined in preliminary experiments with cells from healthy individuals expressing HLA-A*02:01 and B*35:01 using three peptide concentrations—1, 2, and 4 μg/ml. After incubation for 48 h at 37°C in a CO_2_ (5%) incubator, the plate was washed with washing buffer, and 100 μl of solution of diluted secondary antibody was added to each well of the plate. After 2 h of incubation at 37°C and six rinses with washing buffer, 100 μl of substrate was added to each well. After a 20-min incubation, the reaction was terminated by three washes with distilled water. An EliSpot reader and microscope were used to read and record the reaction.

### 2.2 *In vitro* HLA:peptide binding assay

*In vitro* affinity of peptide binding to HLA conformers was determined by Immunitrack (Denmark, acquired by Eli Lilly and Company as of 2 October 2023) using their proprietary MHC:peptide binding assay (18). The assay was performed for the following HLA alleles: A*02:01, B*07:02, B*08:01, B*35:01 complexes, and the following two CALRmut-derived peptides: SPARPRTSC and RMMRTKMRM. The positive control for each assay was a pool of reference peptides, which were known binders to the HLA molecules of interest as follows: A*02:01—LLFGYPVYV (HTLV_Tax), B*07:02—APRTLVYLL (HPV), B*0801—ELRRKMMYM (CMV), and B*35:01—LPFEKSTVM (influenza virus). Assays were run in duplicates in two independent experiments. Results were presented as luminescent intensity over a range of peptide concentrations and affinity was estimated where possible.

### 2.3 Genomic DNA samples

Genomic DNA was extracted from peripheral whole-blood samples obtained from Bulgarian patients with known or suspected MPN referred to our clinical departments for diagnostic and clinical management. Most patients were included in our previous studies on MPNs (14, 19, 20). Genomic DNA samples from healthy controls from the Bulgarian population (n = 1,083), participating in our previous studies (14, 21), were used. Demographic and clinical characteristics of the patients and healthy controls are presented in **Supplementary Table 1** and **Supplementary Figure 1**. Immunogenetic testing for this study was approved by the Local Ethics Committee at Medical University Sofia, Bulgaria. All patients provided informed consent for genetic testing as part of the institutional review boards’ approved standard operating procedures. The principles of the Declaration of Helsinki were strictly followed during the study.

### 2.4 *JAK2* exon 14 and *CALR* exon 9 genotyping

All patients included in the study were tested for JAK2 V617F and CALR mutations through various assays as described previously (22–24). Presence of all mutations was confirmed using direct Sanger sequencing as described previously (23, 24).

### 2.5 *HLA* classes I and II genotyping procedure

*HLA-I* and *-II* genotyping, covering loci *HLA-A*, *-B*, *-C*, *–DPB1*, *–DQA1*, *–DQB1*, and *–DRB1* of all patients and healthy controls, was performed through next-generation sequencing (25) using Holotype HLA™ kit (Omixon, Budapest, Hungary) as described previously (14, 20, 21). Sequencing was performed on the MiniSeq sequencher (Illumina, San Diego, CA, USA). HLA genotype assignment was performed using HLA Twin™ software (Omixon, Budapest, Hungary) (14, 20).

### 2.6 *HLA* class I genotype data from other studies

We retrieved *HLA-I* genotype data for MPN from two other previously published studies. Schischlik et al. provided *HLA-I* genotypes for 42 and 67 CALRmut+ and JAK2 V617F+ Austrian and Italian MPN patients, respectively (26). Gigoux et al. provided *HLA-I* genotype data from a Danish cohort of MPN patients [CALRmut+ (n = 29) and JAK2 V617F+ (n = 149)] and from two Northeastern US MPN cohorts from two academic centers [CALRmut+ (n = 25) and JAK2 V617F+ (n = 105)] (15). HLA evolutionary divergence (HED) and patient harmonic mean best rank (PHBRs) for these patients as well as for the subjects from the Bulgarian cohort were calculated using the procedures described below.

### 2.7 *HLA* genotype data from 1000 Genomes Project

High-resolution *HLA-I* and *-II* data were obtained for 2,693 individuals typed as part of the 1000 Genomes Project (https://www.internationalgenome.org/category/hla/) (27). We filtered out individuals with missing or ambiguous HLA genotypes and ended up with 2,618 subjects for subsequent analyses. HED and PHBRs for those subjects were calculated using the procedure applied to the Bulgarian cohort as described below.

### 2.8 Estimation of HLA evolutionary divergence (HED) for each subject

HED is a measure of the structural divergence of HLA alleles in heterozygous individuals; by definition, it is zero for homozygous individuals. Individuals with higher values of HED have more structurally divergent HLA molecules encoded by heterozygous HLA loci. Therefore, the higher the HED, the more likely it is that the individual can present more antigens through the HLA molecules encoded by a given locus. Tumors in individuals with higher HEDs can potentially be more immunogenic, as evidenced recently by the reported better response to immune checkpoint inhibitors in patients with higher HED values for some alleles (28). HED for each HLA-I and -II locus was estimated as described originally by Pierini et al. (29). Amino acid sequences for each allele required for the implementation of the procedure were retrieved from the IMGT database (14, 20). The Grantham distance (30) between the alleles of each patient was calculated using the Perl script developed by Pierini et al. (29) and available at https://sourceforge.net/projects/granthamdist/. The mean HED for HLA-I and HLA-II for each subject was calculated as the mean of the HEDs of the three HLA-I loci and the four HLA-II loci of the same subject, respectively (14, 20).

### 2.9 Estimation of allele binding best rank (BR) and patient harmonic mean best rank (PHBR) for HLA-I

We generated the sequences of all possible octa-, nona-, deca-, and endecamers from the common neomorphic CALR C-terminus RRMMRTKMRMRRMRRTRRKMRRKMSPARPRTSCREACLQGWTEA (n = 142). The identical procedure for all peptides (n = 38) harboring V617F mutation had already been described (14). The set of peptides for each type of mutation was subjected to antigen-binding prediction by each of the identified HLA-I alleles using NetMHCpan 4.1 server (31). The lowest binding rank obtained for each locus was assigned as the best rank (BR) for binding either CALRmut or JAK2 V617-derived neoantigens. Patient harmonic mean best rank (PHBR) for binding either CALRmut or JAK2 V617-derived neoantigens was calculated as the harmonic mean of the BRs for all six alleles of each subject for the respective type of mutation (14, 32).

### 2.10 *HLA* allele and haplotype association analyses

*HLA-I* and *-II* allelic association with CALR mutational status analyses were performed by fitting additive generalized linear models with sex and age as covariates. Analyses were performed independently for each locus using two-field resolution. The procedure was implemented using *midasHLA* package (33) for Bioconductor for R for Windows (version 4.3.0). Only alleles that passed the Hardy–Weinberg equilibrium test at a p-value not higher than 0.1 and with a frequency of at least 2% were used for fitting the linear models. This pre-processing procedure was also implemented using the *midasHLA* package. Custom annotation of each HLA-I locus as strong binder (SB), weak binder (WB), or non-binder (NB) for either CALR or JAK2 V617F mutation was performed using the standard thresholds proposed by the NetMHCpan 4.1 server for binding ranks of 0.5% and 2% (31). Association analyses using custom annotation of alleles did not exclude any alleles. Haplotype association analyses for bi-, tri-, and quadruple *HLA-I* or *HLA-II* loci were performed using the *haplo.stats* package (https://cran.r-project.org/web/packages/haplo.stats/index.html) through implementation of imputed haplotypes score calculation (14, 20, 34). p-Values below 0.05 were considered significant.

### 2.11 Neoepitope evaluation

Peptide-binding predictions were performed using NetMHCpan 4.1 server (31). Nonamers obtained from common CALRmut sequence were also tested for predicted binding to HLA-A*02:01, HLA-B*35:01, HLA-C*07:02, and HLA-B*08:01 using the SYFPEITHI database (www.syfpeithi.de) (35). NetMHCIIpan 4.1 server was used to predict binding of 15-mer peptides from the CALRmut to selected HLA-II complexes (31). Stability of HLA-peptide-binding prediction was performed using NetMHCstab 1.0 server (36). Proteasomal cleavage prediction was performed using NetChop 3.1 server (37). Prediction of peptide binding to transporter associated with antigen processing (TAP) protein complex was performed by TAPPred server (38).

### 2.12 Molecular dynamics simulations

Molecular dynamics simulations (MDSs) of the HLA-B*35:01, HLA-A*02:01, HLA-B*07:02, and HLA-B*08:01 in complex with two CALRmut-derived nonapeptides (SPARPRTSC or RMMRTKMRM) were performed using AMBER03 force field in GROMACS 2021.3 software (39). MDSs for HLA-C*06:02 were performed for one nona- (RMMRTKMRM) and one decapeptide (RRMMRTKMRM). All initial crystal structures were obtained from Protein Data Bank under the following accession numbers 1A9B (40), 2GTZ (41), 6UJ7 (42), 7NUI, and 5W6A (43) for HLA-B*35:01, HLA-A*02:01, HLA-B*07:02 and HLA-B*08:01, and HLA-C*06:02, respectively. The systems studied were constructed by modifying the control peptide in the X-ray structure with the peptides of interest using the program PyMOL 2.5.1 (44).

Each system was solvated in transferable intermolecular potential with 3 points (TIP3P) water molecules and brought to an electroneutral state by addition of Na^+^ and Cl^−^ ions for every charged amino acid residue in the model system. The correct protonation states of protein internal and surface residues at pH = 7 were estimated using the PROPKA server (https://www.ddl.unimi.it/vegaol/propka.htm) (45). In addition, 150 mM NaCl was added to mimic physiological conditions. After minimization and heating to a temperature of 310 K in constant temperature, constant volume (NVT) ensemble for 400 ps, subsequent production simulation was conducted in isothermal–isobaric (NPT) ensemble. The temperature and pressure were kept at 310 K and 1 atm, respectively.

For all simulations, production phases lasted for 500 ns, and snapshots of the structures were taken at every 20 ps. Root mean square deviations (RMSDs) of atomic coordinates of all atoms of the complex constituents were calculated at every time-point with respect to the initial structure from the production phases using the built-in tool of GROMACS.

### 2.13 Gene expression analyses

Single-cell RNA sequencing (scRNA-Seq) data from CALRmut+ MPN patients were obtained from Gene Expression Omnibus (GEO) deposited under the following accession number GSE117826. The dataset contains processed scRNA-Seq data from bone marrow mononuclear cells (BMMCs) of five CALRmut+ ET patients and from peripheral blood mononuclear cells (PBMCs) of five CALRmut+ MF patients processed using 10× Genomics protocol and sequenced on Illumina HiSeq 2500 machine as described in the original publication (46). The original study also performed targeted single-cell genotyping, so the CALR mutational status was available for all cells subjected to scRNA-Seq. Gene count data for ET and MF patients were analyzed separately as described here in brief. Initially, downloaded gene count data were merged into a Seurat object, and cells were annotated for CALR mutational status. The merged Seurat object was filtered for low-expressing cells (transcript count below 500) and high percentage of mitochondrial gene transcripts (above 10%). The filtered Seurat object was subjected to standard preprocessing steps for normalization and dimensionality reduction using the Uniform Manifold Approximation and Projection (UMAP) approach and integration to correct for batch effects in sequencing as described previously for Seurat objects (47). All steps were performed using standard *Seurat* package functions. Integrated Seurat objects were annotated for cell identity using *celldex* and *SingleR* packages with reference expression data for hematopoietic cells from a previous publication (48). Enrichment of MHC class I and II antigen processing and presentation pathways in individual cells was analyzed using the *AUCell* package (49). The gene sets used were obtained from Kyoto Encyclopedia of Genes and Genomes (KEGG) (50) as described previously (14, 20). Areas under the curves (AUCs) for MHC-I and MHC-II pathways were compared between CALRmut and wild-type (CALRwt) cells per hematopoietic cell type. Differential expression of *MHC-I* and *MHC-II* pathway genes was compared by dotplots implemented through *SCPubr* package. Subsequently, pseudobulk-based analysis of differential gene expression was performed per cell type using the standard *limma* approach (51).

GSE173805 dataset contained RNA-Seq data from mouse BaF3 cell line expressing human *MPL* gene (BaF3-MPL) (52). BaF3-MPL cells were further transduced with lentiviral constructs of human *CALRdel52*, *CALRins5*, and *CALRwt* genes. Gene expression for each of these cell lines was analyzed in technical triplicates in the presence of IL-3 (unstarved conditions) and after 24-h deprivation of IL-3 (starved conditions) (52). Processed gene-level counts were downloaded from GEO portal. The expression matrix was converted to a *DGEdge* object with *DGedge* package, and genes with low expression were filtered out, and expression was normalized using *DGEdge* standard approach. Expressions were subsequently *voom*-transformed using *limma* package for Bioconductor (51). Differentially expressed genes were annotated in regard to their association with the mouse MHC antigen processing and presentation pathway list from KEGG. P-values were corrected according to the Benjamini–Hochberg procedure, and adjusted p-values below 0.05 were considered significant.

GSE195705 dataset contained RNA-Seq data from human umbilical cord blood (UCB) hematopoietic stem cells with CRISPR/Cas9 and AAV6-mediated knock-in approach to introduce *CALRdel52* or *CALRins5* mutation at the endogenous locus (53). Raw transcriptomic data were downloaded from SRA repository under the accession number PRJNA801866. They were aligned to the reference human genome hg38 build using HISAT v. 2.2.1 (54) and annotated with featureCounts v. 2.0.3 (55). All samples were used to build a DGEdge object with *DGedge* package, and genes with low expression were filtered out, and expression was normalized using *DGEdge* standard approach. Subsequent steps were identical to the approach described for the GSE173805 dataset above.

gene expression profiles of sorted bone marrow and peripheral blood cells were obtained from HemaExplorer project (56) and downloaded as log2-transformed values from the BloodSpot 3.0 web-server (www.bloodspot.eu) (57).

The overall analytical approach of the study is summarized in **Supplementary Figure 2**.

## 3 Results

### 3.1 Specific T cells for CALRmut neoantigens are identifiable in healthy individuals but not in MPN patients

**Figure 1:**
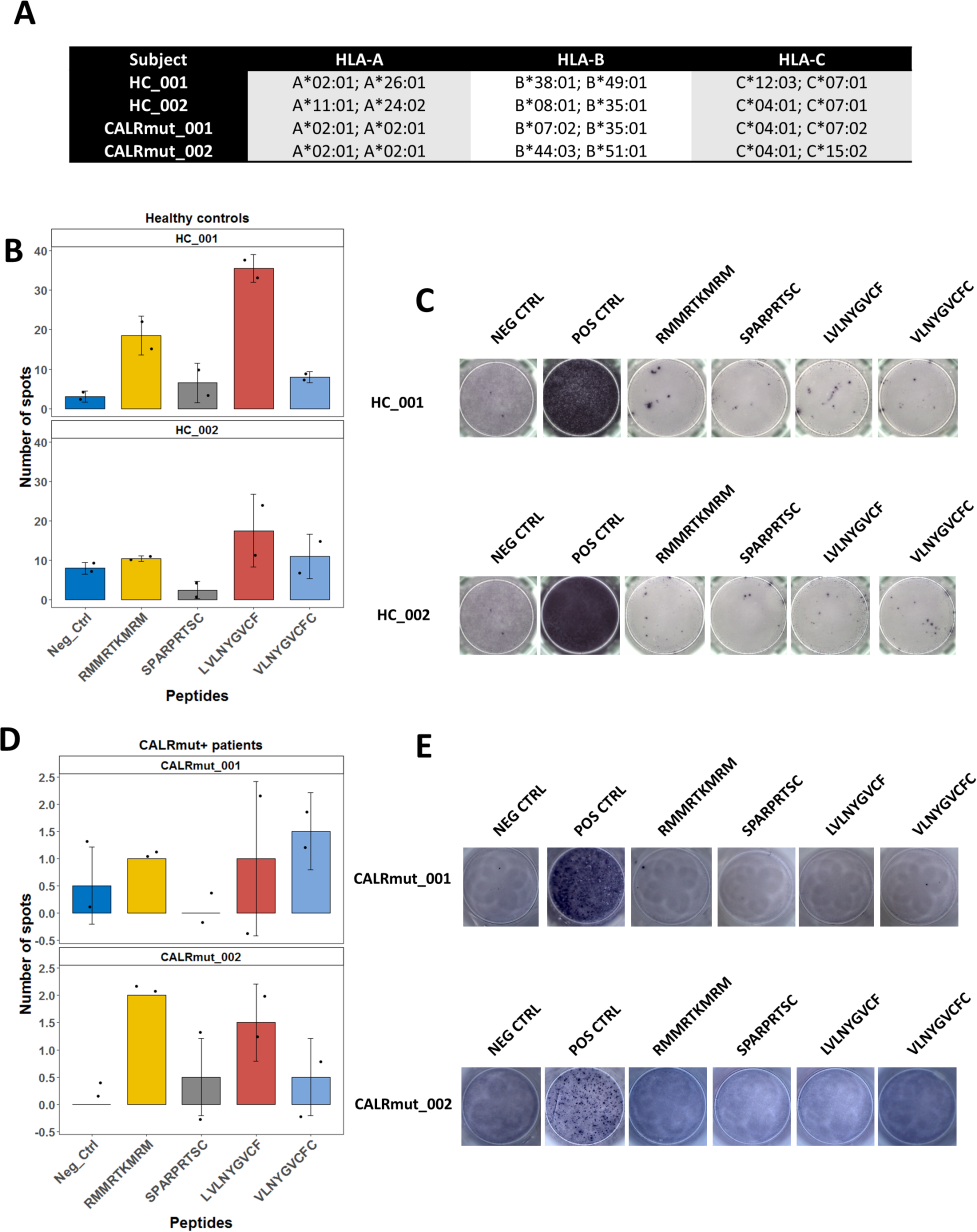
ELISpot assays for detection of neoantigen specific T cells in healthy controls and MPN patients with CALR mutation. VLNYGVCFC and LVLNYGVCF peptides are derived from JAK2 V617F sequence and SPARPRTSC and RMMRTKMRM peptides are derived from the common neomorphic C-terminus of CALR. Negative control was stimulation with the vehicle DMSO. Positive control involved stimulation with pokeweed mitogen. A total of 2 × 10^5^ PBMCs were assayed per well. **(A)** *HLA-I* genotype of the tested subjects. **(B)** Mean ± standard deviation (SD) of detected spots in two wells per sampled subject–peptide combination for healthy controls. Positive results were assumed if the mean per sampled combination was above the SD of the negative control for that patient, and the absolute value of the mean was above 10 spots. **(C)** Demonstration of the selected wells for the assays described in **(B)**. **(D)** Mean ± standard deviation (SD) of detected spots in two wells per sampled subject–peptide combination for CALRmut+ MPN patients. Positive results were assumed if the mean per sampled combination was above the SD of the negative control for that patient, and the absolute value of the mean was above four spots. **(E)** Demonstration of the selected wells for the assays described in **(D)**.

As the JAK2 V617F mutation, and more rarely CALR mutations, can be identified in healthy individuals with CHIP, we questioned whether T cells specific for some CALRmut and JAK2 V617F neoantigens could be identified in randomly selected healthy individuals. We tested two CALRmut-derived nonapeptides (SPARPRTSC and RMMRTKMRM) and two JAK2 V617F-derived nonapeptides (LVLNYGVCF and VLNYGVCFC) for their ability to produce positive results in an IFN-γ-releasing (ELISpot) assay with freshly isolated non-expanded PBMCs. CALRmut-derived peptides were selected based on previous reports (17, 58) and predictions using both NetMHCpan 4.1 (**Supplementary Figure 3**) and SYFPEITHI (**Supplementary Table 2**). JAK2 V617F-derived peptides (LVLNYGVCF and VLNYGVCFC) were selected based on data from our previous study (14), and their predicted binding ranks are presented in **Supplementary Figure 4**. Furthermore, we found that CALRmut peptides were likely to be generated through proteasomal cleavage based on the results from the NetMHCchop 3.0 server (**Supplementary Tables 3**, **4**) and could potentially bind TAP transporter proteins as predicted by the two methods implemented by the TAPPred server (**Supplementary Tables 5**, **6**). Analogous bioinformatics evidence had already been presented for JAK2 V617F-derived peptides in our previous work (14), Collectively, all bioinformatic analyses suggested that the four selected peptides satisfied the theoretical prerequisites for neoantigens. Previous studies reported ELISpot assays for the identification of neoantigen-specific T cells in MPN patients using approaches such as incubation with neoantigen-pulsed dendritic cells (59) or initial induction of neoantigen-reactive T cells by stimulation with cytokines (17). In contrast to these studies, we directly incubated PBMCs with peptides of interest. We assayed healthy controls and CALRmut+ patients with diverse *HLA-I* genotypes (**Figure 1A**). As shown in **Figures 1B, C**, healthy controls showed presence of spots after treatment with LVLNYGVCF and RMMRTKMRM, but CALRmut+ patients showed no reactivity in those assays (**Figures 1D, E**). We additionally tested, using the same assay, six JAK2 V617F+ patients (**Supplementary Figure 5A**). JAK2 V617F+ patients did not demonstrate convincingly any reactivity (**Supplementary Figures 5B, C**).

Although limited in scope and testing patients with diverse *HLA-I* genotype, we obtained evidence for the presence of specific T cells for neoantigens derived from CALRmut and JAK2 V617F mutant peptides in healthy individuals but not in either subgroup of MPN patients. Moreover, as we used 9-mer peptides in our ELISpot assays, the specific T cells in healthy individuals were expected to be most likely HLA-I-restricted, i.e., CD8+ T cells. Collectively, our observations suggested that CALRmut is indeed immunogenic and that specific potential neoantigens can be identified. We therefore decided to test if our selected CALRmut-derived neoantigens could bind specific HLA-I molecules *in vitro* using a standardized assay.

### 3.2 Binding assays

**Figure 2:**
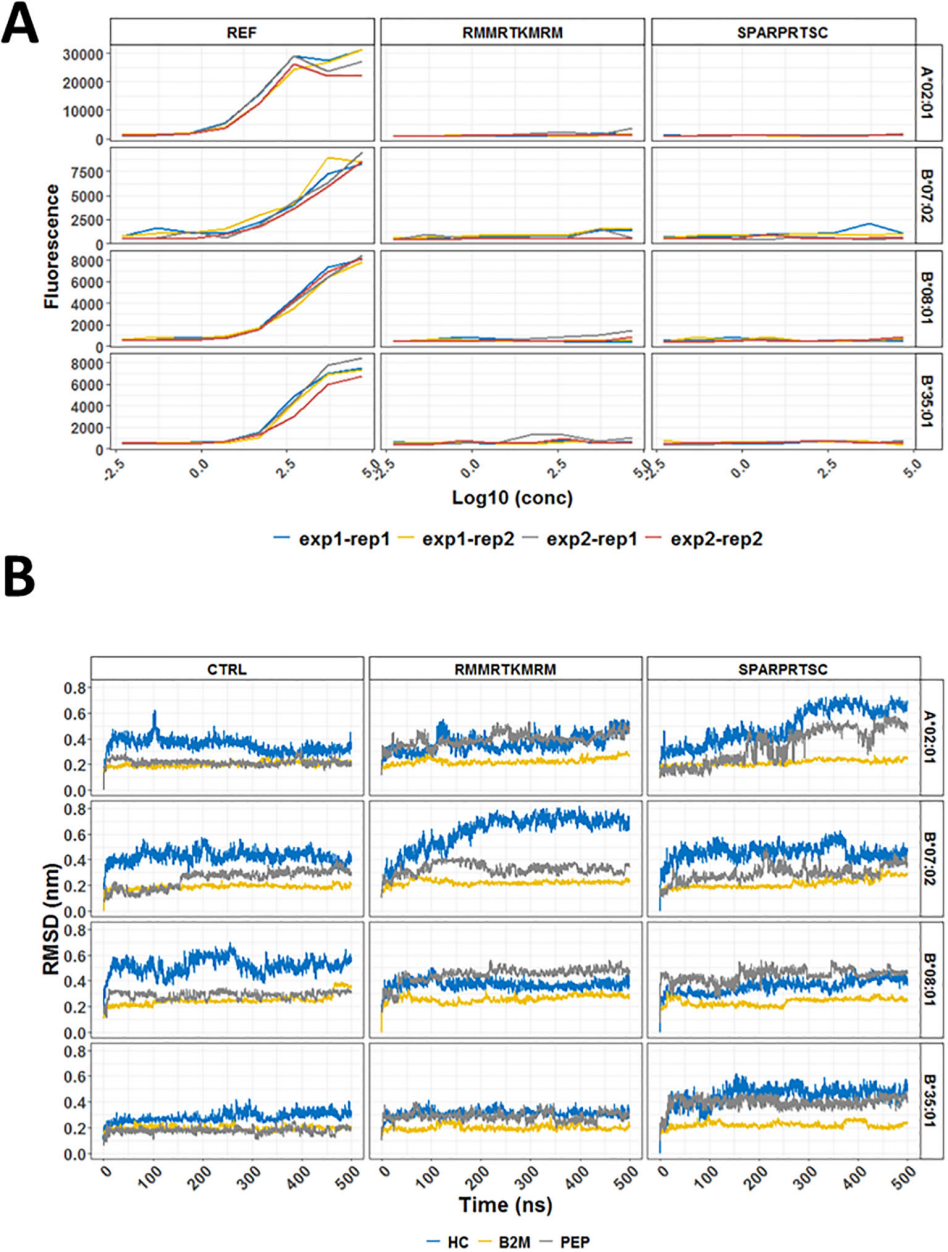
*In vitro* binding and molecular dynamics simulations of selected HLA conformers and CALRmut-derived peptides. **(A)** Binding curves for two 9-mer peptides (RMMRTKMRM and SPARPRTSC) and a pool of reference peptides (REF) with four common human HLA-I molecules (A*02:01, B*07:02, B*08:01, and B*35:01). The assays were performed in two independent experiments (exp1 and exp2) at four different concentrations in duplicates (rep1 and rep2) for each measurement. **(B)** Molecular dynamics simulations of two CALRmut-derived 9-mer peptides (RMMRTKMRM and SPARPRTSC) binding to HLA-A*02:01, HLA-B*07:02, HLA-B*08:01, and HLA-B*35:01. Dynamics of RMSDs of all atoms over 500 ns of simulation of HLA molecules conformers in complex with either a control peptide or any of the two CALRmut-derived peptides. RMSD, root mean squared deviation; PEP, peptide; nm, nanometers; ns, nanoseconds.

We were able to test if the two peptides (RMMRTMRM and SPARPRTSC) used in our ELISpot assays bound to any of the four common HLA-I alleles such as A*02:01; B*07:02; B*08:01, and B*35:01 (**Figure 2A**). This binding assay did not reveal any measurable binding affinity for either of the two peptides to any of the four tested HLA-I conformers. These findings suggested that *in vitro* binding assays neither recapitulated the predicted binding ranks nor directly correlated with data for *ex vivo* immunogenicity and therefore could not be used as the sole evidence for evaluating the immunogenicity potential of neoantigens.

### 3.3 Molecular dynamics simulations (MDS)

*In vitro* binding of peptides to HLA-I conformers does not necessarily mimic the true ability of a given HLA-I molecule to bind a specific peptide because the cell loading of peptides on HLA-I molecules is facilitated by the peptide loading complex (PLC). However, once loaded, a given peptide may remain stably bound or unstably bound with rapid dissociation from the HLA:peptide complex. A rough idea of the stability of binding can be obtained through bioinformatic prediction with NetMHCstab 1.0 server. As shown in **Supplementary Table 7**, SPARPRTSC had significantly longer half-life for binding to B*07:02 than to A*02:01 and B*35:01. To explore this prediction further, MDS was performed using deposited structures of crystallized HLA alleles with specific strong binding proteins. The binding proteins in each deposited structure were exchanged for the primary peptide structures of our interest and modeled for a total of 500 ns as described in the Materials and methods section. The productive simulations of the crystallographic structures with the control peptides at 310 K show that the temperature most strongly affects the heavy chain of В*08:01, where displacement between the corresponding atoms in all structures being compared reaches an average of 0.5–0.6 nm (**Figure 2B**). There are also non-negligible changes in the crystallographic structures B*07:02 (RMSD approximately 0.4 nm) and A*02:01 (RMSD approximately 0.3 nm). Minor changes from the crystallographic structure due to temperature are seen in *35:01 (RMSD approximately 0.2 nm). Simulations of the binding of two HLA-I neoepitopes reveal different dynamics in structural changes compared to the original crystallographic data. As expected, the structure of β2-microglobulin and peptide remains minimally changed in all simulations. After exchange of the binding peptides with two selected nonapeptides, namely, RMMRTKMRM and SPARPRTSC, all investigated systems remained bound throughout the simulations. For the complexes with the peptide RMMRTKMRM, a low level of fluctuation can be seen in the B*08:01 and B*35:01 alleles. On the other hand, more than 200 ns time was needed for B*07:02/RMMRTKMRM to reach a plateau with a high level of fluctuation (0.7 nm). The most significant RMSD fluctuations in the positions of all atoms in the heavy chain were observed in the A*02:01/SPARPRTSC complex. Over 250-ns simulation time was required for the complex to relax to the new positions of the atoms. This time was significantly longer than that for the complexes of the same peptide with other studied alleles. The evolution of the number of hydrogen (H–) bonds of all simulated systems was also studied and is shown in **Supplementary Figure 6**. All complexes were strongly H-bonded forming from 4 to 18 H-bonds in the different frames. Collectively, MDS analyses suggested that once loaded in the peptide-binding cleft of the heavy chain, RMMRTKMRM and SPARPRTSC are most likely to remain stably bound to B*08:01 and not to A*02:01. On the other hand, RMMRTKMRM may also be stably bound to B*35:01 but not to B*07:02. Conversely, SPARPRTSC binds with moderate stability to both B*07:02 and B*35:01.

### 3.4 *HLA-I* allele and haplotype associations in Bulgarian population

To test the association of *HLA-I *genotype with the presence of CALRmut, we used *HLA* genotype data from 42 CALRmut+ patients, 158 JAK2 V617F+ patients, and 1,083 healthy controls from the Bulgarian population (**Supplementary Table 1**; **Supplementary Figure 1**). We first analyzed whether the global diversity of *HLA-I* genotype was associated with presence of either CALRmut or JAK2 V617F mutation. To this end, HED for each locus and the mean HED for all three *HLA-I* loci were calculated and compared between patients and healthy controls. Although CALRmut+ patients showed a tendency for higher HED compared to healthy controls and JAK2 V617F+ patients, this difference was not statistically significant (**Supplementary Figure 7**).

**Figure 3:**
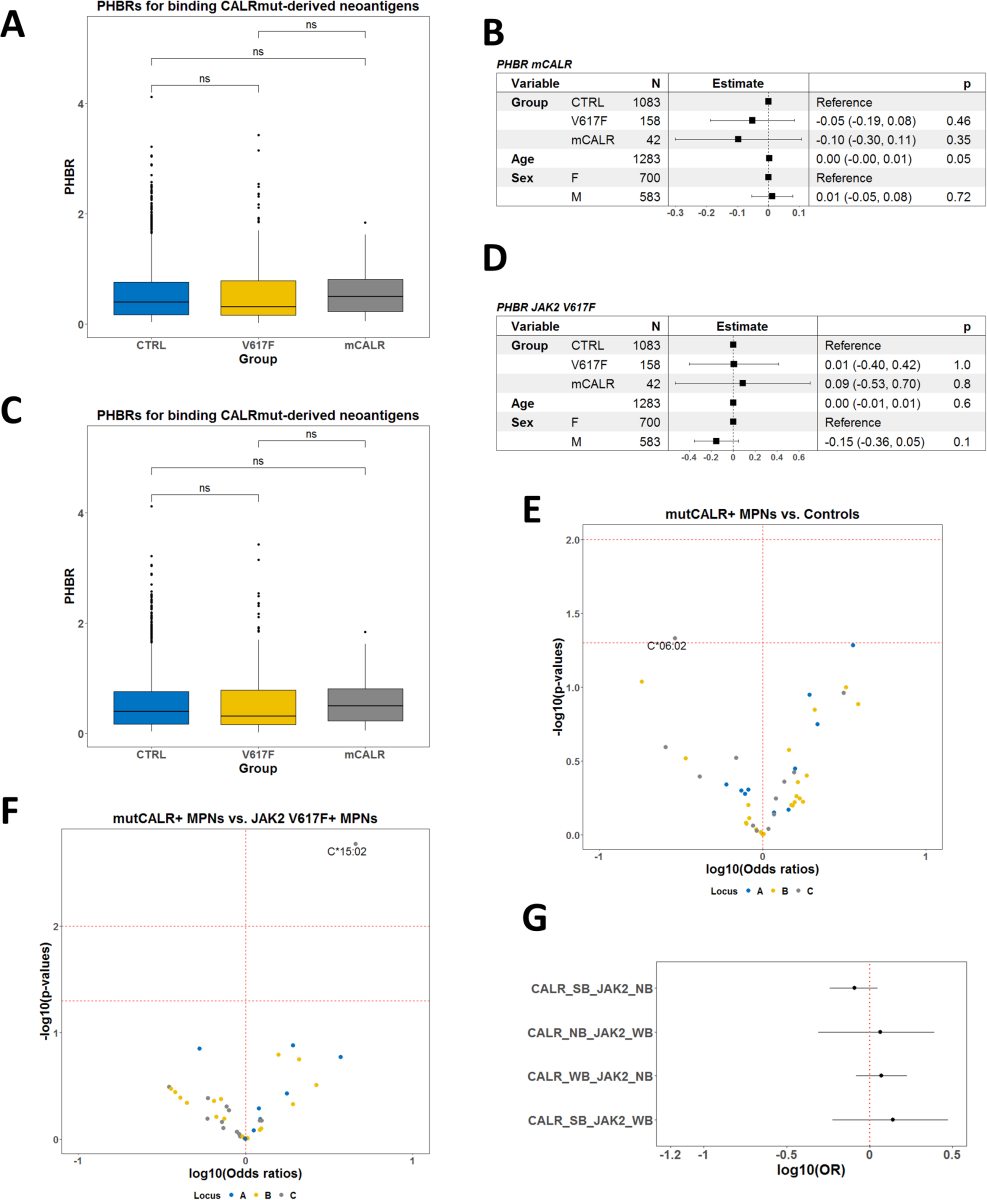
Allele association analyses in Bulgarian CALRmut+ and JAK2 V617F+ MPN patients versus healthy controls. **(A)** Comparison of PHBRs for binding CALRmut-derived neoantigens by groups of subjects and sex. **(B)** Forest plot summarizing the multivariate linear model for PHBR for CALRmut-derived neoantigens with mutational status, age, and sex as covariates. **(C)** Comparison of PHBRs for binding JAK2 V617F-derived neoantigens per groups of subjects and sex. **(D)** Forest plot summarizing the multivariate linear model for PHBR for JAK2 V617F-derived neoantigens with mutational status, age, and sex as covariates. **(E)** Volcano plot summarizing the associations of HLA-I alleles with the presence of the CALRmut *versus* healthy controls. Note the presence of only one potentially protective allele C*06:02. **(F)** Volcano plot summarizing the associations of HLA-I alleles with the presence of the CALRmut versus JAK2 V617F+ MPN patients. Note the presence of only one potentially predisposing allele C*15:02. **(G)** Forest plots using the same dataset as in **(A)** using the custom annotation of alleles. Abbreviations: CALR_SB, allele that strongly binds CALRmut-derived neoantigens; CALR_WB, allele that weakly binds CALRmut-derived neoantigens; CALR_NB, allele that does not bind CALRmut-derived neoantigens; JAK2_WB, allele that weakly binds JAK2 V617F-derived neoantigens; JAK2_NB, allele that does not bind JAK2 V617F-derived neoantigens. p-Values in **(A)** are from a two-sided t-test. p-Value designation: ns—p > 0.05.

Among Bulgarian MPN patients and healthy individuals, we identified a total of 46, 71, and 38 alleles for *A*, *B*, and *C* loci, respectively (**Supplementary Figures 3**, **4**). Their theoretical ability to bind CALRmut-derived peptides was analyzed by NetMHCpan 4.1 server to determine the best binding rank (BR) for each allele as described previously (14). These BRs were used to estimate the patient harmonic mean best rank (PHBR) for binding CALRmut-derived neoantigen. We found that PHBR for CALRmut did not differ between the two groups of patients and healthy controls (**Figures 3A, B**). That was also the case when we compared PHBRs for JAK2 V617F-derived peptides between each of the two groups of patients and healthy controls (**Figures 3C, D**). We further analyzed whether individual *HLA-I* alleles were associated with the presence of CALR mutation. We identified the C*06:02 allele as the only one that was significantly depleted in CALRmut+ patients *versus* healthy controls (p = 0.0464) (**Figure 3E**; **Supplementary Table 8**). There were no alleles that were positively associated with CALRmut+ patients *versus* healthy controls. When we analyzed individual *HLA-I* alleles in CALRmut+ *versus* JAK2 V617F+ patients, we identified that the C*15:02 allele was significantly enriched in CALRmut+ patients (**Figure 3F** and **Supplementary Table 9**). Finally, there was no difference in the distribution of HLA-I alleles between CALRmut+ and JAK2 V617F+ patients when alleles were annotated based on their binding ranks to neoantigens from both mutations (**Figure 3G**). Haplotype association analyses revealed several bi-locus haplotypes that were significantly less frequent in CALRmut+ patients versus healthy controls, such as A*01:01-C*06:02, A*31:01-C:07:01, A*02:01-C*07:01, and B*13:02-C*06-02 (**Supplementary Figures 8**-**10**). Notably, we did not identify any potentially protective tri-locus haplotype (**Supplementary Figure 11**). Of special interest was the identification of protective haplotypes including C*06:02, such as A*01:01-C*06:02 and B*13:02-C*06:02. This observation suggested that if there is a specific *HLA-I* allele with significant protective role against the development of CALRmut+ MPN, the most likely candidate would be C*06:02. Furthermore, additional molecular dynamics simulations of C*06:02 with the control peptide and two CALRmut-derived peptides, RMMRTKMRM and RRMMRTKMRM, showed that the heavy chain of the HLA complex remained relatively stable over 500-ns simulation when in complex with the decapeptide RRMMRTKMRM (**Supplementary Figure 12**) with significant number of H-bonds between the peptide and heavy chain (**Supplementary Figure 13**). This suggested that indeed C*06:02 might have a protective role against the development of CALRmut+ MPN because of its capability to present common neoantigens harboring that mutation.

### 3.5 Meta-analysis of *HLA-I* allele associations

**Figure 4:**
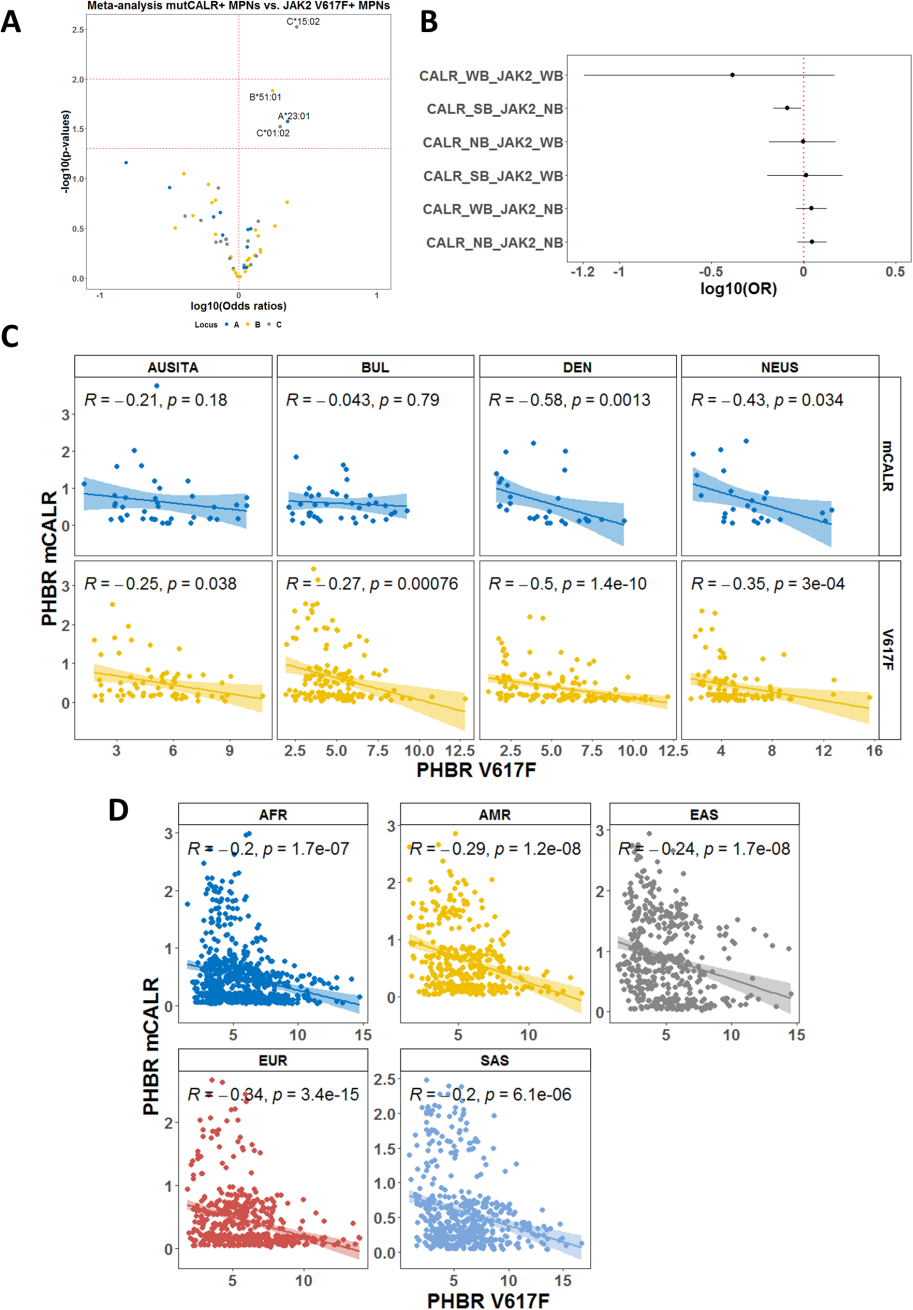
Meta-analysis based on individual HLA-I genotype data. **(A)** Volcano plot representing the association with CALRmut *versus* JAK2 V617F mutation in MPN patients. Analysis was based on data from five independent cohorts as described in the main text. **(B)** Forest plots using the same dataset as in **(A)** demonstrating that alleles that were annotated as strongly binding CALRmut-derided neoantigens but do not bind JAK2 V617F-derived neoantigens are significantly less frequent among CALRmut+ MPN patients. **(C)** Correlation plots between PHBRs for CALRmut and JAK2 V617F in either CALRmut+ or JAK2 V617F+ patients from the cohorts in **(A, B)**. **(D)** Correlation plots PHBRs for healthy individuals from the 1000 Genomes Project. AUSITA, Austria/Italy; DEN, Denmark; NEUS, Northeastern Unites States; BUL, Bulgaria; AFR, Africans; AMR, admixed Americans; EAS, East Asians; EUR, Europeans; SAS, South Asians; CALR_SB, allele that is strongly binding CALRmut-derived neoantigens; CALR_WB, allele that is weakly binding CALRmut-derived neoantigens; CALR_NB, allele that does not bind CALRmut-derived neoantigens; JAK2_WB, allele that is weakly binding JAK2 V617F-derived neoantigens; JAK2_NB, allele that does not bind JAK2 V617F-derived neoantigens. p-Values in **(C, D)** are from two-sided Pearson correlation test.

We proceeded to analyze the associations with individual alleles by comparing CALRmut+ and JAK2 V617F+ patients from independent cohorts. A total of 138 CALRmut+ and 479 JAK2 V617F+ patients from four locations (Austria/Italy, Denmark, Northeastern USA, and Bulgaria) were included. We identified a number of alleles that were more frequently found in CALRmut+ *versus* JAK2 V617F+ patients (C*15:02; C*01:02; B*51:01; A*23:01) (**Figure 4A**). Additionally, *HLA-I* alleles that were predicted to strongly bind CALRmut-derived peptides and not JAK2 V617F-derived peptides were significantly less frequently found in CALRmut+ patients (**Figure 4B**). We further checked for the correlation of PHBR for CALRmut and JAK2 V617F peptides for each subtype of patients in each cohort (**Figure 4C**). Notably, except for CALRmut+ patients from Austria/Italy and Bulgaria, there was a significant inverse correlation between the two types of individual PHBRs. This observation raised the question of whether such an inverse correlation was specific to the *HLA-I* genotypes of MPN patients or if it was a general feature of *HLA-I* genotypes in human populations. To address this question, we obtained the unambiguously typed *HLA-I* genotypes of 2,618 healthy individuals from the 1000 Genomes Project. We calculated the PHBR of each individual from the 1000 Genomes Project for binding either CALRmut-derived or JAK2 V617F-derived neoantigens and tested the correlation between them. As shown in **Figure 4D**, there was a significant inverse correlation between the two PHBRs for all five super regions represented in the 1000 Genomes Project. Overall, these analyses show that in both MPN patients and healthy individuals, the human genome exhibits differential capability to present neoantigens from either CALR mutations or JAK2 V617F mutations.

### 3.6 *HLA-II* allele and haplotype associations

Our HLA typing approach provided data regarding *HLA-II* genotypes of Bulgarian patients. Therefore, we compared HED for HLA class II loci between CALRmut+, JAK2 V617F+ MPNs, and healthy controls. The only significant association was that CALRmut+ MPNs had higher HED for the *DQB1* locus in comparison with both JAK2 V617F+ patients and healthy controls (**Supplementary Figure 14A**), which was independent of age and sex (**Supplementary Figure 14B**).

**Figure 5:**
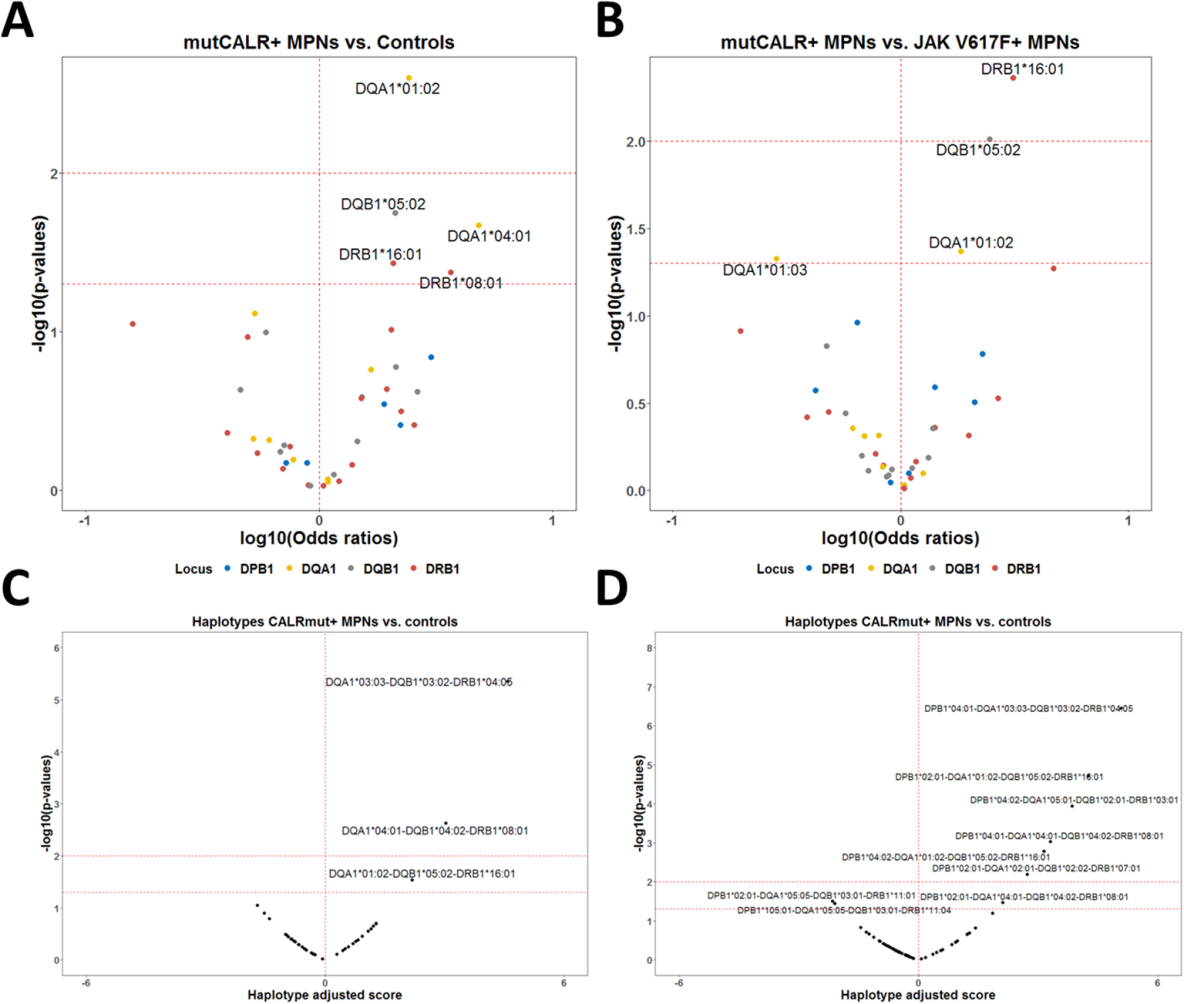
HLA-II allelic and haplotypic associations with CALR mutational status in the Bulgarian population. **(A)** Volcano plot of the HLA-II alleles associated with CALRmut+ MPNs *versus* healthy controls. **(B)** Volcano plot of the HLA-II alleles associated with CALRmut+ *versus* JAK2 V617F+ MPNs. **(C)** Volcano plot of exemplary tri-locus haplotypes associated with CALRmut+ *versus* healthy controls. **(D)** Volcano plot of quadruple-locus haplotypes associated with CALRmut+ *versus* healthy controls.

When we performed the association analysis for *HLA-II* alleles comparing CALRmut+ patients to healthy controls, we identified only alleles that were significantly enriched in CALRmut+ patients (DQA1*01:02, DQB1*05:02, DQA1*04:01, DRB1*16:01, DRB1*08:01) (**Figure 5A**; **Supplementary Table 10**). None of these alleles were predicted to bind the 15-mer peptides derived from CALRmut (**Supplementary Table 11**). Similarly, analysis comparing CALRmut+ to JAK2 V617F+ patients identified three alleles significantly more frequent in CALRmut+ patients (DRB1*16:01, DQB1*05:02, DQA1*01:02) and one allele that was significantly depleted (DQA1*01:03) (**Figure 5B**; **Supplementary Table 12**). Additional analyses of *HLA-II* haplotype associations (**Figures 5C, D**; **Supplementary Figures 15**, **16**) showed some tri-loci haplotypes predisposing individuals to the development of CALRmut+ MPNs (**Figure 5C**). Interestingly, there were some potentially protective four-loci haplotypes such as DPB1*02:01-DQA1*05:05-DQB1*03:01-DRB1*11:01 and DPB1*105:01-DQA1*05:05-DQB1*03:01-DRB1*11:04 (**Figure 5D**). These data suggested that *HLA-II* alleles may play diverse roles in the development of CALRmut+ MPNs, which warrants further exploration in independent cohorts.

### 3.7 Gene expression profiling

**Figure 6:**
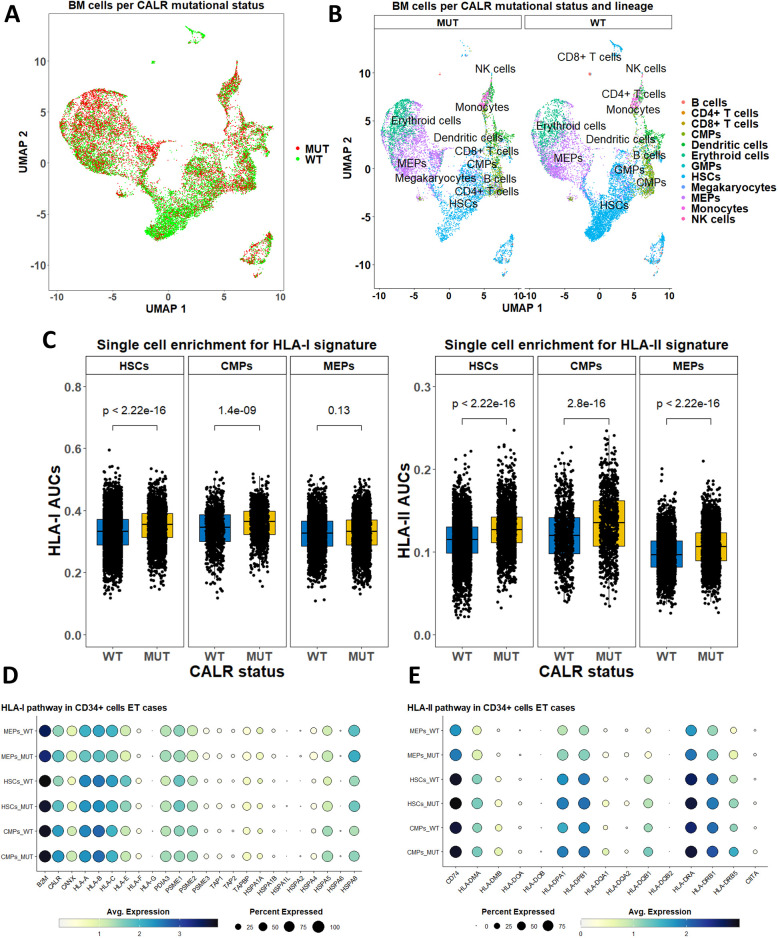
Expression analysis of *HLA-I* and *-II* pathway genes based on publicly available scRNA-Seq data from bone marrow cells in five ET cases. **(A)** Two-dimensional scatter plot after UMAP transformation representing data merged from all cases with CALR mutational status. **(B)** Two-dimensional plots after UMAP transformation representing data merged from all cases and lineage annotation. Plots are split by CALR mutational status. **(C)** Comparison of AUCs for HLA-I (left) and HLA-II (right) pathways in selected cell subtypes based on their CALR mutational status. **(D)** Dot plots summarizing the expression of selected *HLA-I* pathway genes in three cellular subtypes stratified by CALR mutational status. **(E)** Dot plots summarizing the expression of selected *HLA-II* pathway genes in three cellular subtypes stratified by CALR mutational status. p-Values in **(C)** are from a two-sided Wilcoxon test. HSCs, hematopoietic stem cells; CMPs, common myeloid progenitors; MEPs, megakaryocyte-erythroid progenitors.

**Figure 7:**
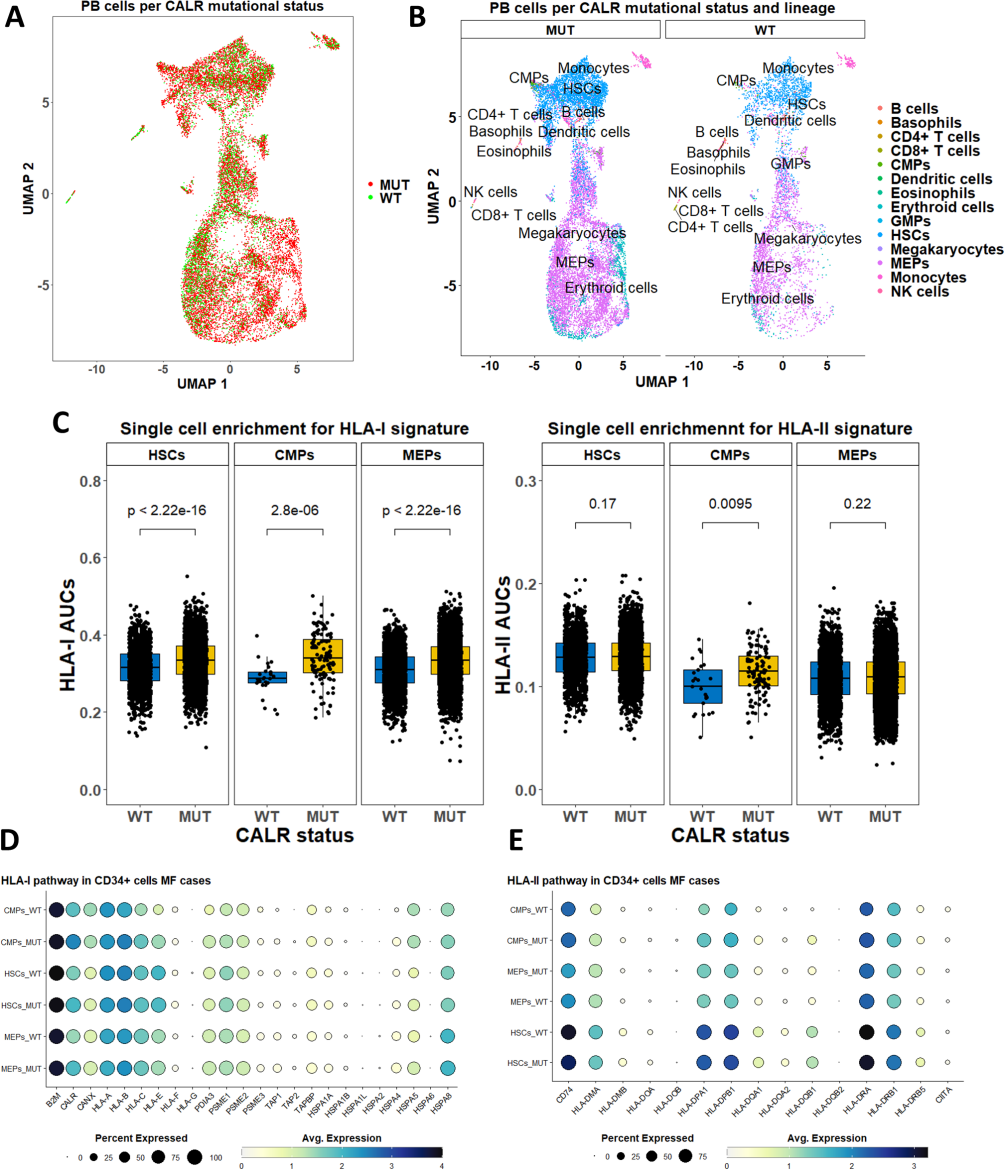
Expression analysis of *HLA-I* and *-II* pathway genes based on publicly available scRNA-Seq data from peripheral blood cells in five MF cases. **(A)** Two-dimensional scatter plot after UMAP transformation representing data merged from all cases with CALR mutational status. **(B)** Two-dimensional plots after UMAP transformation representing data merged from all cases and lineage annotation. Plots are split by CALR mutational status. **(B)** Comparison of AUCs for *HLA-I* (left) and *HLA-II* (right) pathways in selected cell subtypes based on their CALR mutational status. **(D)** Dot plots summarizing the expression of selected *HLA-I* pathway genes in three cellular subtypes stratified by CALR mutational status. **(E)** Dot plots summarizing the expression of selected *HLA-II* pathway genes in three cellular subtypes stratified by CALR mutational status. p-Values in **(C)** are from a two-sided Wilcoxon test. HSCs, hematopoietic stem cells; CMPs, common myeloid progenitors; MEPs, megakaryocyte–erythroid progenitors.

We analyzed scRNA-Seq data with genotyping of the transcriptomes from CALRmut+ ET and MF patients (46) to gain insight into the level of expression of *HLA-I* and *-II* pathway genes (**Figures 6**, **7**). After standard data integration for ET and MF cases separately, we performed single-cell identity annotation using conventional hematopoietic cell expression profiles (48) (**Figures 6A**, **7A**). Analysis of the overrepresentation of *HLA-I* and *-II* pathways was performed using the SCENIC approach (49) as it has been shown to outperform other methods for supervised analysis of gene expression such as single-sample gene set enrichment analysis (GSEA) and gene set variation analysis (GSVA) (60). Comparisons between CALRmut+ and normal cells were performed for each of the annotated cell types. Notably, in ET patients, CALRmut+ hematopoietic stem cells (HSCs), common myeloid progenitors (CMPs), and megakaryocyte–erythroid progenitors (MEPs) showed statistically significant enrichment for both *HLA-I* and *-II* pathway genes (**Figure 6B**). An identical observation was made for CALRmut+ HSCs, CMPs, and MEPs from MF patients (**Figure 7B**). At individual gene level, however, we did not identify any significantly up- or downregulated *HLA-I* or *-II* pathway gene (**Figures 6C**, **7C**). Of note, *TAP1* and *TAP2* genes showed particularly low expression in both CALRmut+ and normal cells (**Figure 7C**). Additionally, *CIITA* showed markedly low levels of expression in all analyzed cell types (**Figures 6D**, **7D**). Because of these observations, we confirmed using data from reference gene expression repositories that indeed *TAP1*, *TAP2*, and *CIITA* genes’ expression is relatively low in the bone marrow hematopoietic and progenitor pools (**Supplementary Figures 17**, **18**). Finally, pseudobulk analysis with aggregation of single-cell gene expressions at patient level did not reveal any significantly up- or downregulated *HLA-I* or *-II* genes in any of the three studied cellular populations (**Supplementary Tables 13**-**18**).

We additionally sought to identify any direct effect of CALR mutation on the expression of *MHC-I* and *-II* pathway genes. To this end, we analyzed scRNA-Seq data from BaF3 cells harboring the human *MPL* gene, which were transduced with CALRmut construct and grown under the presence of IL-3 (unstarved conditions) or in its absence (starved conditions) (52). In this analysis, we did not identify any *MHC-I* or *–II* pathway gene as significantly up- or downregulated in the presence of CALR mutation under any of those conditions (**Supplementary Figures 19**-**21**). Finally, we analyzed scRNA-Seq data from human umbilical cord blood (UCB) HSCs which had CALRmut knock-in using CRISPR/Cas9 gene editing (53). There were unremarkable changes in the *HLA-I* and *-II* genes with the exception of *CALR*, which appeared to be significantly downregulated (**Supplementary Figures 22**, **23**). On the other hand, the *CANX* gene was significantly upregulated in CALRmut+ UCB HSCs (**Supplementary Figure 23**). Collectively, gene expression analyses under various experimental settings suggest that the presence of the CALR mutation may slightly upregulate the overall expression of HLA-I and -II pathways without significantly affecting the expression of any specific gene from either pathway.

## 4 Discussion

Cancer cell populations can escape immune surveillance through three main types of processes: loss of antigenicity, loss of immunogenicity, and induction of an immunosuppressive microenvironment (61). Myeloid malignancies, including MPNs, are usually considered to be poorly immunogenic because of the lower mutational burden. However, a number of studies have shown skewing of the *HLA-I* and *-II* genotypes in patients with JAK2 V617F and CALR mutations suggesting those diseases may undergo active immunoediting during their early development (14, 15), which is consistent with the idea that, in many cases, driver cancer mutations are HLA-I and HLA-II restricted (32, 62).

The standard T-cell epitope prediction approaches we used in this study suggested that the common neomorphic CALR C-terminus could be a source of a number of neoantigens potentially binding to a number of common *HLA-I* alleles. Additionally, *HLA-I* genotype PHBRs are much lower for CALRmut than for JAK2 V617F mutation. Our bioinformatic analysis also showed that CALRmut could be efficiently degraded by proteasome, and the derived peptides could bind transport proteins TAP1 and TAP2. These findings are also supported by existing experimental evidence that mutated CALR C-terminus can be efficiently degraded by the proteasome pathway (63). We, therefore, concluded that CALRmut are likely to lead to the generation of neoantigens *in vivo*, as all the theoretical prerequisites were satisfied (64). However, our study showed the limitations of neoantigen prediction as none of the CALRmut-derived peptides assembled spontaneously to predicted binding HLA-I molecules *in vitro* even though neural network approach implemented through the NetMHCpan 4.1 server still outperforms other similar methods (65). Therefore, any potential identification of putative neoantigens requires verification by mass spectrometry or immunogenicity assays (64). Furthermore, stability, and not binding affinity, is probably more predictive of immunogenicity of neoantigens (64). However, using predicted binding ranks allows for an unbiased estimation of theoretical ability of human genomes to bind specific neoantigens and is well suited for the goals of the current study (64),

Several previous studies demonstrated that CALRmut is immunogenic (17, 58). Of note, Bozkus et al. (17) showed that the nonamer RMMRTKMRM elicited a strong response in healthy subjects who harbored *HLA-I* alleles predicted to have high binding affinity for it. We were also able to demonstrate a T-cell response to RMMRTKMRM in unstimulated PBMCs from at least one healthy individual. As we used only 9-mer peptides, the observed responses were likely HLA-I-restricted, i.e., from CD8+ T cells. Collectively, these findings confirm that CALRmut is not only antigenic but also sufficiently immunogenic at least in people with an intact immune system. However, based on our assay, no definitive conclusions can be drawn regarding the frequency of healthy individuals who may possess existing T cells that recognize CALRmut-derived neoantigens in the context of specific *HLA-I* molecules. To address this question, more extensive testing would be required, including the use of a highly sensitive ELISpot assay on a larger cohort of healthy subjects with diverse HLA-I genotypes across various age groups. Such testing was beyond the scope of our current study, as our primary focus was on obtaining genetic evidence for HLA-mediated immunoediting in CALRmut+ MPNs.

The poor immunogenicity of CALRmut in MPN patients, as evidenced *ex vivo* and *in vivo*, can be explained by a number of non-mutually exclusive mechanisms. Recently, Gigoux et al. (15) showed depletion of some *HLA-I* alleles in CALRmut+ vs. JAK2 V617F+ patients from two cohorts. Notably, Gigoux et al.’s study did not include healthy controls and, therefore, compared only JAK2 V617F+ and CALRmut+ patients. In contrast to Gigoux et al.’s study, our statistical approach was much more stringent and robust, as we included subjects from a single population with a high level of genetic homogeneity. We also excluded rare alleles and those that failed to meet the Hardy–Weinberg equilibrium assumption for the Bulgarian population. Finally, we included age and sex as covariates in the generalized linear models for association analysis. Based on our robust approach, we identified the C*06:02 allele as significantly less frequent in CALRmut+ patients. This allele was identified by Gigoux at al.’s approach (15) as less frequent in CALRmut+ than in JAK2 V617F+ patients and is also a predicted strong binder for some peptides using the NetMHCpan 4.1 server. We were not able to test directly the binding affinity of the C*06:02 to some CALRmut neoantigens, but our MDS analyses suggested that it could form a relatively stable complex with the decamer RRMMRTKMRM over a long period of 500 ns. Furthermore, this peptide was predicted to be generated by proteasomal cleavage and to bind TAP1 and TAP2 proteins suggesting that it satisfies the major requirements for presentation by the specific HLA-I alleles (64).

**Figure 8:**
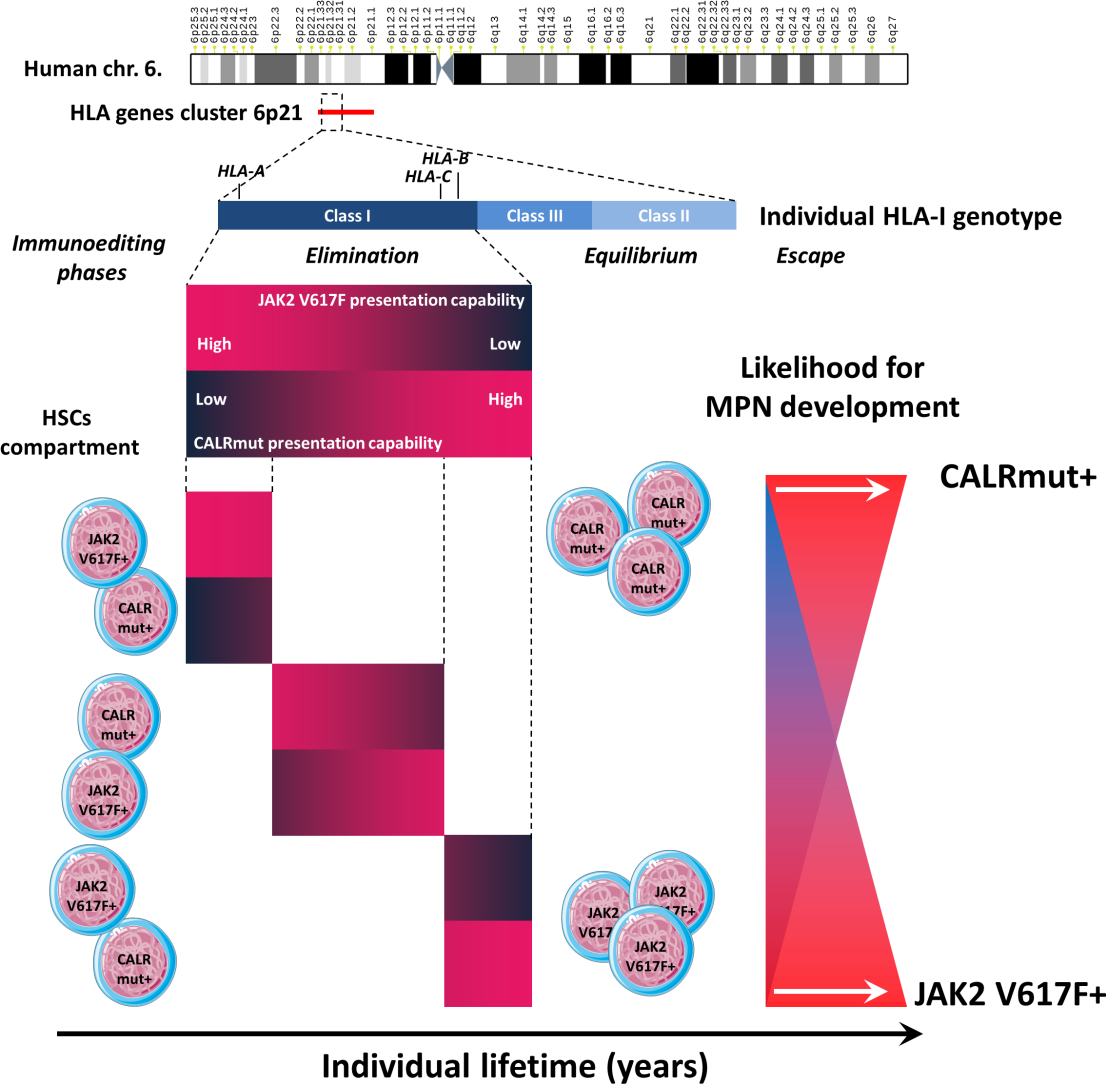
Summary of the main findings. The *HLA* gene cluster is located on human chromosome band 6p21. We specifically evaluated the capability of individual HLA class I genotypes (including the three loci *HLA-A*, *HLA-B*, and *HLA-C*) to present neoantigens derived from either the JAK2 V617F or CALR mutation. We demonstrated that individual *HLA-I* genotypes have divergent capabilities to present either type of neoantigen, which affects the three phases of immunoediting. Two extreme scenarios are depicted. For example, if an individual has a specific *HLA-I* genotype with a low capacity to present JAK2 V617F-derived neoantigens and a low capacity to present CALRmut-derived neoantigens, it is very likely that this would lead to the effective elimination of JAK2 V617F+ but not CALRmut+ cells that eventually occur among the HSC pool. In the equilibrium phase, this would lead to the sustained survival and proliferation of CALRmut+ MPN-SCs, which may eventually acquire additional genetic or epigenetic features leading to additional competitive advantages and the development of overt CALRmut+ MPN. In the opposite scenario, an individual may have an *HLA-I* genotype that can efficiently present CALRmut-derived neoantigens but has a very low capability to present JAK2 V617F-derived neoantigens. In that case, CALRmut-transformed HSCs will be efficiently eliminated, while those that are JAK2 V617F+ will be more likely to avoid early elimination and enter the equilibrium phase. Eventually, they may escape immune response by acquiring additional features leading to overt JAK2 V617F+ MPN. In each scenario, the process may take many years.

To overcome the limitations of small sample sizes, we performed meta-analysis combining patients’ *HLA-I* genotype data from four independent cohorts. It appeared that alleles predicted to be strong binders for CALRmut neoantigens, but not for JAK2 V617F-derived neoantigens, were significantly less frequent in CALRmut+ patients. Notably, PHBRs for CALRmut inversely correlated with those for JAK2 V617F for almost all groups included in the meta-analysis. We confirmed that this dissociation was a general feature of normal human genotypes using the PHBR measures from more than 2,500 individuals from five different geographic regions. Taken together, these data suggest that the individual capability of the *HLA-I* genotype to present either CALRmut or JAK2 V617F neoantigens may restrict differentially the oncogenesis driven by each of these two mutations. This may contribute to the differential kinetics of MPNs mediated by the two types of mutations. While JAK2 V617F mutation (66, 67) is known to appear early during an individual’s life (7, 9), CALR mutations are much less frequent (66, 67). Furthermore, CALR mutations are detected later in life, with an estimated lag in occurrence of 15 years, despite their higher proliferative advantage (8, 68). However, there are prominent examples of CALR mutations originating *in utero*, with evolution to an overt MF over more than three decades (69). Interestingly, Kyllesbech et al. (70) showed that a proportion of healthy volunteers may have detectable IgG antibody levels against CALRmut suggesting that CALR mutation acquisition may not be an extremely rare event. Instead, the mutated cells are either rapidly eliminated or kept in check through immune surveillance for prolonged periods of time. While a main contributor to this differential kinetics might be predisposing germ-line genetic factors (71), with subsequent positive selection for the driver mutation (4–6), one cannot exclude the role of negative immune selection as a contributing factor. It can be proposed that more immunogenic CALR mutations are more efficiently eliminated by the immune system, thereby diminishing the likelihood of identifying such mutations at earlier ages. Furthermore, we showed that the *HLA-I* genomes’ capabilities to present either CALRmut or JAK2 V617F mutation were inversely correlated, which could contribute to their almost canonical mutual exclusivity in MPNs (66, 67) due to the initial restriction of transformed cells (**Figure 8**). Collectively, our findings, in conjunction with other reports, suggest that the two main driver mutations in MPNs are most probably associated with differential trade-offs between hematopoietic fitness and immunogenicity (72, 73) (**Figure 8**).

*CALR* exon 9 mutations in MPNs may directly contribute to immune escape by a number of non-mutually exclusive mechanisms, such as downregulation of HLA-I molecules (74), perturbation of the unfolded protein response (52, 75), and phagocytosis suppression (76). Therefore, we questioned whether CALR mutations affected *HLA-I* pathway genes at transcriptional level. To this end, we compared the expression of those genes in various subsets in the stem and progenitor cell compartments in ET and MF patients using publicly available scRNA-Seq data. As the dataset provided the genotype of each cell, we directly compared gene expression of mutated and normal cells residing in an identical microenvironment. We demonstrated statistically significant increase in the expression of the entire *HLA-I* and *-II* pathway genes for all cell subtypes in both ET and MF patients. However, at the individual gene level, there were no significantly over- or underexpressed genes. Taken together, these data suggested that at transcriptional level, the presence of CALRmut does not impair HLA-I pathway. Notably, based on the scRNA-Seq data, expression of *TAP1* and *TAP2* genes in both wild-type and mutant cells was low. It is unclear, however, whether this limits the capability of HSCs and progenitor cells to present TAP-dependent antigens.

We further identified predisposing *HLA-II* alleles that might predispose to CALRmut+ MPN. This may not be considered surprising as Humblet-Baron et al. (77) showed that CD4+ T cells could play a role in the pathogenesis of myeloproliferative disorders in mice in an MHC-II-dependent fashion. We further showed overall upregulation of *HLA-II* pathway genes in CALRmut+ CD34+ cells at transcriptional level. The role of *CIITA* in regulation of *HLA-II* genes in CALRmut was unclear, as its transcriptional level remained unchanged and low in consistency with a previous report (78). Li et al. also showed that expression of *MHC-II* pathway genes is particularly high in HSCs with long-term repopulating potential (78), which supports our data assuming that CALRmut+ HSCs have a repopulation advantage. Another study suggested that T-regulatory cells (Tregs) play a role in the maintenance of the HSC pool in the bone marrow niche (78). The same study estimated that 9.6% of HSCs made direct contact with Tregs (78). Such contacts are expected to lead to the formation of immunological synapses mediated by the recognition of peptide-loaded MHC-II molecules by cognate TCRs. Therefore, we do not find it surprising that a number of HLA-II alleles were significantly more common in CALR-mut+ MPN patients than in healthy controls. In this regard, it would be interesting to investigate whether CALR-mut+ HSCs form more contacts with Tregs than wild-type HSCs in the bone marrow. Furthermore, our findings of upregulation of HLA-I and HLA-II pathways are also not surprising, as it has already been shown that CALR mutations lead to overactivation of the JAK/STAT signaling pathway, and STAT1 and STAT6 activation have previously been shown to lead to upregulation of MHC-I and MHC-II pathways (79–81).

Finally, the HLA-I-mediated restriction of CALRmut-driven oncogenesis suggests that it might be unlikely to obtain clinically meaningful responses with peptide vaccine based on the mutated CALR sequence. Gigoux et al. (15) reached the same conclusion and tried to optimize their CALRmut vaccine by developing a heteroclytic sequence, which can bind more strongly to A*02:01. We propose that an additional approach, which could be explored, would be testing of endogenous TAP-independent peptides as potential targets for development of vaccines in MPNs (82). An alternative approach could also be to target neoantigens from atypical transcripts or endogenous retroviruses (83, 84). Ideally, any design of neoantigen-based vaccine in MPNs should be supplemented by HLA-I and -II ligandome data (85) potentially in combination with immune checkpoint inhibitors or recombinant interferons.

## Data Availability

The datasets presented in this study can be found in online repositories. The names of the repository/repositories and accession number(s) can be found in the article/Materials and Methods section above.
