# Supplementary material for: Differential modulation of mutant CALR and JAK2 V617F-driven oncogenesis by HLA genotype in myeloproliferative neoplasms
Source: Front Immunol. 2024 Sep 16;15:1427810. doi: 10.3389/fimmu.2024.1427810 (PMC11439724; doi:10.3389/fimmu.2024.1427810)
Supplement: Supplementary file 1 [file DataSheet1.pdf]

Supplementary Material

1 Supplementary Figures and Tables

1.1 Supplementary Figures

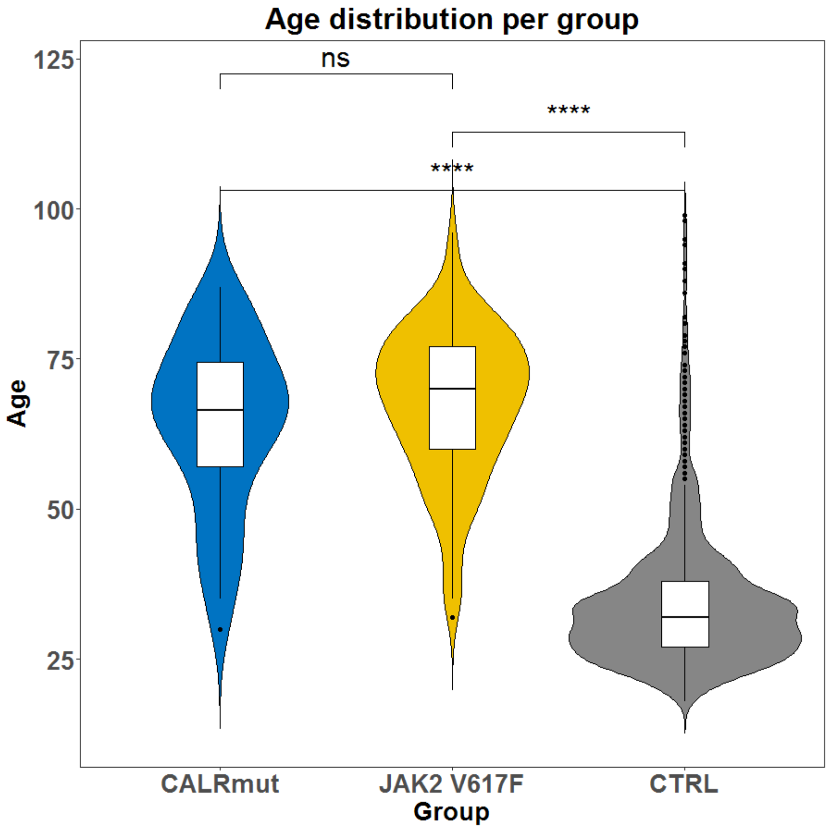

Supplementary Figure 1. Comparison of age distributions in MPN patients and healthy controls within the Bulgarian cohort. P-values are from two-sided t-tests. P-values designation: ns – $p > 0.05$, * – $p \leq 0.05$, ** – $p \leq 0.01$, *** – $p \leq 0.001$, **** – $p \leq 0.0001$.

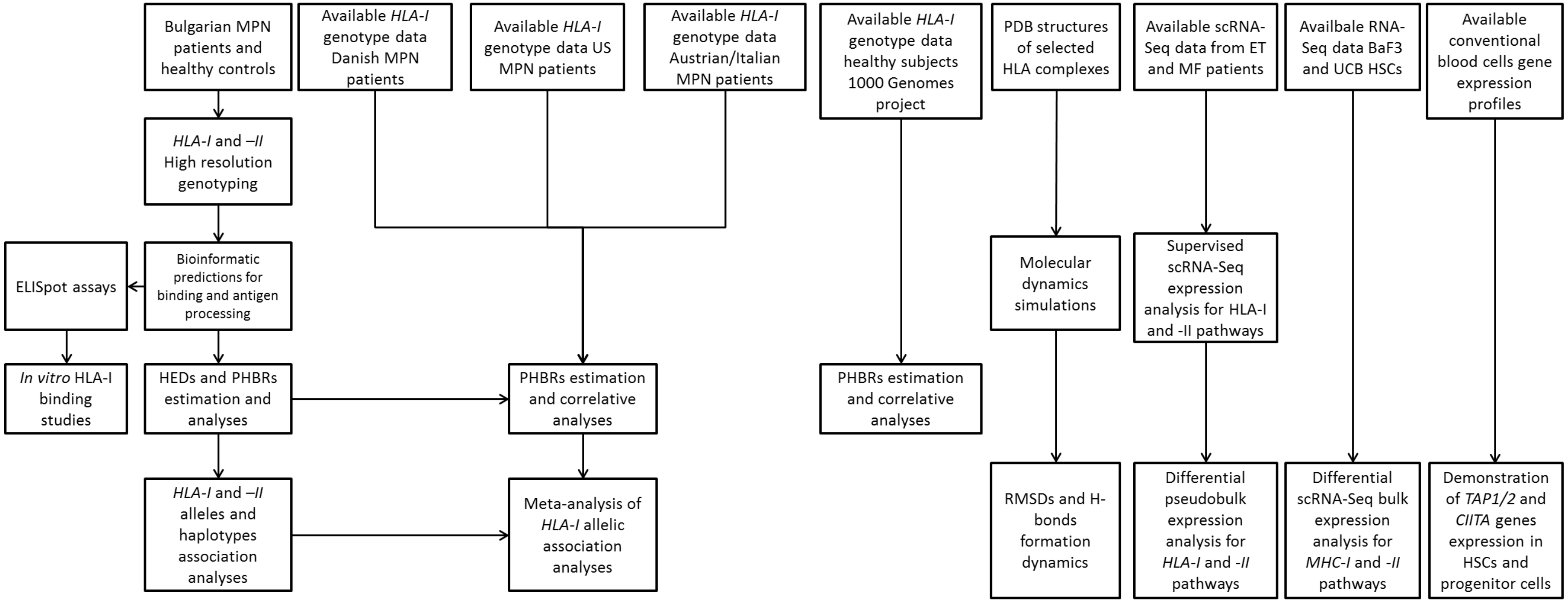

Supplementary Figure 2. Flowchart summarizing the used datasets and performed analyses as part of this study. Abbreviations are identical to those in the main text.

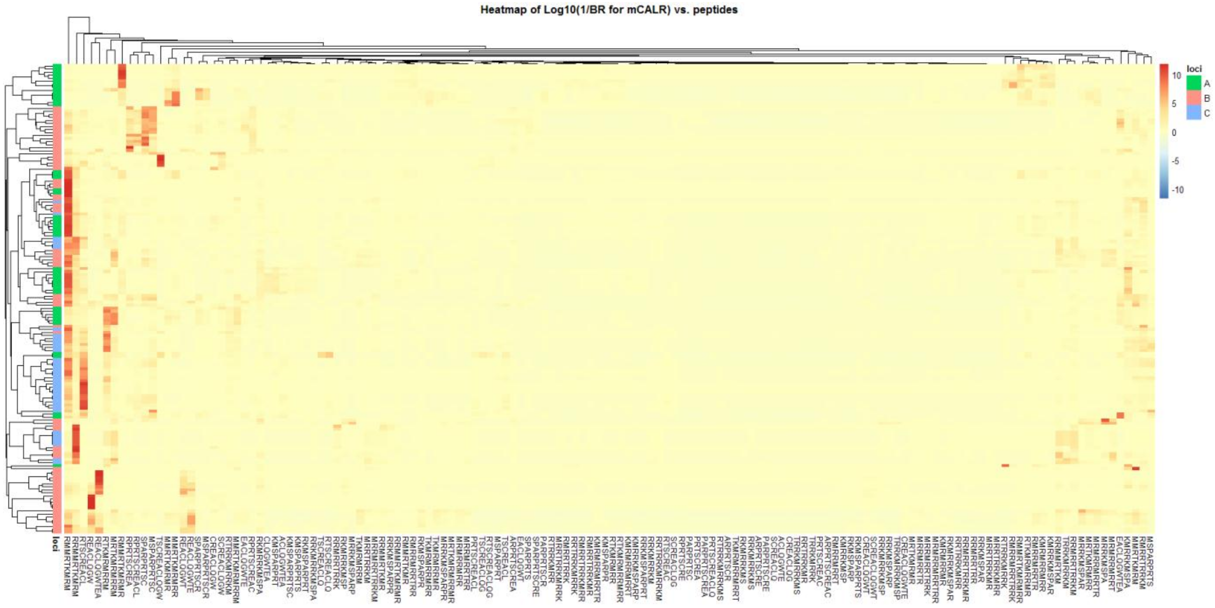

Supplementary Figure 3. Heatmap summarizing the NetMHCpan 4.1-predicted binding ranks (presented as log-transformed inverted values) for CALRmut-derived peptides across all identified *HLA-I* alleles in MPN patients and healthy controls. Each row represents a single *HLA-I* allele, and each column represents one of the 142 tested CALRmut-derived peptides. The higher the value of log10(1/BR) the stronger the predicted binding is (see legend for color coding of the range of values).

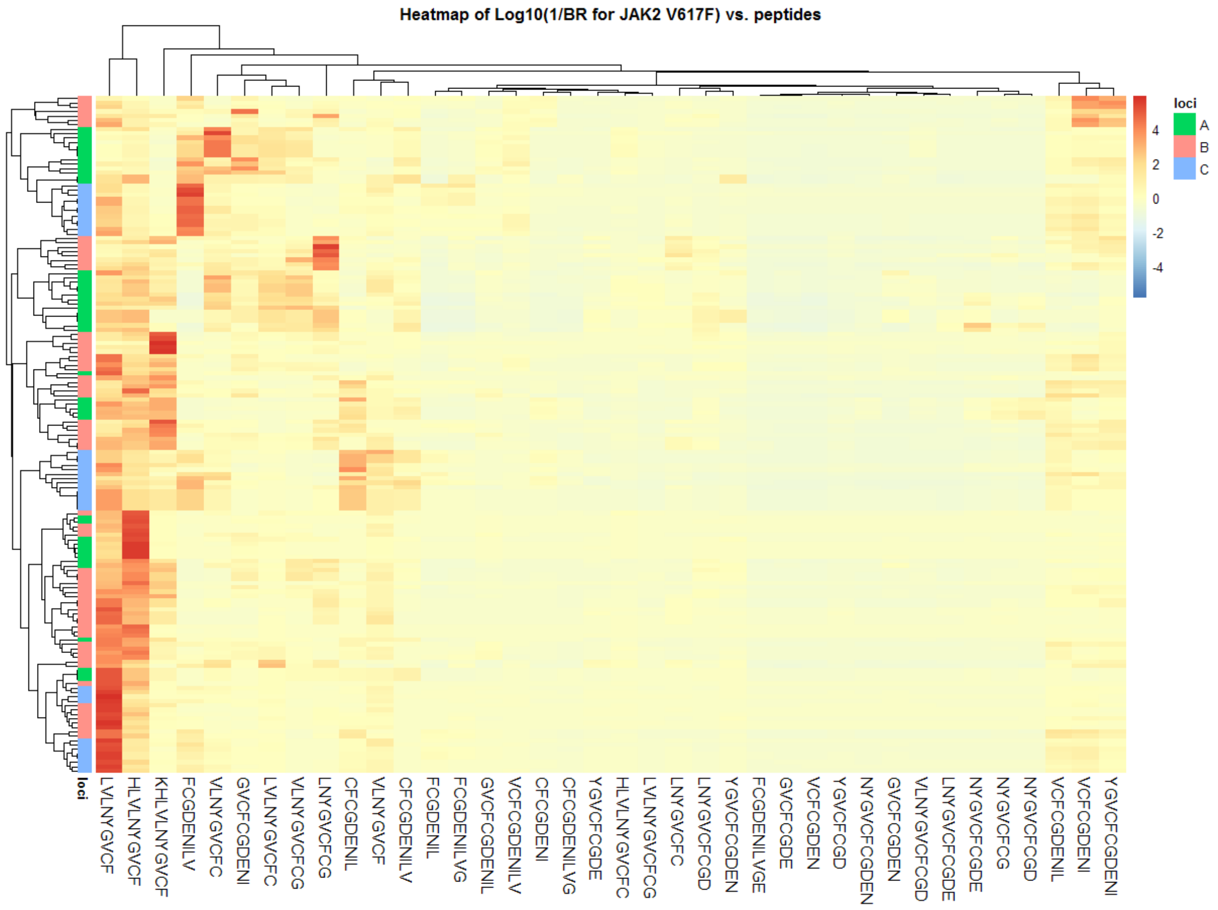

Supplementary Figure 4. Heatmap summarizing the NetMHCpan 4.1-predicted binding ranks (presented as log-transformed inverted values) for JAK2 V671F-derived peptides across all identified *HLA-I* alleles in MPN patients and healthy controls. Each row represents a single *HLA-I* allele, and each column represents one of the 38 tested JAK2 V671F-derived peptides. The higher the value of log10(1/BR) the stronger the predicted binding is (see legend for color coding of the range of values).

A

Subject	HLA-A	HLA-B	HLA-C
JAK2VF_001	A*24:02; A*30:02	B*18:01; B*35:01	C*04:01; C*07:01
JAK2VF_002	A*02:01; A*32:01	B*07:02; B*13:02	C*06:02; C*07:02
JAK2VF_003	A*02:01; A*02:01	B*41:01; B*44:27	C*07:04; C*17:01
JAK2VF_004	A*02:01; A*25:01	B*18:01; B*51:01	C*12:03; C*14:02
JAK2VF_005	A*02:05; A*11:01	B*38:01; B*50:01	C*04:01; C*06:02
JAK2VF_006	A*11:01; A*24:02	B*35:01; B*55:01	C*03:03; C*04:01

B

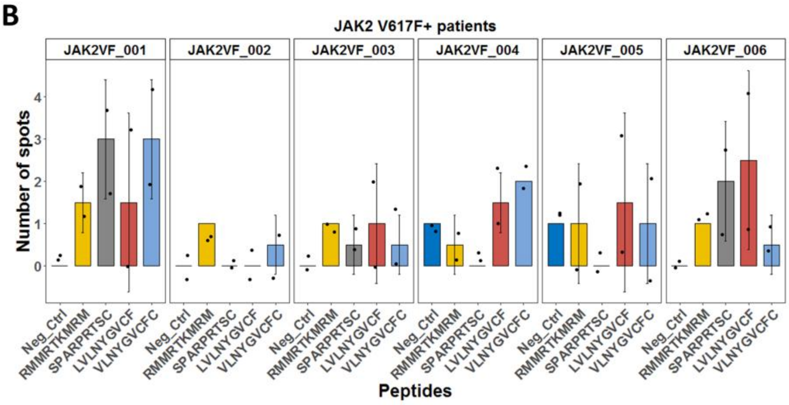

C

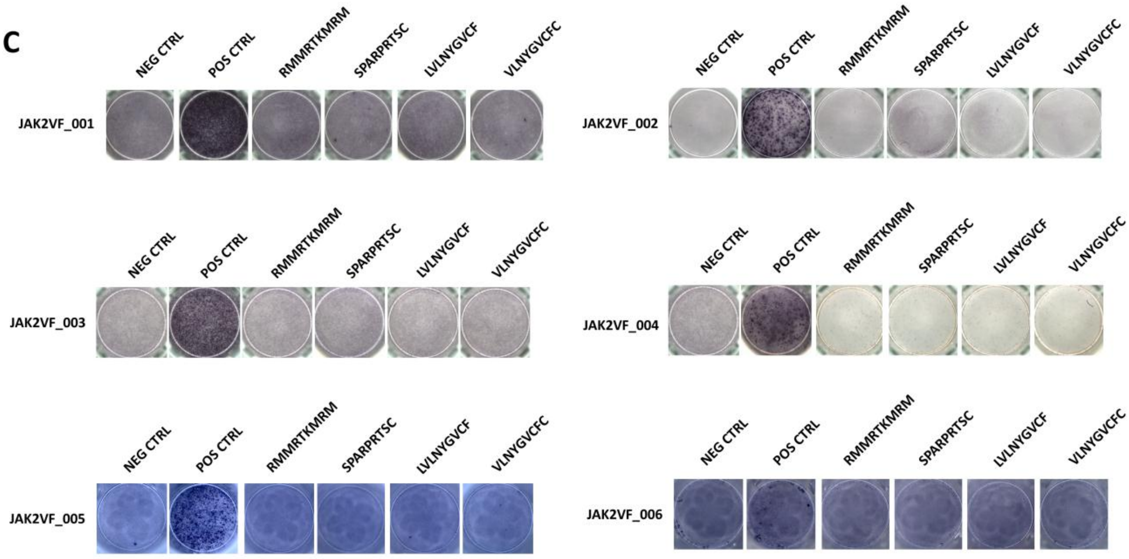

Supplementary Figure 5. ELISpot assays for detection of neoantigen-specific T cells in MPN patients with the JAK2 V617F mutation. VLNYGVCFC and LVLNYGVCF peptides are derived from JAK2 V617F sequence, and SPARPRTSC and RMMRTKMRM peptides are derived from the common neomorphic C-terminus of CALR. The negative control involved stimulation with the vehicle DMSO. A total of $2x10^5$ PBMCs were assayed per well. (A) *HLA-I* genotypes of patients; (B) Mean ± standard deviation (SD) of detected spots of two wells per sampled patient-peptide combination. Positive results were assumed if the mean per sampled combination was above the SD of the negative control for that patient and the absolute value of the mean was above 4 spots. No samples were considered positive. (C) Demonstration of the selected wells for the assays described in (B).

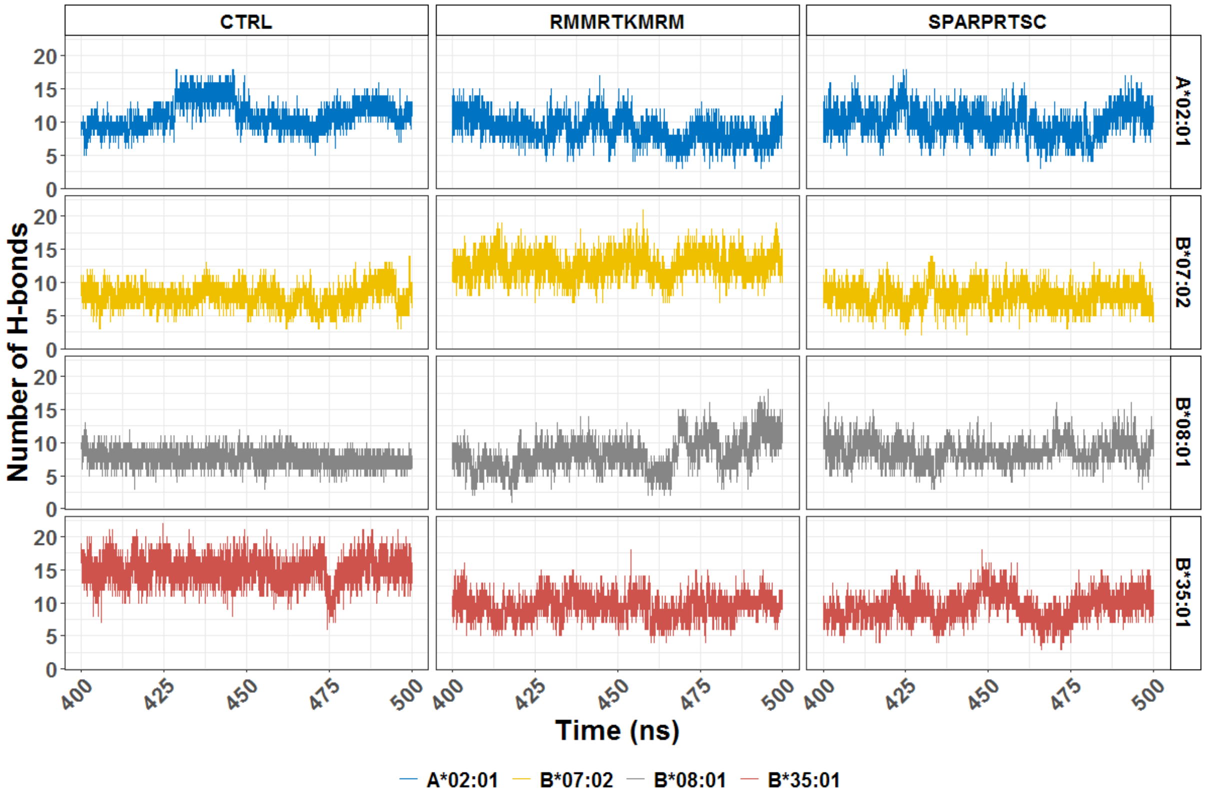

Supplementary Figure 6. Molecular dynamics simulations of two CALRmut-derived 9-mer peptides (RMMRTKMRM and SPARPRTSC) binding to HLA-A*02:01, HLA-B*07:02, HLA-B*08:01, and HLA-B*35:01. Dynamics of the number of H-bonds between the heavy chain and peptide were analyzed between 400 and 500 ns of simulation of HLA molecules conformers in complex with either a control peptide or one of the two CALRmut-derived peptides. Abbreviations: "CTRL"-control peptide; "ns"-nanoseconds.

A

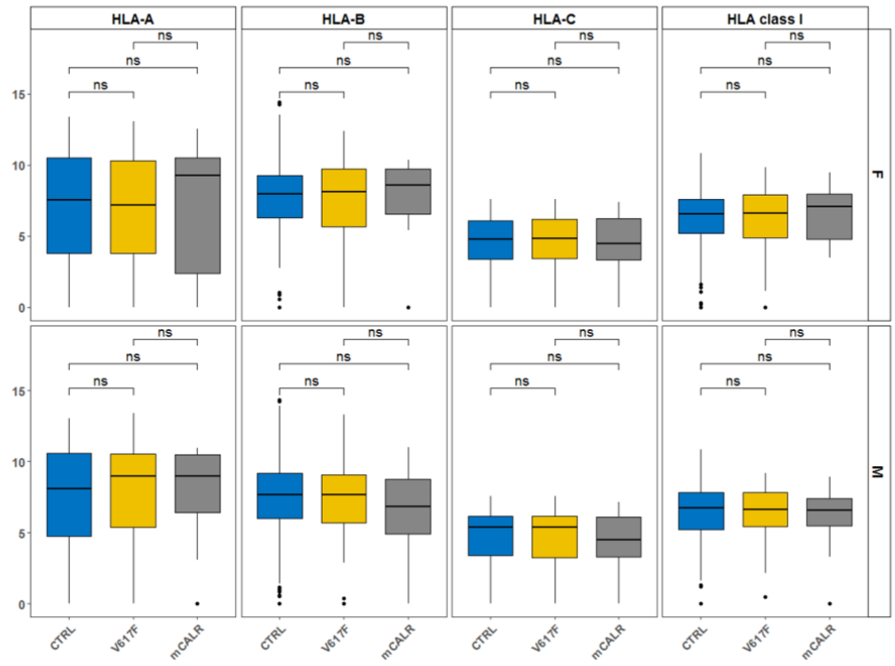

B

HED HLA-A locus

Variable		N	Estimate	p
**Group**	CTRL	1083	Reference	
	V617F	158	0.59 (-0.28, 1.45)	0.18
	mCALR	41	1.22 (-0.09, 2.54)	0.07
**Age**		1282	-0.01 (-0.03, 0.00)	0.10
**Sex**	F	700	Reference	
	M	582	0.47 (0.04, 0.89)	0.03

HED HLA-B locus

Variable		N	Estimate	p
**Group**	CTRL	1082	Reference	
	V617F	155	0.40 (-0.27, 1.07)	0.24
	mCALR	41	-0.03 (-1.05, 0.99)	0.96
**Age**		1278	-0.01 (-0.03, -0.00)	0.03
**Sex**	F	699	Reference	
	M	579	-0.26 (-0.59, 0.07)	0.12

HED HLA-C locus

Variable		N	Estimate	p
**Group**	CTRL	1073	Reference	
	V617F	148	0.18 (-0.30, 0.66)	0.5
	mCALR	41	0.02 (-0.69, 0.73)	1.0
**Age**		1262	-0.01 (-0.02, 0.00)	0.2
**Sex**	F	688	Reference	
	M	574	0.10 (-0.14, 0.33)	0.4

HED HLA class I

Variable		N	Estimate	p
**Group**	CTRL	1072	Reference	
	V617F	146	0.40 (-0.04, 0.84)	0.072
	mCALR	40	0.39 (-0.26, 1.04)	0.241
**Age**		1258	-0.01 (-0.02, -0.00)	0.008
**Sex**	F	688	Reference	
	M	570	0.11 (-0.10, 0.32)	0.302

Supplementary Figure 7. HED for the *HLA-I* locus. (A) Boxplot comparisons by locus and sex. P-values are from a two-sided Wilcoxon tests. (B) Forest plots summarizing the fitted linear models assessing the association of each HLA-I locus and all loci HED with the presence of CALR of JAK2 V617F mutation. Age and sex were included as covariates.

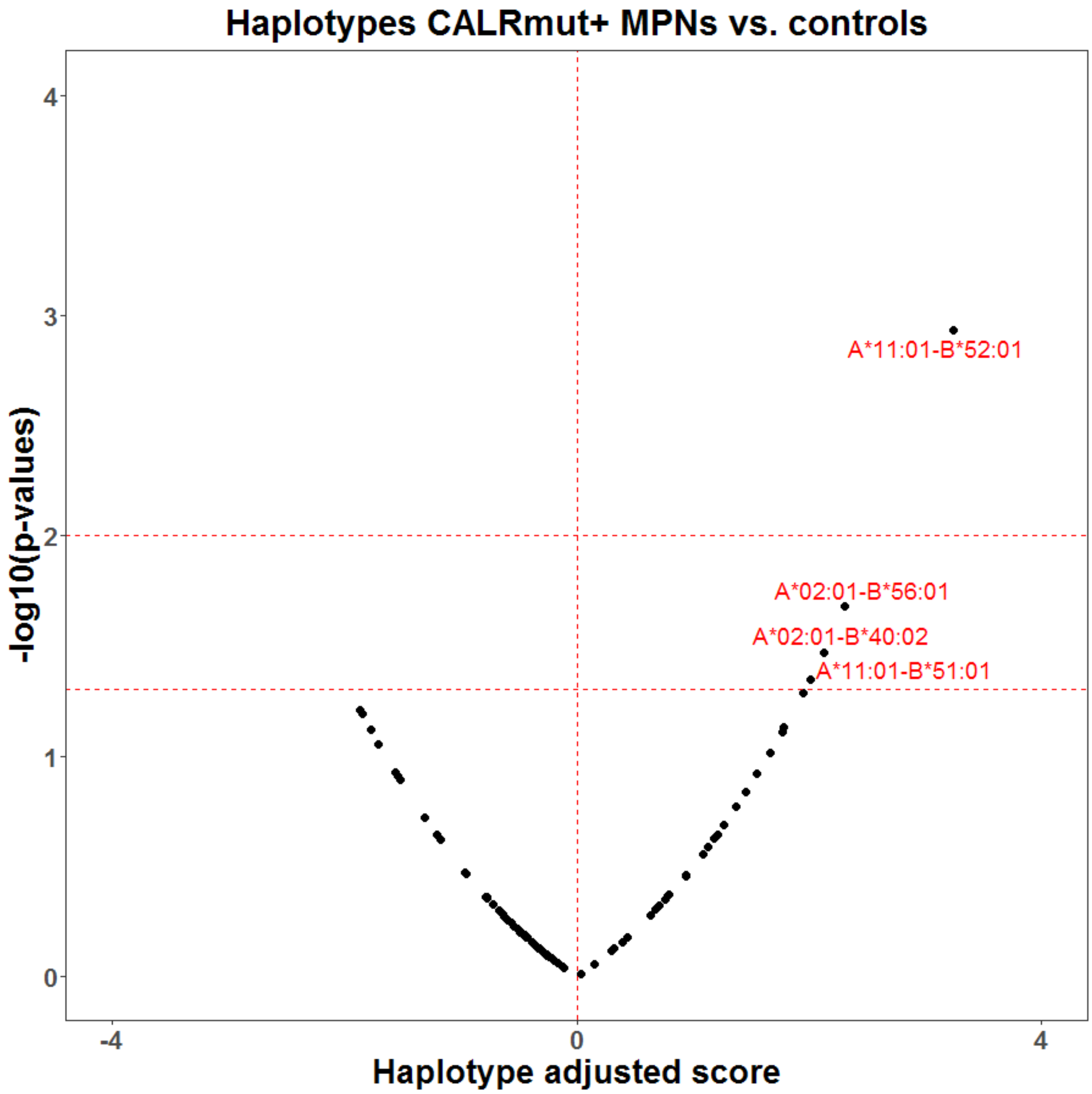

Supplementary Figure 8. Volcano plot of the association of *HLA-I A~B* bi-locus haplotypes with the presence of CALR mutation *versus* healthy controls.

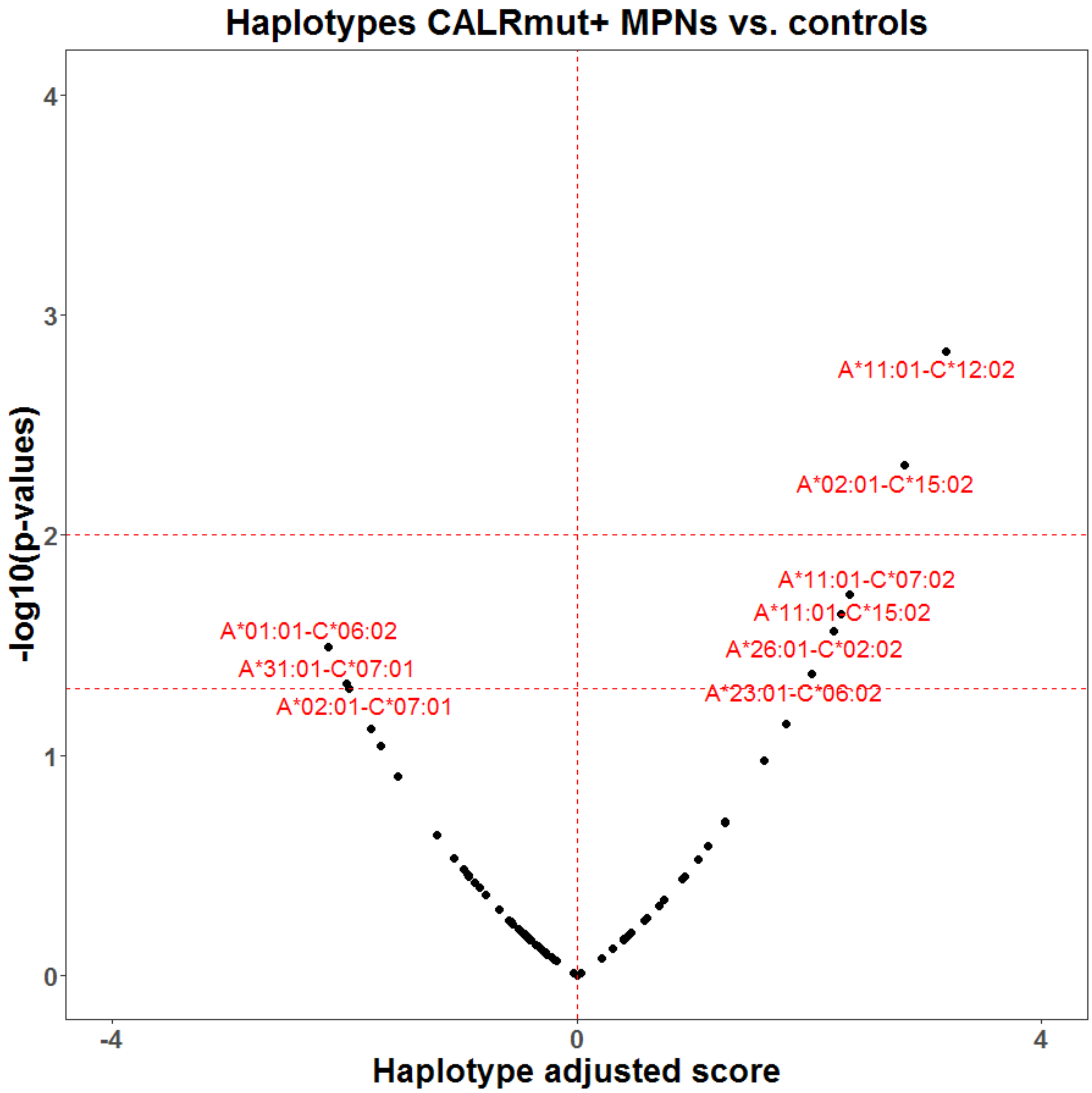

Supplementary Figure 9. Volcano plot of the association of *HLA-I A~C* bi-locus haplotypes with the presence of CALR mutation *versus* healthy controls.

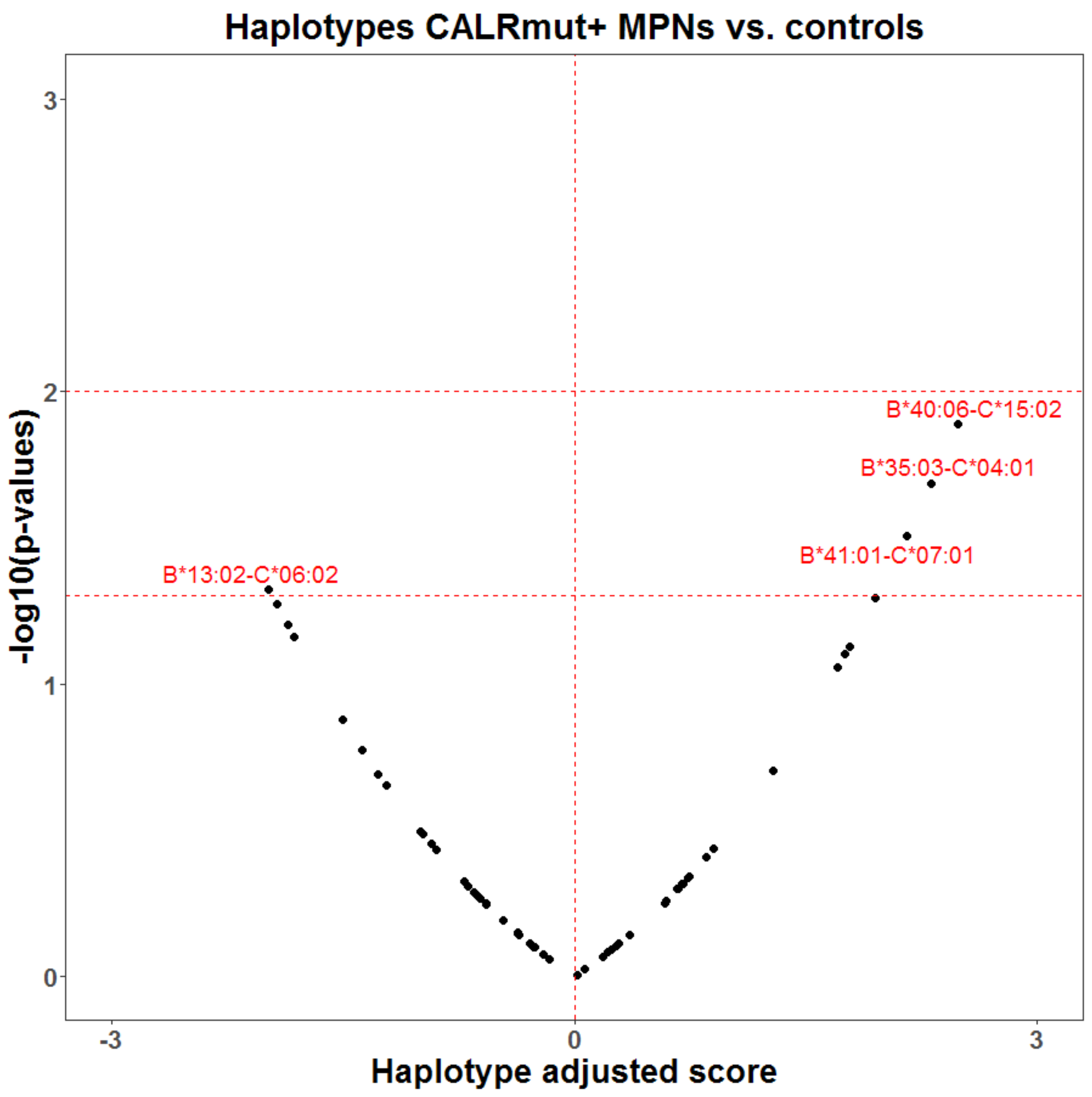

Supplementary Figure 10. Volcano plot of the association of *HLA-I B~C* bi-locus haplotypes with the presence of CALR mutation *versus* healthy controls.

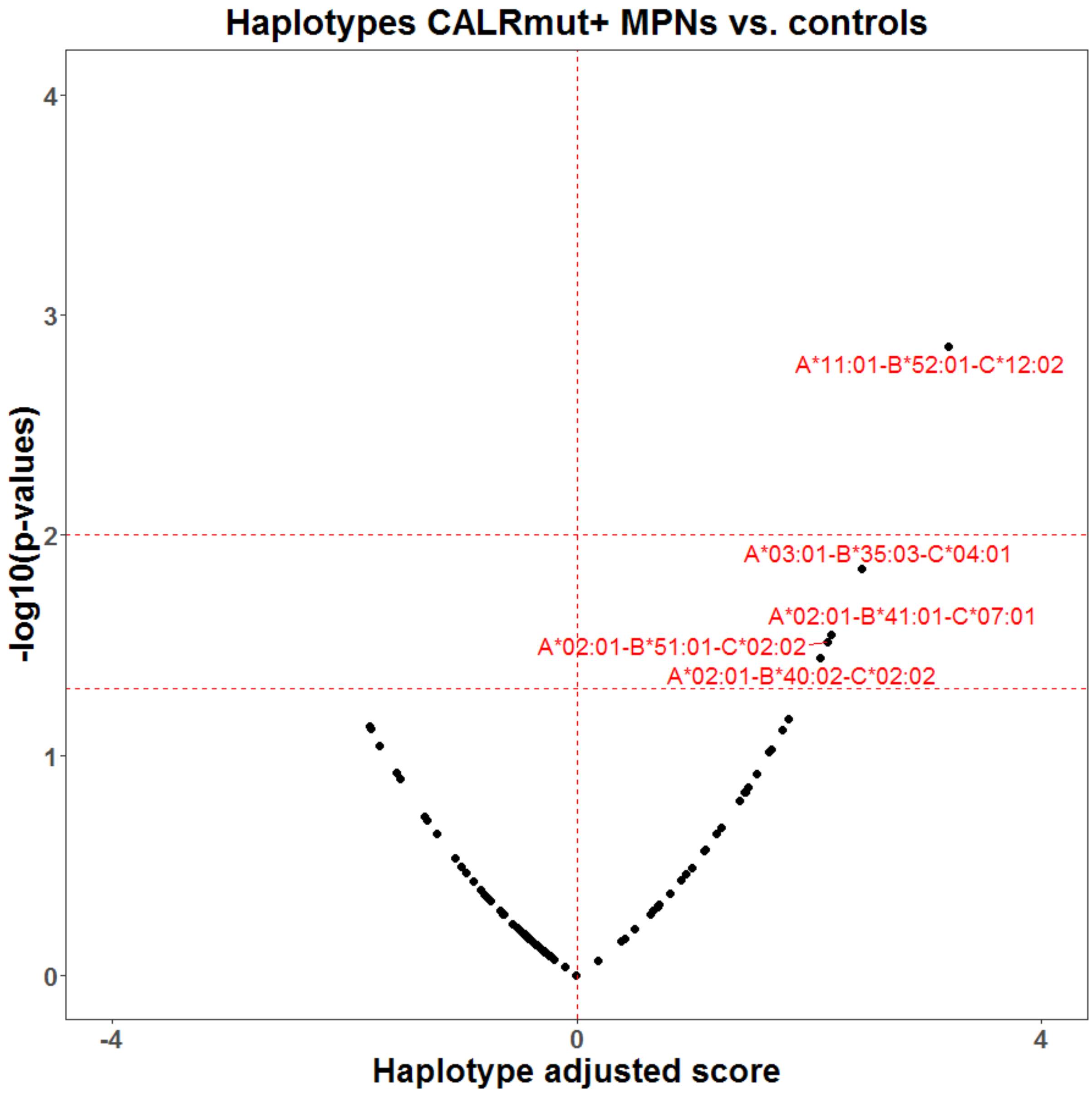

Supplementary Figure 11. Volcano plot of the association of *HLA-I A~B~C* tri-locus haplotypes with the presence of CALR mutation *versus* healthy controls.

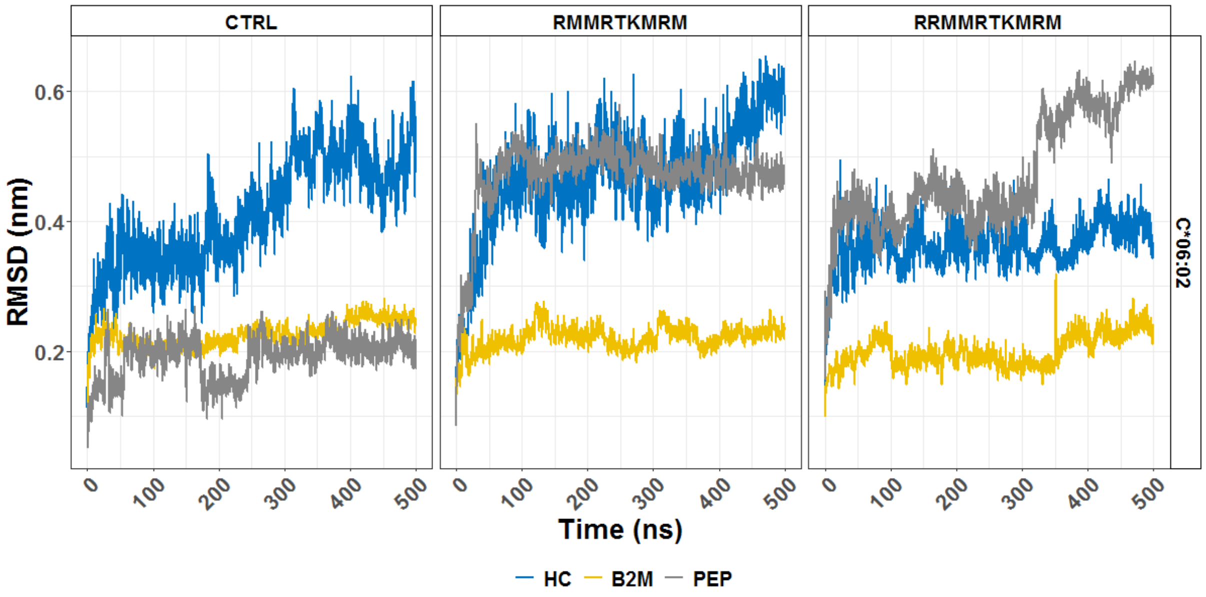

Supplementary Figure 12. Molecular dynamics simulations of two CALRmut-derived peptides – a nonamer (RMMRTKMRM) and a decamer (RRMMRTKMRM) - binding to HLA-C*06:02. Dynamics of the RMSDs of all atoms over 500 ns of simulation for HLA molecule conformers in complex with either a control peptide or one of the two CALRmut-derived peptides. Abbreviations: "RMSD" – root mean squared deviation; "PEP" – peptide; "nm" – nanometers; "ns" – nanoseconds.

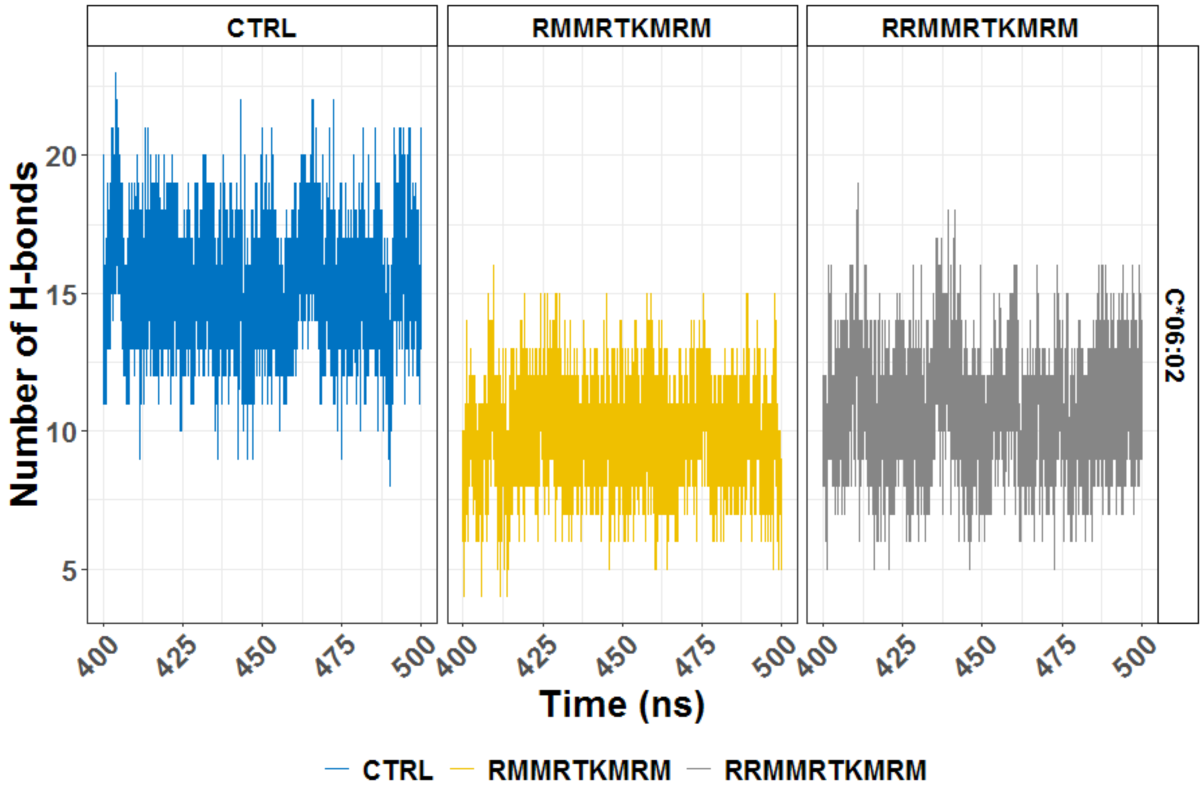

Supplementary Figure 13. Molecular dynamics simulations of two CALRmut-derived peptides – a nonamer (RMMRTKMRM) and a decamer (RRMMRTKMRM) – binding to HLA-C*06:02. Dynamics of the number of H-bonds between the heavy chain and peptide were analyzed between 400 and 500 ns of simulation for HLA molecule conformers in complex with either a control peptide or one of the two CALRmut-derived peptides. Abbreviations: "CTRL" – control peptide; "ns" – nanoseconds.

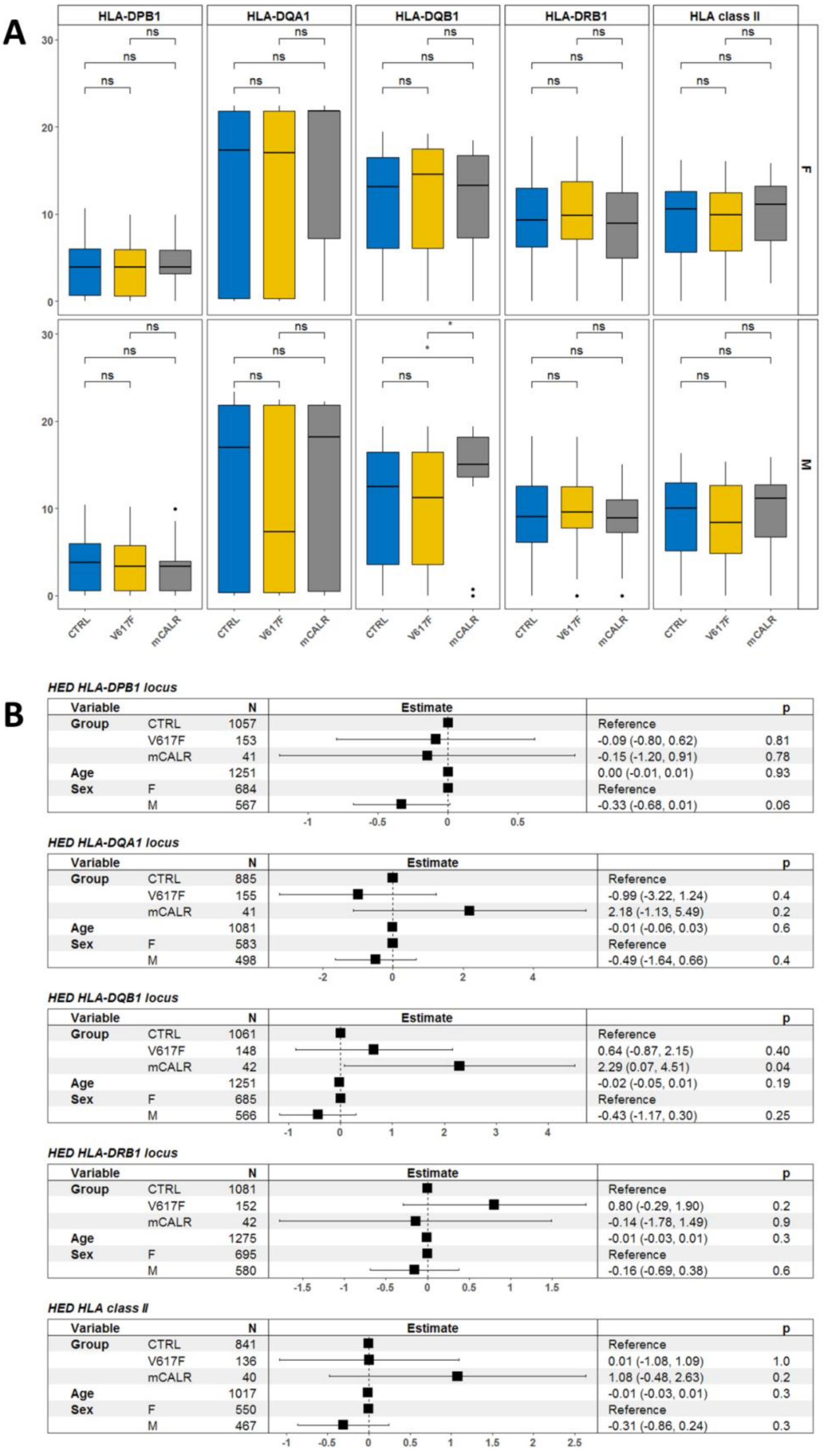

Supplementary Figure 14. HED for the *HLA-II* locus. (A) Boxplot comparisons by locus and sex. P-values are from two-sided Wilcoxon tests. (B) Forest plots summarizing the fitted linear models assessing the association of each HLA-II locus and all loci HED with presence of CALR or JAK2 V617F mutations. Age and sex were included as covariates.

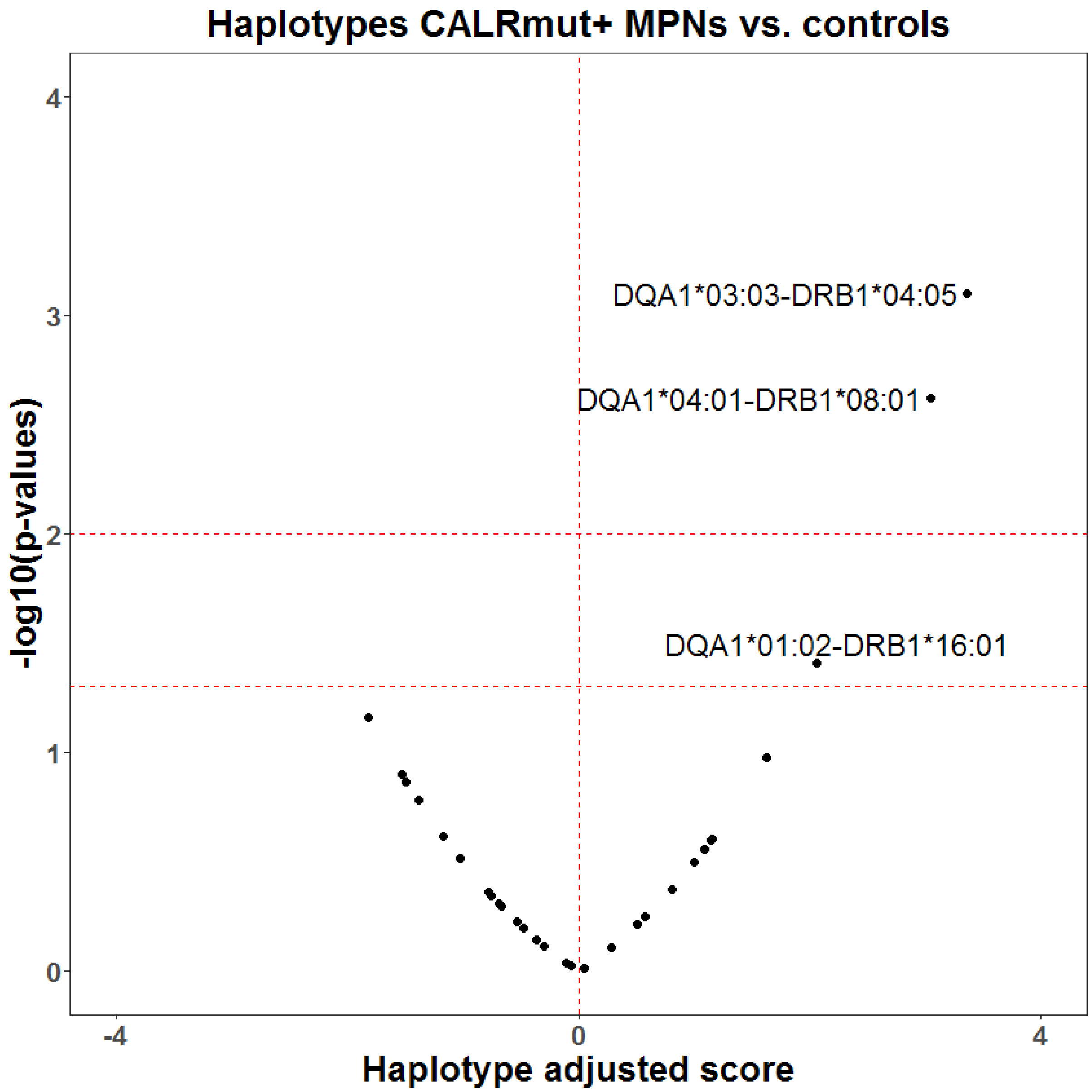

Supplementary Figure 15. Volcano plot of the association of *HLA-II DQA1~DRB1* bi-locus haplotypes with the presence of CALR mutation *versus* healthy controls.

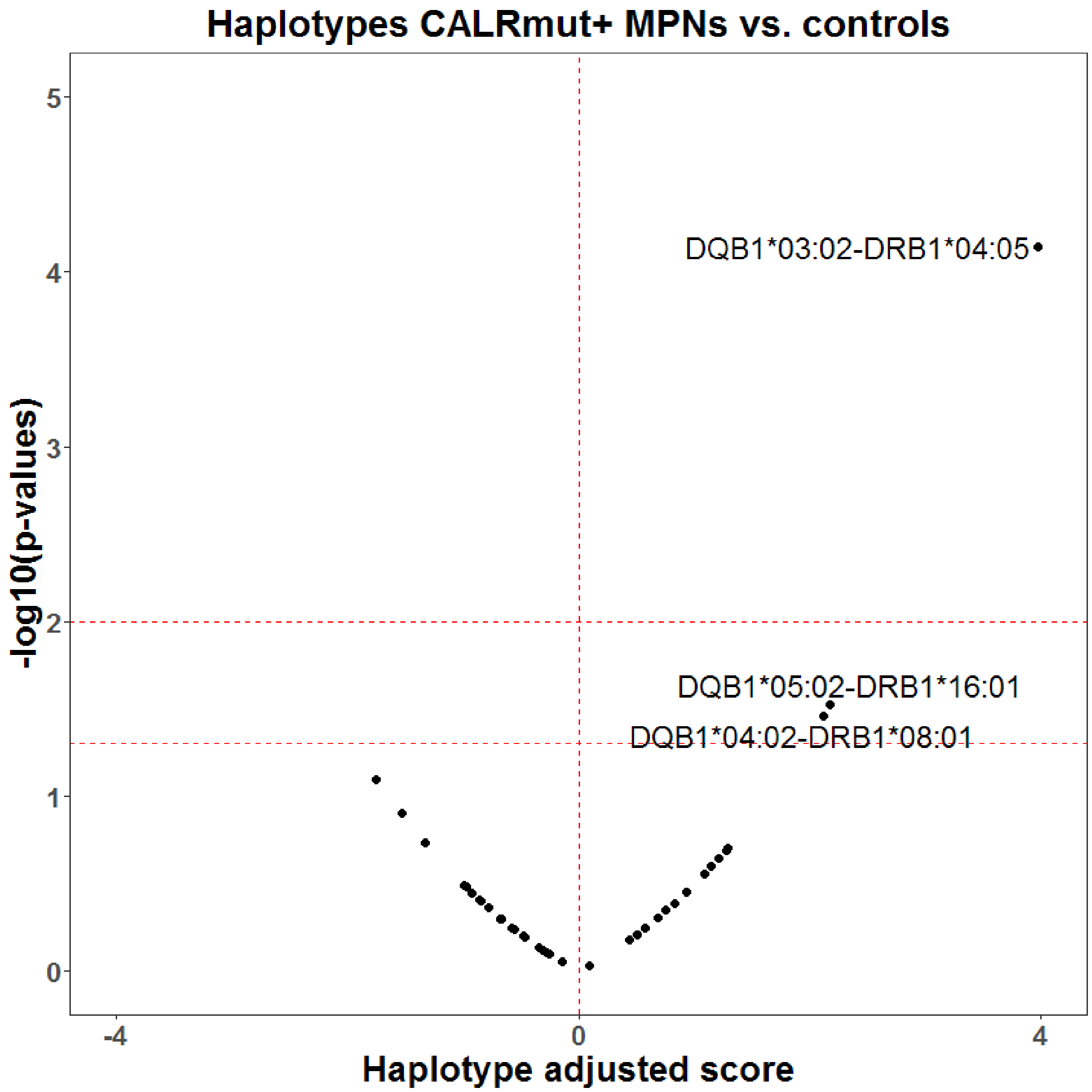

Supplementary Figure 16. Volcano plot of the association of *HLA-II DQB1~DRB1* bi-locus haplotypes with the presence of CALR mutation *versus* healthy controls.

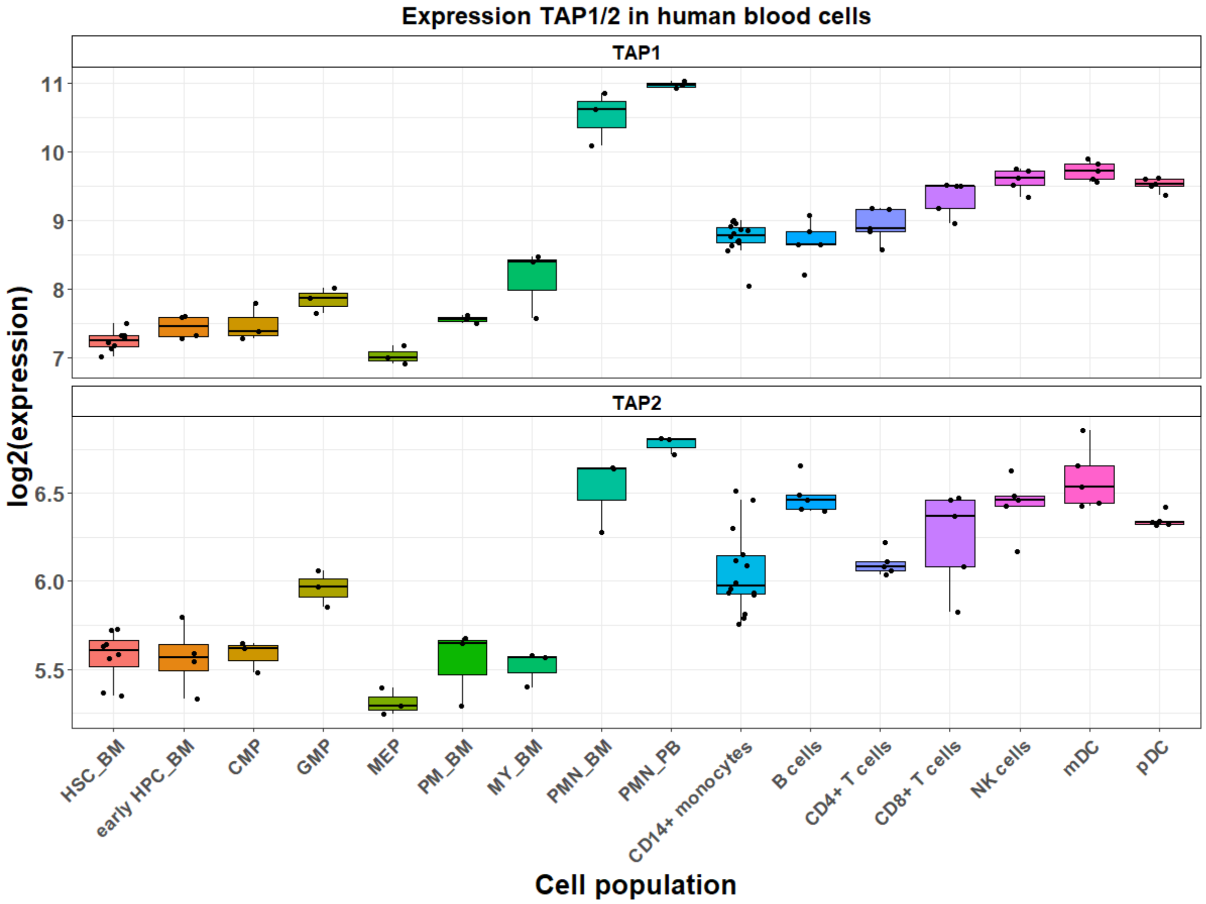

Supplementary Figure 17. Boxplot comparison of expression of the *TAP1* and *TAP2* genes using HemaExplorer data from sorted blood cells. Abbreviations: “HSC-BM” – Bone marrow hematopoietic stem cells; “early HPC_BM” – early bone marrow hematopoietic progenitors; “CMP” – common myeloid progenitors; “GMP” – granulocyte monocyte progenitors; “MEP” – megakaryocyte-erythroid progenitors; “PM_BM” – bone marrow promyelocytes; “MY_BM” – bone marrow myelocytes; “PMN_BM” – bone marrow polymorphonuclear cells; “PMN_PB” – peripheral blood polymorphonuclear cells.

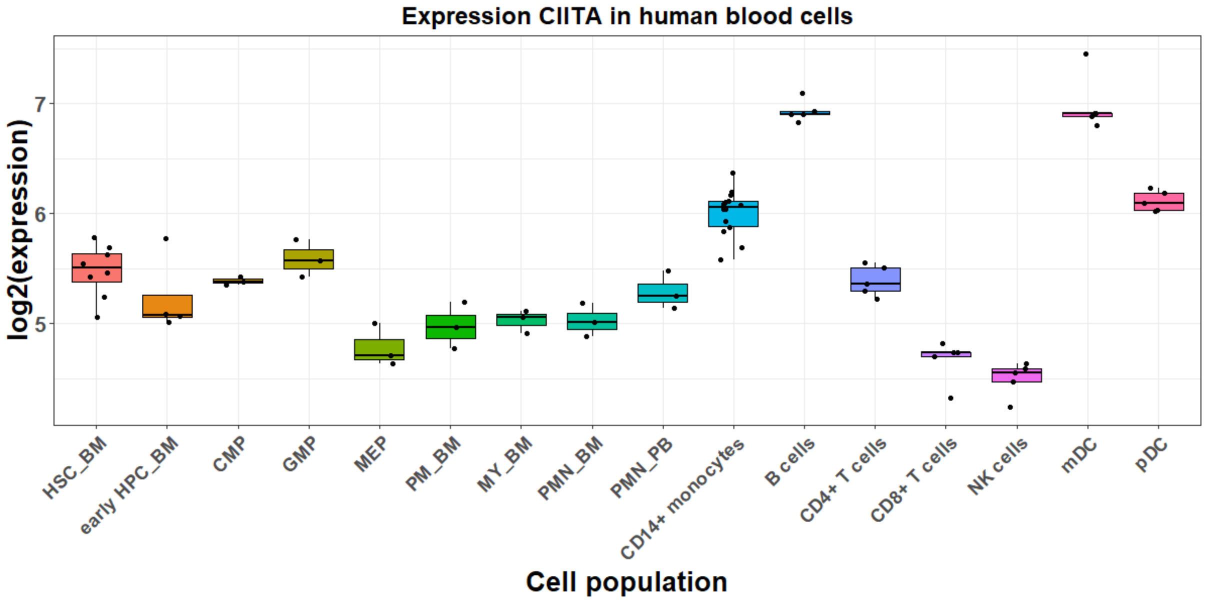

Supplementary Figure 18. Boxplot comparison of expression of the *CIITA* gene using HemaExplorer data from sorted blood cells. Abbreviations: "HSC-BM" – Bone marrow hematopoietic stem cells; "early HPC_BM" – early bone marrow hematopoietic progenitors; "CMP" – common myeloid progenitors; "GMP" – granulocyte monocyte progenitors; "MEP" – megakaryocyte-erythroid progenitors; "PM_BM" – bone marrow promyelocytes; "MY_BM" – bone marrow myelocytes; "PMN_BM" – bone marrow polymorphonuclear cells; "PMN_PB" – peripheral blood polymorphonuclear cells.

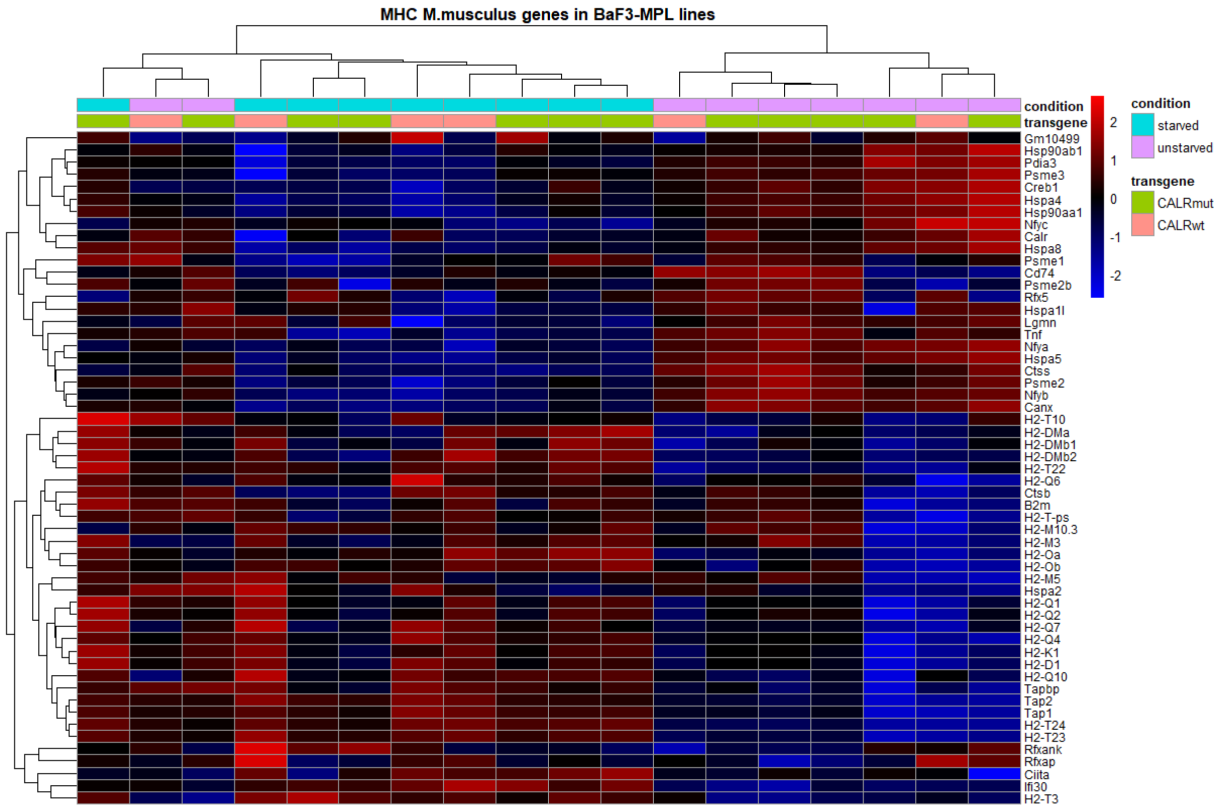

Supplementary Figure 19. Heatmap of *MHC-I* and *MHC–II* gene expression in mouse BaF3-MPL cells transduced with either human mutant or wild-type *CALR* genes under IL-3-starved or unstarved conditions.

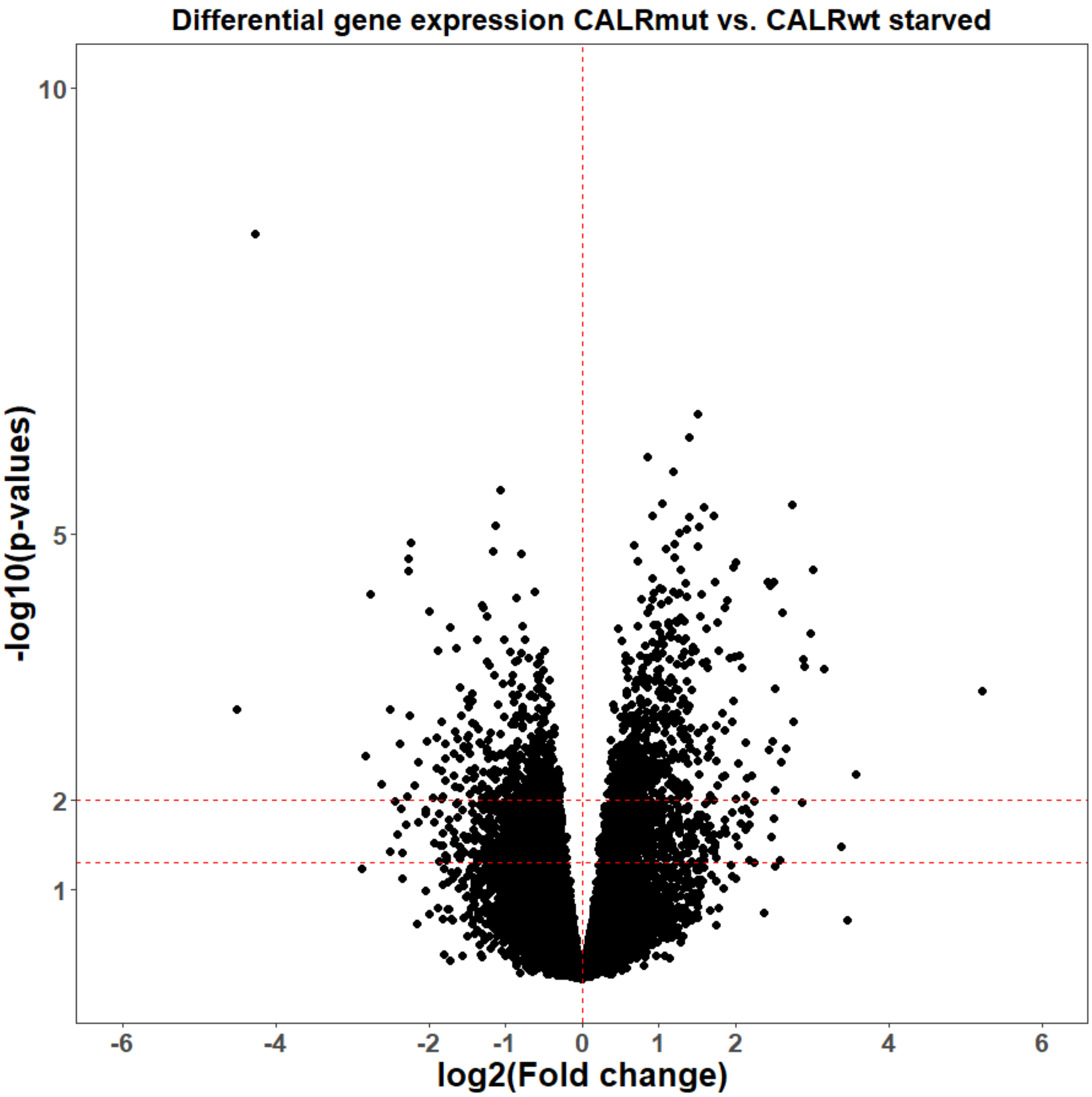

Supplementary Figure 20. Volcano plot of differentially expressed genes in mouse BaF3-MPL cells transduced with either human mutant or wild-type *CALR* genes under IL-3-starvation conditions. Note that there were no statistically significant overexpressed or underexpressed *MHC-I* or *MHC–II* genes.

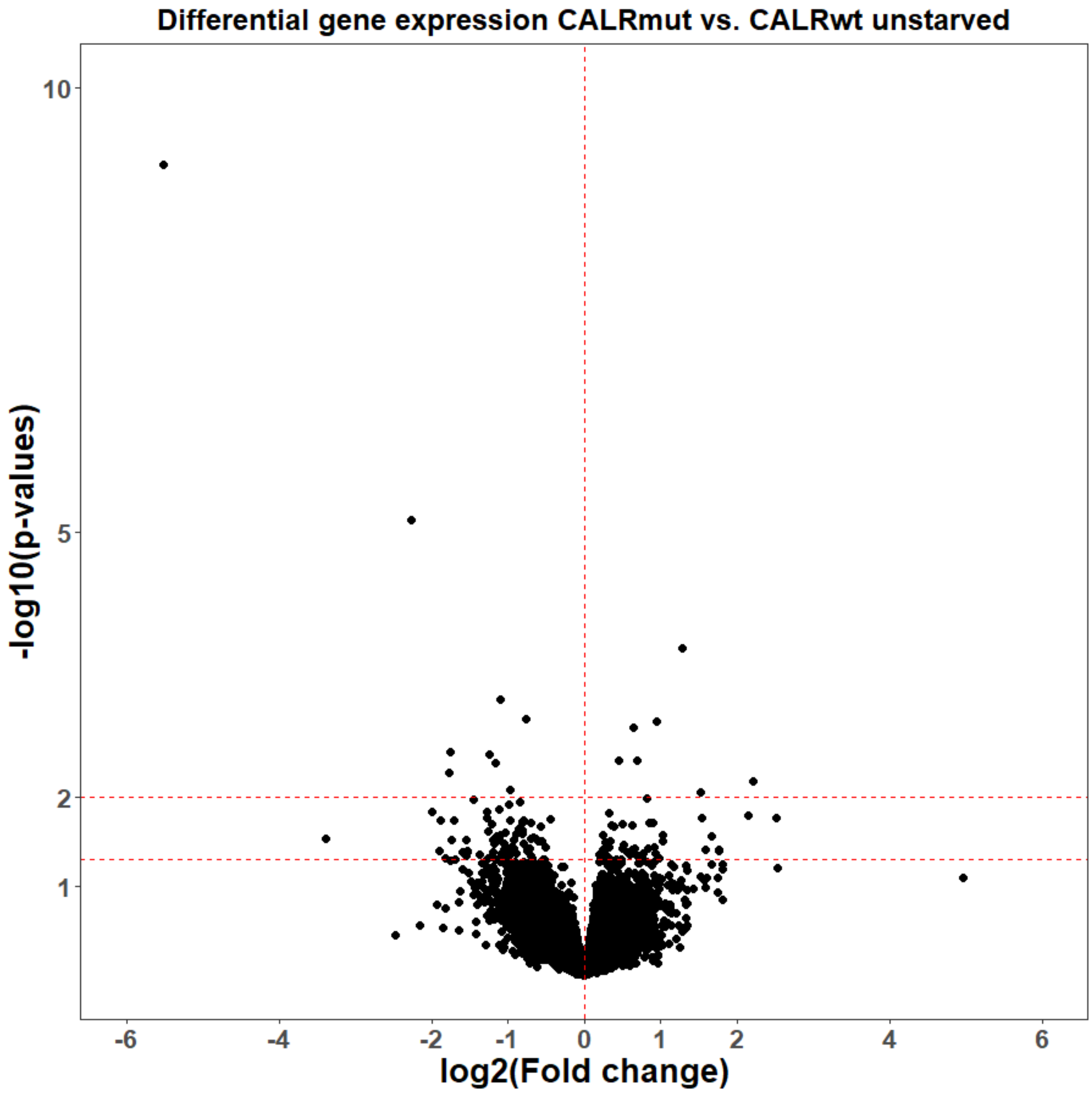

Supplementary Figure 21. Volcano plot of differentially expressed genes in mouse BaF3-MPL cells transduced with either human mutant or wild-type *CALR* genes under IL-3-unstarved conditions. Note that there were no statistically significantly overexpressed or underexpressed *MHC-I* or *MHC–II* genes.

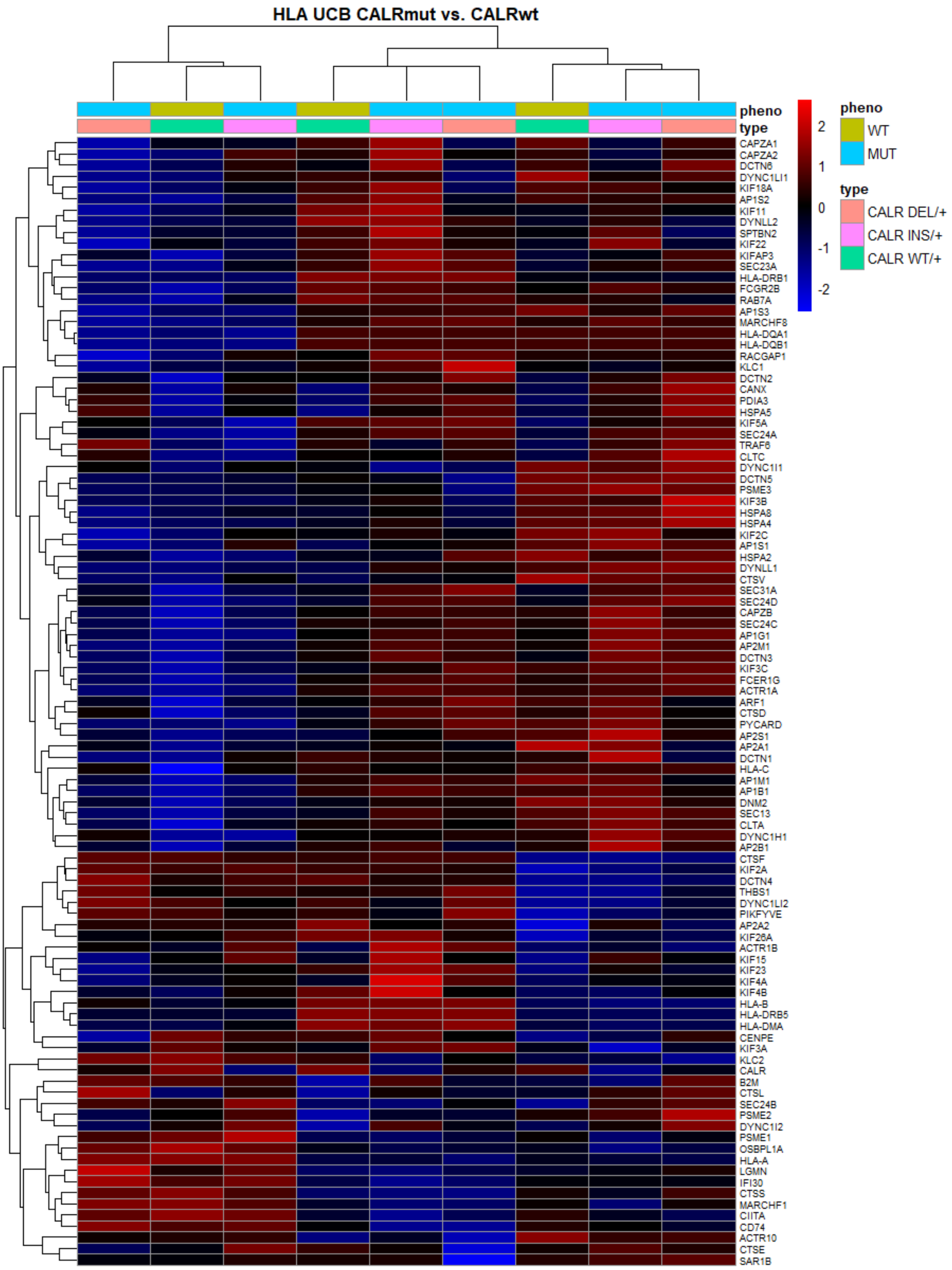

Supplementary Figure 22. Heatmap of *HLA-I* and *HLA-II* gene expression in human UCB cells with *CALR* mutation introduced by CRISPR/Cas9 editing compared to wild-type cells.

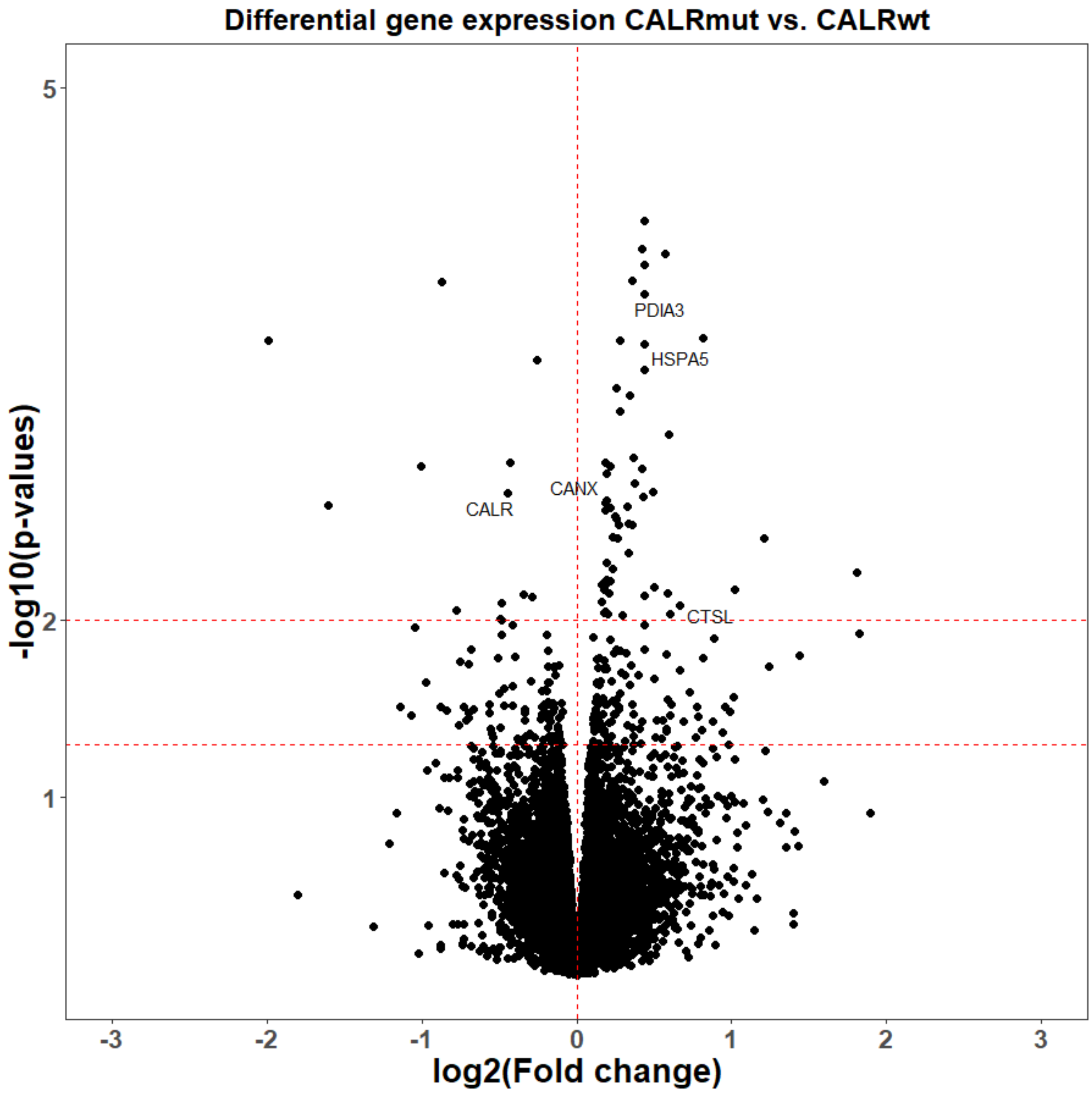

Supplementary Figure 23. Volcano plot of differentially expressed genes in human UCB cells with *CALR* mutation introduced by CRISPR/Cas9 editing compared to wild-type cells. Note that there were only a few genes that were statistically significant either as overexresed (*PDIA3*, *HSPA5*, *CANX* and *CTSL*) or underexpressed (*CALR*) in *HLA-I* or *HLA-II* pathway genes.

1.2 Supplementary Tables

Supplementary Table 1. Demographic and clinical characteristics of Bulgarian MPN patients and healthy controls included in the study. Designations: "*" P-value from the Kruskal-Wallis test comparing the age among the three groups; "†" P-value from the Chi-squared test comparing the gender distribution among the three groups; "‡"P-value from the Chi-squared test comparing the clinical diagnosis distribution between the two MPN groups.

Parameter	CALRmut+ MPN patients	JAK2 V617F+ MPN patients	Healthy controls	P-value
Age				<0.0001*
Median	66.5	70	32	
Range	(30-87)	(32-96)	(18-99)	
Gender (n)				0.602†
Male	22	69	492	
Female	20	89	591	
Total	42	158	1083	
Diagnosis (n)				
ET	16	52	0	<0.0001‡
PV	0	61	0	
PMF (post PV/ET MF)	15 (1)	33 (6)	0	
MPN, NOS	11	12	0	

Supplementary Table 2. SYFPEITHI-based prediction of binding affinity of nonamers from mutated CALR to four HLA alleles (A*02:01, B*07:02, B*08, and B*35:01). Peptides of special interest are in bold red font.

A*02:01			B*07:02			B*08			B*35:01		
Pos	Peptide	Score	Pos	Peptide	Score	Pos	Peptide	Score	Pos	Peptide	Score
23	KMSPARPRT	16	28	RPRTSCREA	19	17	RRKMRRKMS	19	25	**SPARPRTSC**	11
30	RTSCREACL	16	25	**SPARPRTSC**	16	5	RTKMRMRRM	18	28	RPRTSCREA	10
12	RMRRTRRKM	14	30	RTSCREACL	14	26	PARPRTSCR	18	30	RTSCREACL	10
19	KMRRKMSPA	14	23	KMSPARPRT	12	30	RTSCREACL	18	2	**RMMRTKMRM**	7
2	**RMMRTKMRM**	13	12	RMRRTRRKM	10	3	MMRTKMRMR	16	12	RMRRTRRKM	7
36	ACLQGWTEA	12	16	TRRKMRRKM	10	15	RTRRKMRRK	16	16	TRRKMRRKM	7
9	RMRRMRRTR	10	19	KMRRKMSPA	10	19	KMRRKMSPA	16	5	RTKMRMRRM	6
3	MMRTKMRMR	9	36	ACLQGWTEA	9	13	MRRTRRKMR	15	20	MRRKMSPAR	3
7	KMRMRRMRR	9	2	**RMMRTKMRM**	8	7	KMRMRRMRR	14	24	MSPARPRTS	3
5	RTKMRMRRM	7	34	REACLQGWT	8	10	MRRMRRTRR	14	31	TSCREACLQ	3
25	**SPARPRTSC**	7	5	RTKMRMRRM	7	28	RPRTSCREA	13	33	CREACLQGW	3
34	REACLQGWT	7	8	MRMRRMRRT	7	21	RRKMSPARP	10	4	MRTKMRMRR	2
16	TRRKMRRKM	6	22	RKMSPARPR	6	32	SCREACLQG	10	8	MRMRRMRRT	2
28	RPRTSCREA	6	20	MRRKMSPAR	5	1	RRMMRTKMR	9	9	RMRRMRRTR	2
8	MRMRRMRRT	5	7	KMRMRRMRR	4	8	MRMRRMRRT	8	3	MMRTKMRMR	1
15	RTRRKMRRK	5	9	RMRRMRRTR	4	11	RRMRRTRRK	8	6	TKMRMRRMR	1
26	PARPRTSCR	5	10	MRRMRRTRR	4	14	RRTRRKMRR	8	10	MRRMRRTRR	1
32	SCREACLQG	5	26	PARPRTSCR	4	18	RKMRRKMSP	8	11	RRMRRTRRK	1
33	CREACLQGW	4	32	SCREACLQG	4	24	MSPARPRTS	8	13	MRRTRRKMR	1
11	RRMRRTRRK	3	1	RRMMRTKMR	3	25	**SPARPRTSC**	8	14	RRTRRKMRR	1
18	RKMRRKMSP	3	11	RRMRRTRRK	3	20	MRRKMSPAR	7	15	RTRRKMRRK	1
35	EACLQGWTE	3	13	MRRTRRKMR	3	35	EACLQGWTE	7	18	RKMRRKMSP	1
1	RRMMRTKMR	2	15	RTRRKMRRK	3	9	RMRRMRRTR	6	19	KMRRKMSPA	1
20	MRRKMSPAR	2	3	MMRTKMRMR	2	12	RMRRTRRKM	6	22	RKMSPARPR	1
22	RKMSPARPR	2	4	MRTKMRMRR	2	16	TRRKMRRKM	6	27	ARPRTSCRE	1
24	MSPARPRTS	2	6	TKMRMRRMR	2	29	PRTSCREAC	1	29	PRTSCREAC	1
27	ARPRTSCRE	2	17	RRKMRRKMS	2	36	ACLQGWTEA	1	32	SCREACLQG	1
6	TKMRMRRMR	1	18	RKMRRKMSP	2	2	**RMMRTKMRM**	0	34	REACLQGWT	1
21	RRKMSPARP	1	21	RRKMSPARP	2	4	MRTKMRMRR	0	35	EACLQGWTE	1

10	MRRMRRTRR	0	27	ARPRTSCRE	2	6	TKMRMRRMR	0	36	ACLQGWTEA	1
31	TSCREACLQ	0	29	PRTSCREAC	2	22	RKMSPARPR	0	1	RRMMRTKMR	0
4	MRTKMRMRR	-1	35	EACLQGWTE	2	23	KMSPARPRT	0	7	KMRMRRMRR	0
13	MRRTRRKMR	-1	14	RRTRRKMRR	1	27	ARPRTSCRE	0	17	RRKMRRKMS	0
14	RRTRRKMRR	-1	24	MSPARPRTS	1	31	TSCREACLQ	0	21	RRKMSPARP	0
17	RRKMRRKMS	-2	33	CREACLQGW	1	33	CREACLQGW	0	23	KMSPARPRT	0
29	PRTSCREAC	-3	31	TSCREACLQ	0	34	REACLQGWT	0	26	PARPRTSCR	0

Supplementary Table 3. NetMHCchop 3.0-based prediction using the C-term method for proteasomal cleavage sites in the mutated CALR sequence. The initial 44-mer peptide sequence was: **RRMMRTKMRMRRMRRTRRKMRRKMSPARPRTSCREACLQGWTEA**. Predicted cleavage sites are those with score above 0.5 (the default value for the server). The "S" symbol in the Cleavage column denotes a predicted cleavage site after the marked amino acid position. The dot "." symbol in the Cleavage column denotes that the score for that amino acid is below 0.5 and therefore is not considered a predicted cleavage site after the marked amino acid position. The score is an estimate from a neural network prediction algorithm and ranges between 0 and 1.

Position	***Amino acid***	***Cleavage***	***Score***
1	R	.	0.092199
2	R	.	0.120418
3	M	.	0.465463
4	M	.	0.305689
5	R	.	0.04105
6	T	.	0.037997
7	K	.	0.10345
8	M	.	0.261841
9	R	.	0.417158
10	M	S	0.587093
11	R	.	0.056343
12	R	.	0.45055
13	M	.	0.293013
14	R	.	0.033864
15	R	.	0.072223
16	T	.	0.038081
17	R	.	0.074184
18	R	S	0.619269
19	K	S	0.91302
20	M	S	0.698259

21	R	.	0.310845
22	R	S	0.922966
23	K	.	0.291502
24	M	S	0.619963
25	S	.	0.032519
26	P	.	0.041507
27	A	.	0.062686
28	R	.	0.49505
29	P	.	0.16319
30	R	.	0.160104
31	T	.	0.137282
32	S	.	0.045343
33	C	.	0.203269
34	R	S	0.932168
35	E	.	0.209642
36	A	S	0.884404
37	C	.	0.023011
38	L	S	0.91756
39	Q	.	0.025001
40	G	.	0.063924
41	W	S	0.514234
42	T	.	0.047934
43	E	.	0.041729
44	A	S	0.524592

Supplementary Table 4. NetMHCchop 3.0-based prediction using the 20S method for proteasomal cleavage sites in the mutated CALR sequence. The initial 44-mer peptide sequence was: **RRMMRTKMRMRRMRRTRRKMRRKMSPARPRTSCREACLQGWTEA**. Predicted cleavage sites are those with score above 0.5 (the default value for the server). The "S" symbol in the Cleavage column denotes predicted cleavage site after the marked amino acid position. The dot "." symbol in the Cleavage column denotes that the score for that amino acid is below 0.5 and therefore is not considered a predicted cleavage site after the marked amino acid position. The score is an estimate from a neural network prediction algorithm and ranges between 0 and 1.

Position	*Amino acid*	*Cleavage*	*Score*
1	R	S	0.669046
2	R	S	0.878428
3	M	S	0.765988
4	M	S	0.952529
5	R	S	0.867658
6	T	S	0.663214
7	K	S	0.575957
8	M	S	0.942465
9	R	S	0.966675
10	M	S	0.937329
11	R	S	0.977862
12	R	S	0.964452
13	M	S	0.933107
14	R	S	0.978274
15	R	S	0.953169
16	T	.	0.272409
17	R	S	0.862355
18	R	S	0.947008
19	K	.	0.433875

20	M	S	0.770525
21	R	S	0.898162
22	R	S	0.926951
23	K	.	0.177161
24	M	S	0.736338
25	S	.	0.491171
26	P	.	0.076288
27	A	.	0.043607
28	R	.	0.416496
29	P	.	0.243236
30	R	.	0.082987
31	T	.	0.280884
32	S	S	0.513631
33	C	S	0.556596
34	R	S	0.832585
35	E	S	0.940442
36	A	S	0.553137
37	C	.	0.291034
38	L	.	0.361263
39	Q	S	0.758991
40	G	S	0.541802
41	W	S	0.592685
42	T	.	0.042123
43	E	S	0.943239
44	A	S	0.573911

Supplementary Table 5. TAPPred server-based prediction of TAP binding offinity of nonamer peptides from the mutated CALR C-terminus. The submitted protein sequence was the 44-mer peptide: **RRMMRTKMRMRRMRRTRRKMRRKMSPARPRTSCREACLQGWTEA**. Prediction was based on the TAPPred SVM method. Peptides of special interest are bolded and shown in red.

Peptide Rank	*Start Position*	*Sequence*	*Score*	*Predicted Affinity*
1	14	RRTRRKMRR	10.041	High
2	10	MRRMRRTRR	8.967	High
3	9	**RMRRMRRTR**	8.857	High
4	4	MRTKMRMRR	8.791	High
5	7	KMRMRRMRR	8.77	High
6	11	RRMRRTRRK	8.545	High
7	16	TRRKMRRKM	8.446	High
8	18	RKMRRKMSP	8.409	High
9	6	TKMRMRRMR	7.855	High
10	8	MRMRRMRRT	7.636	High
11	2	RMMRTKMRM	7.636	High
12	20	MRRKMSPAR	7.302	High
13	1	RRMMRTKMR	7.232	High
14	15	RTRRKMRRK	7.144	High
15	5	RTKMRMRRM	6.916	High
16	13	MRRTRRKMR	6.757	High
17	22	RKMSPARPR	6.669	High
18	12	RMRRTRRKM	6.421	High
19	26	PARPRTSCR	6.35	High
20	21	RRKMSPARP	5.738	Intermidate
21	30	RTSCREACL	5.232	Intermidate
22	17	RRKMRRKMS	4.783	Intermidate
23	19	KMRRKMSPA	4.765	Intermidate
24	3	MMRTKMRMR	4.136	Intermidate
25	23	KMSPARPRT	3.54	Intermidate
26	27	ARPRTSCRE	3.499	Intermidate
27	36	ACLQGWTEA	3.423	Intermidate
28	34	REACLQGWT	3.09	Intermidate

29	33	CREACLQGW	2.901	Low or undetectable
30	25	**SPARPRTSC**	2.877	Low or undetectable
31	31	TSCREACLQ	2.219	Low or undetectable
32	32	SCREACLQG	2.01	Low or undetectable
33	35	EACLQGWTE	0.921	Low or undetectable
34	28	RPRTSCREA	0.772	Low or undetectable
35	29	PRTSCREAC	0.689	Low or undetectable
36	24	MSPARPRTS	-0.392	Low or undetectable

Supplementary Table 6. TAPPred server-based prediction of TAP binding affinity of nonamer peptides from the mutated CALR C-terminus. The submitted protein sequence was the 44-mer peptide: **RRMMRTKMRMRRMRRTRRKMRRKMSPARPRTSCREACLQGWTEA**. Prediction was based on the TAPPred cascade SVM method. Peptides of special interest are bolded and shown in red.

Peptide Rank	*Start Position*	*Sequence*	*Score*	*Predicted Affinity*
1	32	SCREACLQG	8.475	High
2	31	TSCREACLQ	7.98	High
3	33	CREACLQGW	6.747	High
4	34	REACLQGWT	6.151	High
5	25	**SPARPRTSC**	6.029	High
6	35	EACLQGWTE	5.71	Intermediate
7	29	PRTSCREAC	5.307	Intermediate
8	36	ACLQGWTEA	5.281	Intermediate
9	23	KMSPARPRT	5.121	Intermediate
10	28	RPRTSCREA	5.037	Intermediate
11	27	ARPRTSCRE	4.928	Intermediate
12	3	MMRTKMRMR	4.306	Intermediate
13	19	KMRRKMSPA	3.962	Intermediate
14	17	RRKMRRKMS	3.955	Intermediate
15	30	RTSCREACL	3.876	Intermediate
16	21	RRKMSPARP	3.849	Intermediate
17	26	PARPRTSCR	3.842	Intermediate
18	12	RMRRTRRKM	3.842	Intermediate
19	22	RKMSPARPR	3.841	Intermediate
20	13	MRRTRRKMR	3.841	Intermediate
21	5	RTKMRMRRM	3.841	Intermediate
22	15	RTRRKMRRK	3.841	Intermediate
23	1	RRMMRTKMR	3.841	Intermediate
24	20	MRRKMSPAR	3.841	Intermediate
25	8	MRMRRMRRT	3.841	Intermediate
26	2	RMMRTKMRM	3.841	Intermediate
27	6	TKMRMRRMR	3.841	Intermediate
28	16	TRRKMRRKM	3.841	Intermediate

29	18	RKMRRKMSP	3.841	Intermediate
30	11	RRMRRTRRK	3.841	Intermediate
31	4	MRTKMRMRR	3.841	Intermediate
32	7	KMRMRRMRR	3.841	Intermediate
33	9	**RMRRMRRTR**	3.841	Intermediate
34	10	MRRMRRTRR	3.841	Intermediate
35	14	RRTRRKMRR	3.841	Intermediate
36	24	MSPARPRTS	-0.933	Low or undetectable

Supplementary Table 7. NetMHCstab 1.0 predictions of binding for all CALR-mutated peptides for three HLA molecules: HLA-A_02:01, HLA-B_07:02, and HLA-B*35:01. Rows of special interest are in bold red font.

		HLA-A*02:01					HLA-B*07:02					HLA-B*35:01				
Pos	Peptide	Pred	Thalf	1-log50k	nM	Comb	Pred	Thalf	1-log50k	nM	Comb	Pred	Thalf	1-log50k	nM	Comb
1	RRMMRTKM	0.0232	0.3685	0.055	27575.71	0.0502	0.0519	0.4686	0.202	5651.03	0.1795	0.0106	0.3049	0.061	25702.97	0.0534
2	RMMRTKMR	0.0491	0.4601	0.068	23957.42	0.0652	0.0119	0.3127	0.056	27278.95	0.0494	0.0065	0.2755	0.038	33144.32	0.0333
3	MMRTKMRM	0.0429	0.4402	0.145	10414.04	0.1297	0.0911	0.5786	0.31	1746.99	0.2772	0.0521	0.4691	0.232	4084.68	0.205
4	MRTKMRMR	0.0052	0.2632	0.029	36534.22	0.0254	0.0044	0.2555	0.036	33869.37	0.0313	0.0084	0.2898	0.023	38984.65	0.0208
5	RTKMRMRR	0.0052	0.2634	0.034	34610.27	0.0297	0.0087	0.2922	0.051	28951.56	0.0447	0.0052	0.2633	0.018	41151.77	0.0161
6	TKMRMRRM	0.0152	0.3313	0.038	33144.32	0.0346	0.0297	0.3942	0.13	12315.54	0.115	0.0296	0.394	0.085	19932.31	0.0767
7	KMRMRRMR	0.0082	0.2887	0.04	32434.8	0.0352	0.0252	0.3767	0.079	21269.21	0.0709	0.0048	0.2596	0.026	37944.28	0.0228
8	MRMRRMRR	0.0062	0.2731	0.039	32787.64	0.0341	0.0053	0.2645	0.041	32085.75	0.0356	0.0088	0.2926	0.027	37535.94	0.0243
9	RMRRMRRT	0.0187	0.3486	0.054	27725.29	0.0487	0.0557	0.4801	0.19	6434.51	0.1699	0.0049	0.2608	0.022	39196.13	0.0194
10	MRRMRRTR	0.0042	0.2531	0.027	37333.42	0.0236	0.0125	0.3164	0.057	26985.39	0.0503	0.006	0.2706	0.028	36732.4	0.0247
11	RRMRRTRR	0.0065	0.2757	0.034	34610.27	0.0299	0.0108	0.306	0.048	29906.73	0.0424	0.0048	0.2594	0.018	41151.77	0.016
12	RMRRTRRK	0.0073	0.2819	0.032	35559.24	0.0283	0.0264	0.3813	0.102	16583.46	0.0907	0.0044	0.2554	0.024	38565.12	0.0211
13	MRRTRRKM	0.0051	0.2626	0.03	36141.06	0.0263	0.0701	0.5215	0.175	7527.49	0.1593	0.0112	0.3085	0.054	27875.69	0.0476
14	RRTRRKMR	0.0049	0.261	0.025	38357.05	0.022	0.0069	0.2785	0.035	34423.54	0.0308	0.0047	0.2588	0.013	43439.36	0.0118
15	RTRRKMRR	0.0051	0.263	0.029	36337.11	0.0254	0.0201	0.3548	0.062	25426.37	0.0557	0.0048	0.2596	0.017	41375	0.0152
16	TRRKMRRK	0.0039	0.2502	0.022	39408.75	0.0193	0.007	0.2795	0.044	31060.99	0.0385	0.0047	0.259	0.016	42051.98	0.0143
17	RRKMRRKM	0.0056	0.2677	0.028	36732.4	0.0246	0.0479	0.4563	0.132	12051.9	0.1194	0.0057	0.2682	0.026	37944.28	0.023
18	RKMRRKMS	0.0103	0.3031	0.032	35559.24	0.0287	0.0226	0.3657	0.057	26985.39	0.0518	0.0059	0.2703	0.015	42280.1	0.0136

19	KMRRKMSP	0.0253	0.377	0.048	29745.37	0.0446	0.041	0.4341	0.085	20040.43	0.0784	0.0053	0.2649	0.02	40270.83	0.0178
20	MRRKMSPA	0.0092	0.2957	0.049	29584.89	0.043	0.024	0.3719	0.161	8758.62	0.1405	0.0099	0.3004	0.059	26407.71	0.0516
21	RRKMSPAR	0.0047	0.2586	0.028	37132	0.0245	0.008	0.2874	0.044	31229.48	0.0386	0.0058	0.2694	0.022	39408.75	0.0196
22	RKMSPARP	0.0187	0.3484	0.03	35946.07	0.0283	0.0143	0.3266	0.043	31398.89	0.0387	0.0094	0.2968	0.018	40929.75	0.0167
23	KMSPARPR	0.0267	0.3827	0.084	20258.44	0.0754	0.0192	0.3508	0.055	27575.71	0.0496	0.0074	0.2826	0.056	27426.93	0.0487
24	MSPARPRT	0.0084	0.2903	0.046	30232.07	0.0404	0.005	0.2613	0.046	30232.07	0.0398	0.017	0.3402	0.04	32434.8	0.0365
25	SPARPRTS	0.0054	0.2659	0.029	36337.11	0.0255	0.4427	1.7013	0.336	1318.61	0.352	0.0665	0.5115	0.113	14722.66	0.106
26	PARPRTSC	0.0057	0.2683	0.025	38150.11	0.0221	0.1759	0.7977	0.085	19824.77	0.0986	0.0287	0.3902	0.025	38357.05	0.0255
27	ARPRTSCR	0.0062	0.2726	0.024	38565.12	0.0213	0.0045	0.2565	0.029	36534.22	0.0253	0.005	0.2616	0.014	42740.04	0.0126
28	RPRTSCRE	0.0049	0.2609	0.024	38565.12	0.0211	0.4157	1.5794	0.269	2707.68	0.291	0.0485	0.458	0.046	30232.07	0.0464
29	PRTSCREA	0.0079	0.2861	0.024	38565.12	0.0216	0.0051	0.2625	0.032	35559.24	0.028	0.0068	0.2781	0.015	42740.04	0.0138
30	RTSCREAC	0.0222	0.3639	0.048	29745.37	0.0441	0.0906	0.5773	0.099	17223.5	0.0977	0.0074	0.2824	0.029	36337.11	0.0258
31	TSCREACL	0.0155	0.3325	0.042	31569.21	0.038	0.0132	0.3205	0.04	32434.8	0.036	0.0622	0.4991	0.063	25289.19	0.0629
32	SCREACLQ	0.0071	0.2799	0.027	37333.42	0.024	0.0202	0.3552	0.031	35752.13	0.0294	0.023	0.3677	0.034	34610.27	0.0324
33	CREACLQG	0.0055	0.2662	0.031	35752.13	0.0272	0.0054	0.2652	0.029	36534.22	0.0255	0.0093	0.2967	0.023	38984.65	0.021
34	REACLQGW	0.0066	0.2758	0.043	31229.48	0.0375	0.0116	0.3109	0.043	31398.89	0.0383	0.0097	0.2993	0.033	35176.57	0.0295
35	EACLQGWT	0.0046	0.2579	0.03	36141.06	0.0262	0.0053	0.2645	0.035	34423.54	0.0305	0.0658	0.5095	0.04	32610.74	0.0439
36	ACLQGWTE	0.0203	0.3559	0.036	34053.09	0.0337	0.0068	0.2779	0.028	36732.4	0.0248	0.0111	0.3083	0.035	34237.82	0.0314
37	CLQGWTEA	0.1685	0.7785	0.345	1196.26	0.3185	0.0112	0.3084	0.054	27725.29	0.0476	0.006	0.2708	0.046	30396.07	0.04
38	RRMMRTKMR	0.0073	0.2818	0.041	32085.75	0.0359	0.0083	0.2891	0.06	25982.58	0.0522	0.0056	0.2672	0.02	40053.56	0.0178
39	RMMRTKMRM	0.4224	1.6088	0.328	1437.83	0.3422	0.0706	0.523	0.351	1121.07	0.3089	0.0193	0.351	0.152	9602.34	0.1321
40	MMRTKMRMR	0.0074	0.2827	0.051	28951.56	0.0445	0.0116	0.3108	0.073	22818.9	0.0638	0.0074	0.2822	0.037	33504.88	0.0326

41	MRTKMRMRR	0.005	0.2618	0.032	35559.24	0.028	0.0043	0.2541	0.052	28485.48	0.0448	0.009	0.2946	0.022	39196.13	0.0201
42	RTKMRMRRM	0.0093	0.2962	0.05	29266.51	0.0439	0.0611	0.4958	0.307	1804.62	0.2701	0.0077	0.2846	0.039	32787.64	0.0343
43	TKMRMRRMR	0.0056	0.2674	0.03	35946.07	0.0263	0.0073	0.2818	0.051	28640	0.0444	0.0068	0.2776	0.019	40708.92	0.0172
44	KMRMRRMRR	0.0089	0.2938	0.045	30726.73	0.0396	0.0227	0.3664	0.1	17038.15	0.0884	0.0048	0.2598	0.025	38150.11	0.022
45	MRMRRMRRT	0.0123	0.3154	0.056	27278.95	0.0495	0.0071	0.2803	0.091	18679.43	0.0784	0.0075	0.2831	0.025	38357.05	0.0224
**46**	**RMRRMRRTR**	**0.0067**	**0.2766**	**0.036**	**33869.37**	**0.0316**	**0.0619**	**0.4984**	**0.137**	**11417.23**	**0.1257**	**0.0045**	**0.2569**	**0.03**	**36141.06**	**0.0262**
47	MRRMRRTRR	0.0039	0.2495	0.028	36931.66	0.0244	0.0085	0.291	0.07	23571.74	0.0608	0.0056	0.2671	0.021	39622.53	0.0187
48	RRMRRTRRK	0.0062	0.2728	0.036	33869.37	0.0315	0.0093	0.2962	0.078	21500.59	0.0677	0.0044	0.2552	0.018	41375	0.016
49	RMRRTRRKM	0.0133	0.3211	0.048	29906.73	0.0428	0.3777	1.4239	0.576	98.79	0.5463	0.0058	0.2688	0.059	26265.23	0.051
50	MRRTRRKMR	0.004	0.2513	0.026	37535.94	0.0227	0.0099	0.3003	0.058	26694.99	0.0508	0.0055	0.2669	0.018	41151.77	0.0161
51	RRTRRKMRR	0.0051	0.2626	0.029	36732.4	0.0254	0.0052	0.2637	0.047	30068.96	0.0407	0.0048	0.2601	0.012	43675	0.0109
52	RTRRKMRRK	0.0045	0.2567	0.03	35946.07	0.0262	0.0192	0.3507	0.11	15290.89	0.0964	0.0041	0.2518	0.013	43204.99	0.0117
53	TRRKMRRKM	0.0049	0.2602	0.027	37333.42	0.0237	0.0538	0.4742	0.14	10992.95	0.1271	0.007	0.2791	0.035	34237.82	0.0308
54	RRKMRRKMS	0.0048	0.2592	0.027	37333.42	0.0237	0.0117	0.3116	0.064	25017.04	0.0562	0.0045	0.256	0.011	44150.12	0.01
55	RKMRRKMSP	0.016	0.3353	0.035	34237.82	0.0322	0.024	0.3715	0.081	20813.9	0.0724	0.0059	0.2705	0.015	42280.1	0.0136
56	KMRRKMSPA	0.069	0.5185	0.247	3435.38	0.2203	0.134	0.6896	0.391	727.23	0.3524	0.0054	0.2657	0.039	32610.74	0.034
57	MRRKMSPAR	0.0049	0.2609	0.035	34423.54	0.0305	0.0115	0.3107	0.08	21154.46	0.0697	0.0092	0.2956	0.062	25564.3	0.0541
58	RRKMSPARP	0.0058	0.2689	0.026	37535.94	0.023	0.0051	0.2624	0.046	30396.07	0.0399	0.006	0.2707	0.013	43675	0.0119
59	RKMSPARPR	0.0057	0.2686	0.044	30893.41	0.0383	0.0271	0.3843	0.077	21617.22	0.0695	0.0069	0.2788	0.033	35176.57	0.0291
60	KMSPARPRT	0.2208	0.9178	0.183	6903.33	0.1887	0.0249	0.3755	0.107	15795.37	0.0947	0.0087	0.2921	0.04	32434.8	0.0353
61	MSPARPRTS	0.0053	0.2643	0.041	31912.64	0.0356	0.0055	0.2668	0.051	28795.36	0.0442	0.0102	0.3026	0.048	29745.37	0.0423
**62**	**SPARPRTSC**	**0.0063**	**0.2739**	**0.03**	**35946.07**	**0.0265**	**0.7616**	**5.0897**	**0.712**	**22.56**	**0.7194**	**0.0467**	**0.4524**	**0.098**	**17410.87**	**0.0903**

63	PARPRTSCR	0.0043	0.2546	0.024	38565.12	0.021	0.0373	0.4215	0.054	27725.29	0.0515	0.0356	0.4156	0.025	38150.11	0.0266
64	ARPRTSCRE	0.0066	0.2765	0.024	38565.12	0.0214	0.0038	0.2489	0.039	32787.64	0.0337	0.0051	0.2623	0.013	43439.36	0.0118
65	RPRTSCREA	0.0069	0.2784	0.037	33504.88	0.0325	0.6382	3.0866	0.728	18.97	0.7145	0.0273	0.3852	0.065	24747.82	0.0594
66	PRTSCREAC	0.0079	0.2864	0.024	38774.32	0.0216	0.0106	0.3046	0.047	30068.96	0.0415	0.0074	0.2823	0.013	43439.36	0.0122
67	RTSCREACL	0.0662	0.5106	0.138	11233.42	0.1272	0.0796	0.5478	0.175	7486.88	0.1607	0.0156	0.333	0.058	26694.99	0.0516
68	TSCREACLQ	0.0047	0.2591	0.028	36931.66	0.0245	0.0043	0.2545	0.036	34053.09	0.0312	0.0563	0.4818	0.023	38984.65	0.028
69	SCREACLQG	0.0084	0.2901	0.031	35752.13	0.0276	0.0243	0.373	0.044	31060.99	0.041	0.0142	0.3259	0.032	35367.39	0.0293
70	CREACLQGW	0.0051	0.2627	0.038	33144.32	0.0331	0.0049	0.2605	0.057	26839.8	0.0492	0.0081	0.288	0.029	36337.11	0.0259
71	REACLQGWT	0.0072	0.2808	0.044	30893.41	0.0385	0.0062	0.2731	0.054	27725.29	0.0468	0.0082	0.2885	0.019	40708.92	0.0174
72	EACLQGWTE	0.0044	0.2556	0.026	37739.56	0.0228	0.0065	0.2752	0.038	33144.32	0.0333	0.1002	0.6026	0.064	25017.04	0.0694
73	ACLQGWTEA	0.0541	0.4752	0.103	16404.99	0.0957	0.0097	0.2989	0.067	24218.04	0.0584	0.0076	0.2844	0.049	29584.89	0.0428
74	RRMMRTKMRM	0.0354	0.4149	0.071	23192.26	0.0657	0.0354	0.415	0.207	5353.44	0.1813	0.0146	0.3278	0.053	28331.79	0.0472
75	RMMRTKMRMR	0.0458	0.4496	0.092	18478.42	0.0851	0.0111	0.3078	0.073	22818.9	0.0637	0.0058	0.2696	0.032	35367.39	0.0281
76	MMRTKMRMRR	0.0077	0.2845	0.056	27131.77	0.0487	0.0109	0.3069	0.064	25017.04	0.056	0.0087	0.2925	0.041	32085.75	0.0362
77	MRTKMRMRRM	0.011	0.3073	0.037	33504.88	0.0331	0.0126	0.3171	0.12	13575.13	0.1039	0.0276	0.3863	0.088	19191.6	0.0789
78	RTKMRMRRMR	0.0048	0.2595	0.026	37739.56	0.0228	0.0111	0.3083	0.071	23318.07	0.062	0.0046	0.2573	0.015	42280.1	0.0134
79	TKMRMRRMRR	0.006	0.2713	0.024	38565.12	0.0213	0.0069	0.2787	0.042	31569.21	0.0367	0.0081	0.2878	0.02	40270.83	0.0182
80	KMRMRRMRRT	0.0224	0.3648	0.062	25564.3	0.0561	0.0445	0.4454	0.159	8950.22	0.1418	0.0049	0.2605	0.016	42051.98	0.0143
81	MRMRRMRRTR	0.0057	0.2686	0.033	34986.78	0.0289	0.0077	0.2849	0.059	26265.23	0.0513	0.0067	0.2768	0.034	34798.02	0.0299
82	RMRRMRRTRR	0.0063	0.2737	0.037	33324.12	0.0324	0.0357	0.4158	0.129	12449.51	0.115	0.0045	0.256	0.024	38565.12	0.0211
83	MRRMRRTRRK	0.004	0.2506	0.019	40489.28	0.0167	0.0076	0.2842	0.076	21970.92	0.0657	0.0053	0.2642	0.021	39622.53	0.0186
84	RRMRRTRRKM	0.0143	0.3266	0.044	30893.41	0.0396	0.0758	0.5375	0.247	3435.38	0.2213	0.0065	0.2754	0.035	34237.82	0.0307

85	RMRRTRRKMR	0.0062	0.2727	0.029	36534.22	0.0256	0.0392	0.4281	0.11	15208.39	0.0994	0.0044	0.2558	0.019	40708.92	0.0168
86	MRRTRRKMRR	0.004	0.2513	0.02	40270.83	0.0176	0.0075	0.2834	0.043	31229.48	0.0377	0.0064	0.2742	0.018	40929.75	0.0163
87	RRTRRKMRRK	0.0047	0.2584	0.019	40489.28	0.0169	0.0054	0.2652	0.041	31912.64	0.0357	0.0043	0.2544	0.013	43204.99	0.0117
88	RTRRKMRRKM	0.0072	0.2814	0.035	34423.54	0.0308	0.3006	1.1535	0.475	293.06	0.4488	0.0061	0.2718	0.03	35946.07	0.0264
89	TRRKMRRKMS	0.0045	0.2568	0.017	41825.1	0.0151	0.0132	0.3204	0.051	28795.36	0.0453	0.0052	0.2635	0.013	43439.36	0.0118
90	RRKMRRKMSP	0.0053	0.2644	0.019	40489.28	0.0169	0.0114	0.3098	0.066	24614.3	0.0578	0.0046	0.2575	0.012	43911.91	0.0109
91	RKMRRKMSPA	0.0461	0.4506	0.076	21852.38	0.0715	0.0644	0.5054	0.225	4382.3	0.2009	0.0065	0.2755	0.03	35946.07	0.0265
92	KMRRKMSPAR	0.0143	0.3261	0.052	28331.79	0.0463	0.0463	0.4511	0.1	17038.15	0.0919	0.0055	0.2667	0.04	32610.74	0.0348
93	MRRKMSPARP	0.0062	0.2728	0.021	39837.46	0.0188	0.0076	0.2838	0.052	28331.79	0.0453	0.0107	0.3054	0.032	35367.39	0.0288
94	RRKMSPARPR	0.0041	0.2527	0.022	39196.13	0.0193	0.0081	0.2875	0.05	28951.56	0.0437	0.0052	0.2638	0.018	41151.77	0.0161
95	RKMSPARPRT	0.0247	0.3745	0.04	32434.8	0.0377	0.0344	0.4113	0.112	14882.82	0.1004	0.0084	0.2902	0.024	38774.32	0.0217
96	KMSPARPRTS	0.0645	0.5058	0.094	18082.85	0.0896	0.0302	0.396	0.076	21852.38	0.0691	0.0072	0.2809	0.04	32610.74	0.0351
97	MSPARPRTSC	0.0069	0.2783	0.04	32610.74	0.035	0.016	0.3353	0.081	20813.9	0.0713	0.01	0.3011	0.041	32085.75	0.0364
98	SPARPRTSCR	0.0051	0.2628	0.022	39196.13	0.0195	0.3353	1.2687	0.264	2889.29	0.2747	0.1042	0.613	0.087	19505.61	0.0896
99	PARPRTSCRE	0.0044	0.2558	0.015	42280.1	0.0134	0.0271	0.3843	0.038	32965.5	0.0364	0.0412	0.4347	0.018	41151.77	0.0215
100	ARPRTSCREA	0.0139	0.3242	0.027	37333.42	0.025	0.005	0.262	0.04	32434.8	0.0348	0.0048	0.2599	0.015	42280.1	0.0135
101	RPRTSCREAC	0.0068	0.2775	0.024	38774.32	0.0214	0.7779	5.5197	0.692	28.16	0.7049	0.0326	0.4051	0.068	24087.38	0.0627
102	PRTSCREACL	0.0106	0.305	0.022	39196.13	0.0203	0.0127	0.3177	0.044	31229.48	0.0393	0.0114	0.3101	0.019	40489.28	0.0179
103	RTSCREACLQ	0.0083	0.2896	0.031	35752.13	0.0276	0.0113	0.3094	0.041	32085.75	0.0365	0.0135	0.3221	0.026	37739.56	0.0241
104	TSCREACLQG	0.0058	0.2693	0.026	37739.56	0.023	0.005	0.2612	0.026	37739.56	0.0228	0.0343	0.4109	0.029	36337.11	0.0298
105	SCREACLQGW	0.0068	0.2781	0.029	36534.22	0.0257	0.0221	0.3635	0.051	28951.56	0.0467	0.0166	0.3384	0.046	30232.07	0.0416
106	CREACLQGWT	0.0059	0.2696	0.029	36534.22	0.0255	0.0038	0.249	0.037	33686.63	0.032	0.0077	0.2849	0.017	41825.1	0.0156

107	REACLQGWTE	0.0078	0.2855	0.03	36141.06	0.0267	0.0091	0.2948	0.043	31569.21	0.0379	0.0101	0.3016	0.028	36931.66	0.0253
108	EACLQGWTEA	0.0055	0.2669	0.044	31060.99	0.0382	0.0089	0.2937	0.05	29266.51	0.0438	0.058	0.4868	0.1	16946.23	0.0937
109	RRMMRTKMRMR	0.0071	0.28	0.039	32787.64	0.0342	0.0082	0.2886	0.064	25152.75	0.0556	0.0051	0.2628	0.02	40270.83	0.0178
110	RMMRTKMRMRR	0.0514	0.4669	0.102	16583.46	0.0944	0.0105	0.3042	0.075	22090.1	0.0653	0.0063	0.2738	0.039	32787.64	0.0341
111	MMRTKMRMRRM	0.0256	0.3783	0.131	12183	0.1152	0.0927	0.5828	0.466	324.79	0.41	0.0233	0.3686	0.179	7208.66	0.1556
112	MRTKMRMRRMR	0.0049	0.2611	0.027	37535.94	0.0237	0.0048	0.2592	0.054	27875.69	0.0466	0.0075	0.2831	0.022	39196.13	0.0198
113	RTKMRMRRMRR	0.0049	0.261	0.033	35176.57	0.0288	0.0095	0.2979	0.069	23828.16	0.0601	0.0048	0.2592	0.016	42051.98	0.0143
114	TKMRMRRMRRT	0.011	0.3076	0.034	34798.02	0.0306	0.0099	0.3002	0.063	25289.19	0.055	0.0079	0.2863	0.018	41151.77	0.0165
115	KMRMRRMRRTR	0.0081	0.2882	0.039	32787.64	0.0344	0.0444	0.445	0.117	14099.07	0.1061	0.0047	0.2585	0.027	37333.42	0.0237
116	MRMRRMRRTRR	0.0055	0.2661	0.037	33504.88	0.0323	0.0056	0.2676	0.06	26123.53	0.0518	0.0069	0.2788	0.025	38150.11	0.0223
117	RMRRMRRTRRK	0.0068	0.2774	0.034	34610.27	0.0299	0.0298	0.3944	0.146	10301.97	0.1286	0.0043	0.2548	0.025	38357.05	0.0219
118	MRRMRRTRRKM	0.0051	0.2624	0.031	35752.13	0.0271	0.0667	0.5121	0.254	3202.08	0.2259	0.0098	0.2996	0.057	26985.39	0.0499
119	RRMRRTRRKMR	0.0061	0.2719	0.035	34423.54	0.0307	0.0114	0.3098	0.064	25017.04	0.0561	0.0046	0.2581	0.018	41375	0.016
120	RMRRTRRKMRR	0.0081	0.2882	0.039	32787.64	0.0344	0.0251	0.3762	0.09	18882.64	0.0803	0.0047	0.2582	0.024	38565.12	0.0211
121	MRRTRRKMRRK	0.0039	0.2498	0.022	39196.13	0.0193	0.0076	0.2844	0.072	23067.13	0.0623	0.0051	0.2624	0.018	41375	0.0161
122	RRTRRKMRRKM	0.0085	0.2904	0.036	33869.37	0.0319	0.037	0.4204	0.115	14329.76	0.1033	0.0066	0.2764	0.027	37535.94	0.0239
123	RTRRKMRRKMS	0.0057	0.2685	0.026	37739.56	0.023	0.0579	0.4865	0.131	12117.27	0.12	0.0046	0.2577	0.013	43439.36	0.0117
124	TRRKMRRKMSP	0.0048	0.2595	0.022	39408.75	0.0194	0.0136	0.3227	0.058	26550.96	0.0513	0.0056	0.2671	0.015	42740.04	0.0136
125	RRKMRRKMSPA	0.0077	0.2848	0.039	32787.64	0.0343	0.0259	0.3793	0.133	11857.88	0.1169	0.0048	0.2593	0.017	41825.1	0.0152
126	RKMRRKMSPAR	0.0106	0.3047	0.038	32965.5	0.0339	0.0233	0.3688	0.073	22695.78	0.0655	0.0066	0.2763	0.034	34798.02	0.0299
127	KMRRKMSPARP	0.0288	0.3908	0.051	28795.36	0.0477	0.0246	0.3743	0.079	21154.46	0.0708	0.0059	0.2699	0.018	40929.75	0.0162
128	MRRKMSPARPR	0.0045	0.2563	0.029	36337.11	0.0253	0.0117	0.3115	0.077	21617.22	0.0672	0.0078	0.2852	0.046	30232.07	0.0403

129	RRKMSPARPRT	0.0071	0.2805	0.026	37944.28	0.0232	0.0095	0.298	0.068	24087.38	0.0592	0.0058	0.2688	0.014	42971.89	0.0128
130	RKMSPARPRTS	0.0093	0.2966	0.037	33504.88	0.0328	0.0384	0.4253	0.089	19088.05	0.0814	0.0067	0.2773	0.026	37739.56	0.0231
131	KMSPARPRTSC	0.1713	0.7857	0.119	13872.1	0.1268	0.1427	0.712	0.146	10357.86	0.1455	0.0071	0.2803	0.04	32610.74	0.0351
132	MSPARPRTSCR	0.0051	0.2629	0.036	33869.37	0.0314	0.0048	0.2597	0.053	28178.94	0.0458	0.017	0.3402	0.048	29906.73	0.0433
133	SPARPRTSCRE	0.0054	0.2652	0.022	39622.53	0.0195	0.2456	0.9875	0.283	2327.08	0.2774	0.1028	0.6093	0.082	20478.82	0.0851
134	PARPRTSCREA	0.0057	0.2681	0.036	34053.09	0.0315	0.0661	0.5103	0.13	12249.09	0.1204	0.0296	0.3937	0.033	34986.78	0.0325
135	ARPRTSCREAC	0.0118	0.3123	0.026	37739.56	0.0239	0.011	0.3072	0.05	29108.61	0.0441	0.0052	0.2635	0.015	42740.04	0.0135
136	RPRTSCREACL	0.0096	0.2982	0.044	30893.41	0.0388	0.7928	5.9692	0.802	8.52	0.8006	0.0931	0.5839	0.134	11793.9	0.1279
137	PRTSCREACLQ	0.0045	0.2569	0.019	40708.92	0.0168	0.0048	0.2594	0.041	32085.75	0.0356	0.0106	0.3051	0.013	43675	0.0126
138	RTSCREACLQG	0.0139	0.3241	0.041	32085.75	0.0369	0.0137	0.323	0.053	28026.9	0.0471	0.0086	0.2916	0.031	35752.13	0.0276
139	TSCREACLQGW	0.0052	0.2632	0.037	33686.63	0.0322	0.0048	0.2595	0.038	33144.32	0.033	0.0461	0.4506	0.042	31740.46	0.0426
140	SCREACLQGWT	0.0091	0.2949	0.043	31398.89	0.0379	0.015	0.33	0.06	25982.58	0.0532	0.0122	0.3144	0.026	37944.28	0.0239
141	CREACLQGWTE	0.0059	0.2701	0.026	37739.56	0.023	0.0045	0.2565	0.042	31740.46	0.0364	0.0095	0.2977	0.024	38565.12	0.0218
142	REACLQGWTEA	0.0122	0.3146	0.061	25842.4	0.0537	0.0139	0.3244	0.07	23444.56	0.0616	0.0074	0.2826	0.037	33686.63	0.0326

Supplementary Table 8. Association analysis of *HLA-I* alleles with CALRmut+ MPNs *versus* healthy controls in the Bulgarian population.

allele	p.value	p.adjusted	estimate	std.error	conf.low	conf.high	statistic	Ntotal	Ntotal.percent	N(disease=0)	N(disease=0).percent	N(disease=1)	N(disease=1).percent
C*06:02	0.046413	1	0.290971	0.619865	0.068223	0.85243	-1.99162	180	8.00%	177	8.17%	3	3.57%
A*23:01	0.052002	1	3.564332	0.654091	0.863616	11.744	1.94312	51	2.27%	47	2.17%	4	4.76%
B*13:02	0.092005	1	0.182105	1.010836	0.010256	0.884195	-1.68492	67	2.98%	66	3.05%	1	1.19%
B*52:01	0.1001	1	3.243306	0.715529	0.66295	11.81095	1.644369	41	1.82%	38	1.75%	3	3.57%
C*12:02	0.108976	1	3.128405	0.711575	0.643268	11.29108	1.602815	40	1.78%	37	1.71%	3	3.57%
A*11:01	0.112284	1	1.939032	0.416993	0.808603	4.238769	1.58801	183	8.13%	174	8.03%	9	10.71%
B*41:01	0.130556	1	3.850673	0.891749	0.499386	18.59277	1.511915	25	1.11%	23	1.06%	2	2.38%
B*35:03	0.141624	1	2.083081	0.499294	0.685499	5.105742	1.469771	101	4.49%	96	4.43%	5	5.95%
A*68:01	0.178427	1	2.171605	0.576292	0.639674	6.484698	1.345613	59	2.62%	54	2.49%	5	5.95%
C*16:02	0.255298	1	0.255249	1.200375	0.011787	1.930017	-1.13757	28	1.24%	27	1.25%	1	1.19%
B*51:01	0.265053	1	1.444782	0.330148	0.737058	2.713598	1.114527	275	12.22%	257	11.87%	18	21.43%
C*07:01	0.301913	1	0.689296	0.360429	0.324897	1.345738	-1.03234	388	17.24%	376	17.36%	12	14.29%
B*44:02	0.302618	1	0.338294	1.051419	0.01834	1.73985	-1.03083	92	4.09%	91	4.20%	1	1.19%
A*32:01	0.356386	1	1.574295	0.492053	0.546458	3.851722	0.922274	117	5.20%	110	5.08%	7	8.33%
C*01:02	0.376443	1	1.559457	0.502378	0.542756	4.021052	0.884469	90	4.00%	84	3.88%	6	7.14%
B*39:01	0.396788	1	1.864408	0.735148	0.330507	7.383404	0.847371	39	1.73%	37	1.71%	2	2.38%
C*05:01	0.402621	1	0.412923	1.056805	0.022266	2.159701	-0.83695	78	3.47%	77	3.55%	1	1.19%
C*12:03	0.436401	1	1.361422	0.396422	0.589466	2.841005	0.778285	242	10.76%	233	10.76%	9	10.71%
B*07:02	0.439481	1	1.645231	0.64403	0.370662	5.004342	0.773071	96	4.27%	93	4.29%	3	3.57%

A*26:01	0.457091	1	0.599928	0.687083	0.125453	1.976741	-0.74364	114	5.07%	111	5.12%	3	3.57%
A*02:01	0.492321	1	0.816319	0.295578	0.447014	1.432831	-0.68662	643	28.58%	621	28.67%	22	26.19%
A*24:02	0.500097	1	0.740687	0.445144	0.282917	1.654442	-0.67434	297	13.20%	290	13.39%	7	8.33%
A*01:01	0.526276	1	0.782682	0.386664	0.352892	1.620287	-0.6337	288	12.80%	276	12.74%	12	14.29%
B*40:02	0.547888	1	1.605527	0.787869	0.240748	6.097299	0.600927	50	2.22%	48	2.22%	2	2.38%
B*56:01	0.566126	1	1.687003	0.91144	0.213865	8.568487	0.573767	27	1.20%	25	1.15%	2	2.38%
C*04:01	0.568173	1	1.203358	0.324341	0.621008	2.231131	0.570744	389	17.29%	374	17.27%	15	17.86%
B*55:01	0.598852	1	1.762221	1.077032	0.09317	9.814742	0.526052	38	1.69%	37	1.71%	1	1.19%
B*58:01	0.602271	1	1.563635	0.857766	0.210807	6.779303	0.521137	32	1.42%	30	1.39%	2	2.38%
B*15:01	0.627998	1	1.494103	0.828662	0.209288	6.07332	0.484547	43	1.91%	41	1.89%	2	2.38%
B*18:01	0.630052	1	0.815033	0.424635	0.324702	1.757525	-0.48165	250	11.11%	242	11.17%	8	9.52%
B*44:03	0.631831	1	1.531989	0.890255	0.193505	7.036266	0.479152	53	2.36%	51	2.35%	2	2.38%
A*25:01	0.677167	1	1.43964	0.875246	0.189781	6.619494	0.416332	40	1.78%	38	1.75%	2	2.38%
A*03:01	0.705875	1	1.177481	0.4329	0.465278	2.609288	0.377401	205	9.11%	198	9.14%	7	8.33%
C*02:02	0.728933	1	1.174356	0.463776	0.447542	2.806236	0.346545	194	8.62%	187	8.63%	7	8.33%
B*38:01	0.770072	1	0.828156	0.64511	0.187244	2.533875	-0.29228	96	4.27%	93	4.29%	3	3.57%
B*57:01	0.827048	1	0.789763	1.080249	0.041661	4.453087	-0.21849	44	1.96%	43	1.99%	1	1.19%
B*50:01	0.841984	1	0.795508	1.147566	0.039091	5.317763	-0.19936	26	1.16%	25	1.15%	1	1.19%
C*03:03	0.868803	1	0.87275	0.823992	0.124469	3.608918	-0.16518	58	2.58%	56	2.59%	2	2.38%
C*14:02	0.910086	1	1.086759	0.736739	0.20284	3.922721	0.11293	72	3.20%	69	3.19%	3	3.57%
B*27:05	0.91766	1	0.916067	0.847977	0.126303	3.995529	-0.10338	50	2.22%	48	2.22%	2	2.38%
C*07:04	0.93755	1	0.918137	1.090098	0.047863	5.26618	-0.07835	30	1.33%	29	1.34%	1	1.19%

B*35:01	0.95712	1	0.973857	0.492683	0.334268	2.368632	-0.05377	173	7.69%	167	7.71%	6	7.14%
B*08:01	0.981635	1	0.986761	0.578951	0.27234	2.773447	-0.02302	143	6.36%	139	6.42%	4	4.76%
A*30:01	0.98543	1	1.18E-07	873.7102	4.69E-134	1.08E+09	-0.01826	38	1.69%	38	1.75%	0	0.00%
B*44:05	0.986413	1	1.74E-07	913.8964	5.39E-139	1.16E+10	-0.01703	38	1.69%	38	1.75%	0	0.00%
B*35:08	0.987158	1	4.83E-07	903.5113	2.42E-136	2.8E+10	-0.0161	45	2.00%	45	2.08%	0	0.00%
C*03:04	0.987522	1	4.84E-07	929.7818	1.61E-141	4.76E+10	-0.01564	43	1.91%	43	1.99%	0	0.00%
B*37:01	0.987787	1	1.91E-07	1010.56	4.47E-152	1.07E+12	-0.01531	31	1.38%	31	1.43%	0	0.00%
C*08:02	0.989273	1	3.76E-07	1100.379	2.66E-166	4.84E+13	-0.01345	31	1.38%	31	1.43%	0	0.00%
B*14:02	0.989276	1	3.75E-07	1100.925	2.21E-166	4.94E+13	-0.01344	31	1.38%	31	1.43%	0	0.00%
B*27:02	0.989764	1	1.01432	1.108263	0.051716	6.014996	0.012829	44	1.96%	43	1.99%	1	1.19%
B*40:01	0.989956	1	4.08E-07	1168.633	3.92E-176	9.32E+14	-0.01259	26	1.16%	26	1.20%	0	0.00%
B*49:01	0.995781	1	1.004456	0.840824	0.16771	4.655074	0.005288	40	1.78%	37	1.71%	3	3.57%

Supplementary Table 9. Association analysis of *HLA-I* alleles with CALRmut+ MPNs *versus* JAK2 V617F+ MPNs in the Bulgarian population.

allele	p.value	p.adjusted	estimate	std.error	conf.low	conf.high	statistic	Ntotal	Ntotal.percent	N(disease=0)	N(disease=0).percent	N(disease=1)	N(disease=1).percent
C*15:02	0.001676	0.077103	4.534906	0.481111	1.754435	11.78775	3.142318	22	5.50%	11	3.48%	11	13.10%
A*11:01	0.131788	1	1.925864	0.434861	0.790531	4.457227	1.50709	29	7.25%	20	6.33%	9	10.71%
A*24:02	0.140898	1	0.53069	0.430286	0.209778	1.160457	-1.47246	54	13.50%	47	14.87%	7	8.33%
B*51:01	0.160249	1	1.577689	0.324704	0.82315	2.970468	1.404236	67	16.75%	49	15.51%	18	21.43%
A*03:02	0.168404	1	3.712101	0.952262	0.462053	24.16676	1.37735	5	1.25%	3	0.95%	2	2.38%
B*35:01	0.178716	1	2.08397	0.546043	0.672442	5.928383	1.34472	17	4.25%	11	3.48%	6	7.14%
B*15:01	0.311324	1	2.645199	0.960787	0.324116	17.46116	1.012447	5	1.25%	3	0.95%	2	2.38%
C*05:01	0.321873	1	0.34775	1.066278	0.018622	1.910265	-0.99062	11	2.75%	10	3.16%	1	1.19%
B*44:02	0.336096	1	0.358608	1.066137	0.019207	1.970165	-0.96191	11	2.75%	10	3.16%	1	1.19%
B*13:02	0.362039	1	0.378877	1.064791	0.020315	2.073167	-0.91149	12	3.00%	11	3.48%	1	1.19%
A*23:01	0.372459	1	1.762392	0.63537	0.452476	5.831724	0.891877	13	3.25%	9	2.85%	4	4.76%
B*55:01	0.406281	1	0.405827	1.085944	0.021312	2.364867	-0.83046	9	2.25%	8	2.53%	1	1.19%
C*06:02	0.41065	1	0.594389	0.632296	0.137192	1.78676	-0.82275	24	6.00%	21	6.65%	3	3.57%
B*18:01	0.419348	1	0.714852	0.415675	0.295456	1.538124	-0.80755	49	12.25%	41	12.97%	8	9.52%
B*08:01	0.439653	1	0.648988	0.559463	0.184889	1.759611	-0.77278	26	6.50%	22	6.96%	4	4.76%
B*27:02	0.454389	1	0.445534	1.080686	0.023539	2.567468	-0.74812	9	2.25%	8	2.53%	1	1.19%
B*58:01	0.468002	1	1.915322	0.895488	0.254771	10.42711	0.725734	6	1.50%	4	1.27%	2	2.38%
C*12:03	0.492517	1	0.769968	0.380886	0.342371	1.55449	-0.68631	52	13.00%	43	13.61%	9	10.71%
A*02:01	0.517145	1	1.197472	0.278212	0.683049	2.048767	0.647753	95	23.75%	73	23.10%	22	26.19%

C*07:01	0.535472	1	0.795349	0.369507	0.371086	1.597682	-0.61968	66	16.50%	54	17.09%	12	14.29%
B*27:05	0.618632	1	0.670122	0.804145	0.099079	2.727525	-0.49779	12	3.00%	10	3.16%	2	2.38%
C*17:01	0.643412	1	0.592118	1.132017	0.029674	3.989408	-0.46293	6	1.50%	5	1.58%	1	1.19%
B*38:01	0.643855	1	0.74423	0.63897	0.16986	2.283272	-0.46232	18	4.50%	15	4.75%	3	3.57%
A*32:01	0.651589	1	1.221064	0.442298	0.477321	2.800166	0.451555	29	7.25%	22	6.96%	7	8.33%
C*02:02	0.667809	1	1.216318	0.456309	0.463942	2.865846	0.429157	30	7.50%	23	7.28%	7	8.33%
C*01:02	0.668384	1	1.244753	0.511097	0.423111	3.240111	0.428367	25	6.25%	19	6.01%	6	7.14%
C*03:03	0.688505	1	0.723874	0.806063	0.106796	2.965142	-0.40088	12	3.00%	10	3.16%	2	2.38%
C*07:04	0.784661	1	0.735448	1.124524	0.037213	4.899975	-0.27325	6	1.50%	5	1.58%	1	1.19%
B*40:02	0.799178	1	1.235553	0.831403	0.177169	5.493271	0.254411	9	2.25%	7	2.22%	2	2.38%
B*41:01	0.822623	1	1.216757	0.87517	0.164801	6.103304	0.224172	7	1.75%	5	1.58%	2	2.38%
A*68:01	0.831559	1	1.116765	0.519205	0.361142	2.921087	0.212702	21	5.25%	16	5.06%	5	5.95%
C*07:02	0.854297	1	0.884336	0.669348	0.194732	2.940152	-0.18364	16	4.00%	13	4.11%	3	3.57%
C*12:02	0.901356	1	0.918432	0.68648	0.196993	3.177113	-0.12395	14	3.50%	11	3.48%	3	3.57%
B*44:03	0.945488	1	0.94439	0.836817	0.133926	4.214385	-0.06837	9	2.25%	7	2.22%	2	2.38%
C*16:02	0.945967	1	0.92606	1.133448	0.046585	6.502128	-0.06777	5	1.25%	4	1.27%	1	1.19%
B*49:01	0.947728	1	0.959326	0.63338	0.22173	2.956092	-0.06556	16	4.00%	13	4.11%	3	3.57%
B*52:01	0.974775	1	1.022197	0.694296	0.216933	3.612263	0.03162	13	3.25%	10	3.16%	3	3.57%
B*07:02	0.97543	1	0.979416	0.675306	0.213991	3.310215	-0.0308	15	3.75%	12	3.80%	3	3.57%
B*50:01	0.981566	1	1.026749	1.142452	0.051164	7.37249	0.023106	5	1.25%	4	1.27%	1	1.19%
B*35:02	0.987381	1	2.16E-07	970.4615	NA	5.10E+33	-0.01582	6	1.50%	6	1.90%	0	0.00%
B*14:02	0.988191	1	1.80E-07	1049.312	NA	3.40E+40	-0.0148	5	1.25%	5	1.58%	0	0.00%

A*31:01	0.988382	1	1.93E-07	1061.799	NA	1.34E+41	-0.01456	5	1.25%	5	1.58%	0	0.00%
A*30:01	0.988737	1	2.79E-07	1069.157	NA	3.74E+41	-0.01412	5	1.25%	5	1.58%	0	0.00%
C*14:02	0.988772	1	0.990501	0.678189	0.215492	3.369707	-0.01407	15	3.75%	12	3.80%	3	3.57%
B*35:03	0.993169	1	1.004434	0.516703	0.32566	2.596278	0.008562	24	6.00%	19	6.01%	5	5.95%
A*25:01	0.995908	1	0.995762	0.828114	0.143264	4.380021	-0.00513	9	2.25%	7	2.22%	2	2.38%

Supplementary Table 10. Association analysis of *HLA-II* alleles with CALRmut+ MPNs *versus* healthy controls in the Bulgarian population.

allele	p.value	p.adjusted	estimate	std.error	conf.low	conf.high	statistic	Ntotal	Ntotal.percent	N(disease=0)	N(disease=0).percent	N(disease=1)	N(disease=1).percent
DQA1*01:02	0.002523	0.123635	2.41739	0.292228	1.352452	4.285106	3.02055	440	19.56%	415	19.16%	25	29.76%
DQB1*05:02	0.017973	0.880696	2.109064	0.315381	1.115165	3.872955	2.366166	340	15.11%	321	14.82%	19	22.62%
DQA1*04:01	0.021377	1	4.823516	0.683756	1.257054	17.7526	2.301265	32	1.42%	27	1.25%	5	5.95%
DRB1*16:01	0.037243	1	2.067556	0.348697	1.012411	4.013039	2.083092	288	12.80%	273	12.60%	15	17.86%
DRB1*08:01	0.042213	1	3.644641	0.636631	0.922401	11.75561	2.03141	39	1.73%	35	1.62%	4	4.76%
DQA1*05:05	0.076914	1	0.528843	0.36015	0.250014	1.033943	-1.76888	446	19.82%	432	19.94%	14	16.67%
DRB1*13:01	0.08919	1	0.159079	1.081585	0.008345	0.876361	-1.69969	125	5.56%	124	5.72%	1	1.19%
DRB1*15:01	0.097733	1	2.034968	0.429048	0.819386	4.546356	1.655945	131	5.82%	124	5.72%	7	8.33%
DQB1*03:01	0.101414	1	0.591962	0.320087	0.306535	1.081773	-1.63804	574	25.51%	557	25.72%	17	20.24%
DRB1*11:04	0.108356	1	0.493449	0.439913	0.196157	1.120836	-1.60563	294	13.07%	287	13.25%	7	8.33%
DPB1*05:01	0.144295	1	3.00625	0.753909	0.567713	11.63237	1.459983	33	1.47%	30	1.39%	3	3.57%
DQB1*06:02	0.166765	1	2.13133	0.547306	0.62443	5.762396	1.382675	87	3.87%	83	3.83%	4	4.76%
DQA1*05:01	0.173245	1	1.669309	0.37626	0.768817	3.401857	1.361852	181	8.04%	170	7.85%	11	13.10%
DRB1*04:02	0.231231	1	1.939336	0.553249	0.599954	5.551804	1.197193	56	2.49%	51	2.35%	5	5.95%
DQB1*06:03	0.232112	1	0.459499	0.650762	0.101739	1.39676	-1.19493	144	6.40%	141	6.51%	3	3.57%
DQB1*06:01	0.238958	1	2.624419	0.819348	0.379342	10.94319	1.177595	40	1.78%	38	1.75%	2	2.38%
DQB1*02:01	0.258987	1	1.521745	0.371954	0.706858	3.077171	1.128788	218	9.69%	207	9.56%	11	13.10%
DRB1*03:01	0.262711	1	1.515551	0.371229	0.704076	3.055216	1.120007	206	9.16%	195	9.00%	11	13.10%

DPB1*10:01	0.285372	1	1.891297	0.596504	0.521731	5.513924	1.068329	61	2.71%	56	2.59%	5	5.95%
DRB1*15:02	0.316125	1	2.242361	0.805553	0.330095	9.046168	1.002454	46	2.04%	44	2.03%	2	2.38%
DRB1*12:01	0.387234	1	2.545869	1.080759	0.134223	14.4197	0.864644	30	1.33%	29	1.34%	1	1.19%
DPB1*23:01	0.388398	1	2.227094	0.928317	0.257251	10.88032	0.862526	31	1.38%	29	1.34%	2	2.38%
DRB1*04:01	0.435354	1	0.404223	1.161173	0.019278	2.620322	-0.78006	43	1.91%	42	1.94%	1	1.19%
DQA1*03:03	0.472277	1	0.524774	0.89706	0.066852	2.523345	-0.71878	49	2.18%	47	2.17%	2	2.38%
DQA1*01:04	0.48052	1	0.61052	0.699459	0.125737	2.07418	-0.70547	96	4.27%	93	4.29%	3	3.57%
DQB1*02:02	0.490167	1	1.459736	0.548162	0.43876	3.886401	0.690043	127	5.64%	122	5.63%	5	5.95%
DQB1*05:01	0.519512	1	0.708191	0.535696	0.218951	1.844043	-0.6441	204	9.07%	199	9.19%	5	5.95%
DRB1*07:01	0.528174	1	0.750225	0.455587	0.287488	1.772096	-0.6308	162	7.20%	156	7.20%	6	7.14%
DQB1*05:03	0.568642	1	0.67984	0.676951	0.144201	2.187807	-0.57005	121	5.38%	118	5.45%	3	3.57%
DRB1*14:01	0.580106	1	0.545426	1.095723	0.02819	3.140559	-0.55323	45	2.00%	44	2.03%	1	1.19%
DQA1*02:01	0.639369	1	0.775019	0.543913	0.244606	2.142935	-0.46858	128	5.69%	123	5.68%	5	5.95%
DPB1*04:01	0.669889	1	0.886091	0.283687	0.499423	1.527739	-0.4263	774	34.40%	749	34.58%	25	29.76%
DPB1*01:01	0.672443	1	0.719593	0.778315	0.109221	2.671603	-0.4228	87	3.87%	85	3.92%	2	2.38%
DRB1*16:02	0.692892	1	1.384906	0.824526	0.198362	5.81042	0.394933	31	1.38%	29	1.34%	2	2.38%
DRB1*13:02	0.728482	1	0.698295	1.034475	0.038533	3.442468	-0.34715	76	3.38%	75	3.46%	1	1.19%
DQB1*04:02	0.792078	1	1.155905	0.549607	0.3545	3.233386	0.263613	55	2.44%	50	2.31%	5	5.95%
DQA1*03:01	0.847632	1	1.091706	0.456652	0.40904	2.511424	0.192141	146	6.49%	139	6.42%	7	8.33%
DRB1*13:03	0.873432	1	1.217841	1.237157	0.053513	9.461661	0.1593	24	1.07%	23	1.06%	1	1.19%
DQA1*01:01	0.880874	1	1.08974	0.573457	0.315374	3.066919	0.149862	124	5.51%	119	5.49%	5	5.95%
DRB1*11:03	0.924755	1	0.899018	1.127127	0.04487	5.418139	-0.09445	32	1.42%	31	1.43%	1	1.19%

DQB1*06:04	0.932636	1	0.915511	1.044289	0.050063	4.672683	-0.08453	59	2.62%	58	2.68%	1	1.19%
DRB1*01:01	0.937664	1	1.045066	0.563632	0.307342	2.878936	0.078206	138	6.13%	133	6.14%	5	5.95%
DRB1*04:03	0.984263	1	2.97E-07	761.9734	2.57E-116	40450512	-0.01973	59	2.62%	59	2.72%	0	0.00%
DRB1*14:54	0.986476	1	3.89E-07	870.6864	4.51E-133	3.17E+09	-0.01695	49	2.18%	49	2.26%	0	0.00%
DRB1*10:01	0.986914	1	3.22E-07	911.3718	1.19E-137	2.61E+10	-0.0164	42	1.87%	42	1.94%	0	0.00%
DQA1*01:05	0.987888	1	3.11E-07	987.0181	5.38E-150	3.39E+11	-0.01518	35	1.56%	35	1.62%	0	0.00%
DPB1*17:01	0.9882	1	4.89E-07	982.5552	3.81E-149	4.43E+11	-0.01479	38	1.69%	38	1.75%	0	0.00%
DQB1*03:03	0.988744	1	3.03E-07	1063.86	1.71E-159	1.66E+13	-0.01411	30	1.33%	30	1.39%	0	0.00%
DPB1*06:01	0.989858	1	3.24E-07	1175.415	3.22E-177	9.84E+14	-0.01271	26	1.16%	26	1.20%	0	0.00%

Supplementary Table 11. NetMHCIIpan 4.1 predictions of binding for 15-mer peptides from the CALR-mutated sequence to selected HLA-II molecules. Peptides with the lowest predicted binding ranks are highlighted in red.

	HLA-DQA1*0102-DQB10501		HLA-DQA1*0102-DQB10602		HLA-DQA1*0102-DQB10604		HLA-DQA1*0103-DQB10501		HLA-DQA1*0401-DQB10301		HLA-DRB1*0801		HLA-DRB1*1601	
Peptide	Rank	nM	Rank	nM	Rank	nM	Rank	nM	Rank	nM	Rank	nM	Rank	nM
RRMMRTKMRMRRMRR	87.59	1351.309	83.09	2168.365	95	5756.308	61.33	2589.407	95	2473.245	72.47	205.0946	79.46	392.841
RMMRTKMRMRRMRRT	78.41	1049.045	87.62	2058.002	95	5203.19	77.9	2163.093	95	1890.361	69.08	168.3988	86.05	450.682
MMRTKMRMRRMRRTR	81.12	1248.48	84.14	2512.245	95	5789.224	83.31	2460.354	95	1904.383	47.54	193.8258	83.67	471.0229
MRTKMRMRRMRRTRR	80.65	1397.372	72.1	2674.431	95	6024.252	81.02	2741.939	95	1939.63	36.65	167.9694	72.85	418.2609
RTKMRMRRMRRTRRK	79.34	1529.264	61.13	2764.818	95	6311.7	73.15	2974.524	95	1895.112	28.7	151.831	56.76	383.451
TKMRMRRMRRTRRKM	83.49	1938.728	83.22	3176.369	95	7104.282	73.74	3550.027	95	2155.99	38.53	161.7065	59.01	395.1899
KMRMRRMRRTRRKMR	81.76	2226.951	95	3502.261	93.22	7286.525	66.62	3946.832	95	2265.398	39.27	181.1069	53.61	388.1932
MRMRRMRRTRRKMRR	94.71	3266.852	95	5618.716	95	8788.335	90.65	5383.933	95	2858.137	67.66	330.8563	75.54	537.1051
RMRRMRRTRRKMRRK	95	6517.113	95	9670.947	95	12706.44	94.13	9021.389	95	5982.81	67.84	572.8448	78.91	747.9397
MRRMRRTRRKMRRKM	95	6919.63	95	10272.59	95	12981.03	94.05	9370.891	95	6164.767	73.68	746.331	81.97	938.5767
RRMRRTRRKMRRKMS	95	8473.294	95	11783.32	95	14517.97	95	11551.17	95	6807.938	84.95	1030.393	86.89	1284.364
RMRRTRRKMRRKMSP	95	9120.219	95	12390.52	95	15183.4	95	12942.6	95	7524.967	83.24	1244.232	79.93	1671.253
MRRTRRKMRRKMSPA	95	8233.26	95	11874.18	95	13785.31	95	13525.29	95	6618.446	70.66	1241.261	74.87	1851.316
RRTRRKMRRKMSPAR	95	6770.915	95	9404.512	95	11946.61	94.01	11601.02	95	4754.746	53.64	679.9958	68.36	1282.157
RTRRKMRRKMSPARP	95	4882.155	90.17	8229.519	95	10387.48	90.25	8259.134	95	3350.111	39.63	635.7249	54.87	1211.893
TRRKMRRKMSPARPR	89.4	3819.909	80.6	6087.152	95	8378.482	72.92	6601.353	88.64	1777.877	29.76	507.6161	41.96	905.9694
RRKMRRKMSPARPRT	80.74	3889.138	79.16	6079.978	94.35	8486.782	58.26	6540.141	82.04	1675.273	27.5	547.6562	38.71	881.458

RKMRRKMSPARPRTS	70.1	2315.53	14.38	641.9943	82.06	4021.927	56.09	4312.867	22.01	96.04542	39.15	613.002	57.34	983.0523
KMRRKMSPARPRTSC	89.52	2541.773	9.69	524.3916	85.97	4378.315	78.64	4602.688	18.84	89.04359	57.33	808.3869	69.16	1308.462
**MRRKMSPARPRTSCR**	**88.56**	**2602.355**	**4.54**	**392.8537**	77.83	4154.214	84.39	4762.108	11.18	72.45032	56.38	996.3304	56.91	1641.948
**RRKMSPARPRTSCRE**	**82.79**	**2976.52**	**2.68**	**375.1409**	69.24	4288.855	74.49	5386.264	7.49	68.4392	50.62	1669.844	47.59	2293.017
RKMSPARPRTSCREA	95	4108.575	7.51	529.7178	87.19	4978.547	92.8	6974.199	15.9	82.69882	69.59	2730.482	78.23	4071.887
KMSPARPRTSCREAC	95	5855.432	37.57	977.685	95	5521.149	95	9068.462	40.57	119.1294	86.62	4810.216	95	7347.643
MSPARPRTSCREACL	95	6159.966	95	3733.73	95	5845.937	95	9743.417	90.46	329.3883	88.03	5565.412	95	9334.159
SPARPRTSCREACLQ	95	8027.772	95	9853.675	95	7713.327	95	12094.88	90.28	2176.051	89.98	6974.953	95	10414.16
PARPRTSCREACLQG	95	8331.205	95	9483.909	94.48	8270.402	95	12157.99	88.28	2379.457	86.32	7544.369	95	10318.82
ARPRTSCREACLQGW	95	3341.242	95	3453.718	95	3645.115	95	7097.444	94.49	1493.648	90.49	6341.545	95	7356.951
RPRTSCREACLQGWT	95	3026.074	95	2603.791	95	3721.751	95	6681.113	95	1444.911	91.91	6125.34	95	6882.669
PRTSCREACLQGWTE	95	3169.571	95	2413.841	92.5	3915.696	95	7163.639	95	1484.786	95	6886.915	95	7401.502
RTSCREACLQGWTEA	95	2713.518	94.73	2085.01	92.32	3573.882	94	6378.774	95	1570.005	95	6021.776	95	6621.812

Supplementary Table 12. Association analysis of *HLA-II* alleles with CALRmut+ MPNs *versus* JAK2 V617F+ MPNs in the Bulgarian population.

allele	p.value	p.adjusted	estimate	std.error	conf.low	conf.high	statistic	Ntotal	Ntotal.percent	N(disease=0)	N(disease=0).percent	N(disease=1)	N(disease=1).percent
DRB1*16:01	0.004324	0.203214	3.104835	0.397039	1.413303	6.764472	2.853526	40	10.00%	25	7.91%	15	17.86%
DQB1*05:02	0.009721	0.456895	2.447096	0.346111	1.236847	4.849743	2.585592	57	14.25%	38	12.03%	19	22.62%
DQA1*01:02	0.042641	1	1.83454	0.299324	1.018257	3.320215	2.027213	89	22.25%	64	20.25%	25	29.76%
DQA1*01:03	0.047066	1	0.284584	0.632887	0.065717	0.855573	-1.98571	37	9.25%	34	10.76%	3	3.57%
DRB1*08:01	0.053242	1	4.688841	0.799392	0.964687	25.19443	1.932952	7	1.75%	3	0.95%	4	4.76%
DPB1*04:01	0.109149	1	0.645906	0.272842	0.371644	1.088744	-1.60203	147	36.75%	122	38.61%	25	29.76%
DRB1*13:01	0.122022	1	0.198691	1.04505	0.010856	1.016146	-1.54634	20	5.00%	19	6.01%	1	1.19%
DQB1*05:01	0.149366	1	0.471886	0.520899	0.15163	1.215148	-1.44178	39	9.75%	34	10.76%	5	5.95%
DPB1*10:01	0.165639	1	2.2825	0.595283	0.659464	7.13929	1.386352	15	3.75%	10	3.16%	5	5.95%
DPB1*04:02	0.254856	1	1.412708	0.303441	0.766555	2.543262	1.138634	71	17.75%	52	16.46%	19	22.62%
DPB1*01:01	0.26611	1	0.422716	0.774285	0.064825	1.581647	-1.11207	19	4.75%	17	5.38%	2	2.38%
DRB1*14:04	0.29504	1	2.670838	0.938176	0.339157	16.91367	1.04713	5	1.25%	3	0.95%	2	2.38%
DPB1*05:01	0.311707	1	2.11395	0.739941	0.424591	8.592075	1.011646	9	2.25%	6	1.90%	3	3.57%
DRB1*15:02	0.356341	1	0.481697	0.791926	0.072168	1.883257	-0.92236	15	3.75%	13	4.11%	2	2.38%
DQB1*06:03	0.359704	1	0.571555	0.610739	0.13587	1.634845	-0.91593	25	6.25%	22	6.96%	3	3.57%
DRB1*13:02	0.379343	1	0.388453	1.075616	0.020619	2.198792	-0.87911	10	2.50%	9	2.85%	1	1.19%
DRB1*15:01	0.438211	1	1.410225	0.443422	0.552332	3.264418	0.775218	26	6.50%	19	6.01%	7	8.33%
DQA1*01:04	0.439929	1	0.614379	0.63076	0.142037	1.847679	-0.77231	20	5.00%	17	5.38%	3	3.57%

DQB1*03:02	0.441903	1	1.387794	0.426167	0.569663	3.128443	0.768984	28	7.00%	20	6.33%	8	9.52%
DRB1*04:05	0.483475	1	1.982571	0.976695	0.23775	13.53418	0.700725	5	1.25%	3	0.95%	2	2.38%
DQA1*05:05	0.48512	1	0.800448	0.318846	0.413348	1.459086	-0.69809	81	20.25%	67	21.20%	14	16.67%
DQA1*02:01	0.488117	1	0.69416	0.526539	0.221474	1.816888	-0.69331	31	7.75%	26	8.23%	5	5.95%
DRB1*11:01	0.617141	1	0.776194	0.506801	0.254717	1.93748	-0.49991	32	8.00%	27	8.54%	5	5.95%
DQB1*06:01	0.630009	1	0.67431	0.818049	0.097746	2.836557	-0.48171	11	2.75%	9	2.85%	2	2.38%
DQB1*06:02	0.646591	1	1.322692	0.609962	0.351964	4.089593	0.458502	16	4.00%	12	3.80%	4	4.76%
DRB1*03:01	0.680054	1	1.166931	0.374349	0.537813	2.373732	0.412389	46	11.50%	35	11.08%	11	13.10%
DRB1*07:01	0.716232	1	0.840642	0.477549	0.300494	2.020758	-0.3635	33	8.25%	27	8.54%	6	7.14%
DQA1*01:01	0.732913	1	0.833233	0.534623	0.262529	2.223566	-0.34125	27	6.75%	22	6.96%	5	5.95%
DQB1*02:01	0.745558	1	1.123221	0.358088	0.532994	2.210092	0.324502	47	11.75%	36	11.39%	11	13.10%
DQB1*03:01	0.760204	1	0.911192	0.30471	0.488775	1.628583	-0.30521	89	22.25%	72	22.78%	17	20.24%
DQB1*06:04	0.767978	1	0.722547	1.101524	0.03743	4.486999	-0.29502	7	1.75%	6	1.90%	1	1.19%
DQA1*03:03	0.798402	1	1.256941	0.895327	0.165869	6.614573	0.255416	7	1.75%	5	1.58%	2	2.38%
DPB1*02:01	0.800248	1	1.085732	0.325082	0.557354	2.016139	0.253026	67	16.75%	52	16.46%	15	17.86%
DQB1*02:02	0.821213	1	0.884898	0.541109	0.275896	2.396239	-0.22599	25	6.25%	20	6.33%	5	5.95%
DQB1*05:03	0.833235	1	0.868406	0.670117	0.190959	2.890693	-0.21055	16	4.00%	13	4.11%	3	3.57%
DRB1*01:01	0.849585	1	1.10961	0.548432	0.342598	3.067202	0.189648	23	5.75%	18	5.70%	5	5.95%
DPB1*13:01	0.896218	1	0.899413	0.81273	0.131865	3.78359	-0.13044	10	2.50%	8	2.53%	2	2.38%
DQA1*03:01	0.930123	1	1.03932	0.439799	0.406415	2.359601	0.087691	31	7.75%	24	7.59%	7	8.33%
DQA1*05:01	0.936523	1	1.029457	0.364529	0.481674	2.045025	0.07964	50	12.50%	39	12.34%	11	13.10%
DRB1*16:02	0.969011	1	1.032633	0.826587	0.148851	4.52371	0.038849	9	2.25%	7	2.22%	2	2.38%

DQA1*01:05	0.985447	1	2.15E-07	841.6181	NA	1.79E+24	-0.01824	8	2.00%	8	2.53%	0	0.00%
DRB1*10:01	0.985447	1	2.15E-07	841.6181	NA	1.79E+24	-0.01824	8	2.00%	8	2.53%	0	0.00%
DRB1*01:02	0.988462	1	2.04E-07	1065.422	NA	2.05E+41	-0.01446	5	1.25%	5	1.58%	0	0.00%
DPB1*17:01	0.988544	1	2.11E-07	1070.577	NA	3.78E+41	-0.01436	5	1.25%	5	1.58%	0	0.00%
DQB1*03:03	0.988715	1	2.71E-07	1069.106	NA	3.64E+41	-0.01414	5	1.25%	5	1.58%	0	0.00%
DRB1*14:54	0.989546	1	8.37E-08	1243.803	NA	3.07E+34	-0.0131	10	2.50%	10	3.16%	0	0.00%
DRB1*04:03	0.990072	1	8.21E-08	1311.21	NA	5.12E+38	-0.01244	9	2.25%	9	2.85%	0	0.00%

Supplementary Table 13. Pseudobulk analysis of *HLA-I* and *HLA-II* pathway genes in HSCs from five ET cases.

	baseMean	log2FoldChange	lfcSE	stat	pvalue	padj
CAPZB	423.448	0.095018	0.163631	0.580683	0.561454	0.992284
KIF2C	4.34972	-0.81581	0.878791	-0.92833	0.353237	0.992284
CAPZA1	235.9856	0.035339	0.411583	0.08586	0.931578	0.992284
CTSS	215.7457	0.100075	0.198063	0.505269	0.61337	0.992284
FCER1G	44.08501	0.003418	0.477787	0.007153	0.994293	0.994293
HSPA6	0.037109	1.099131	3.149795	0.348953	0.727124	0.992284
FCGR2B	5.288403	-0.81744	1.205058	-0.67834	0.497558	0.992284
KIFAP3	53.51143	0.033507	0.372669	0.089912	0.928357	0.992284
CTSE	0.14072	-0.32358	3.088314	-0.10478	0.916554	0.992284
ARF1	518.5182	0.085738	0.315018	0.27217	0.785491	0.992284
KIF3C	6.221988	0.284497	0.525478	0.541405	0.588228	0.992284
DCTN1	30.33903	-0.13153	0.285264	-0.46108	0.644739	0.992284
ACTR1B	72.36459	-0.01528	0.223289	-0.06841	0.945457	0.992284
DYNC1I2	117.072	0.263272	0.235383	1.118485	0.26336	0.992284
PIKFYVE	67.79397	0.405094	0.293127	1.381974	0.16698	0.992284
AP1S3	3.466018	-0.37664	0.800464	-0.47053	0.637975	0.992284
SEC13	49.32976	-0.13317	0.413071	-0.32239	0.747157	0.992284
DYNC1LI1	115.7101	0.092199	0.220132	0.418835	0.675337	0.992284
KIF15	4.312099	-0.56002	0.965422	-0.58007	0.561864	0.992284
RAB7A	277.947	0.056243	0.472018	0.119154	0.905154	0.992284
AP2M1	399.6964	-0.04617	0.201423	-0.22922	0.818702	0.992284
SEC31A	130.0647	0.143208	0.211611	0.676754	0.498562	0.992284
CENPE	8.55037	0.022625	0.534358	0.042341	0.966227	0.992284
SEC24B	23.60041	-0.17229	0.353894	-0.48683	0.626377	0.992284
SEC24D	18.80612	0.149892	0.339587	0.441395	0.658927	0.992284
KIF2A	207.638	0.161287	0.393391	0.409992	0.681812	0.992284
KIF3A	20.88279	0.070204	0.449433	0.156206	0.875871	0.992284
HSPA4	104.1967	0.145874	0.259431	0.562284	0.573922	0.992284

SAR1B	55.47064	0.079013	0.251681	0.313941	0.753566	0.992284
SEC24A	12.09511	-0.54723	0.59709	-0.9165	0.359404	0.992284
CD74	4077.052	0.081592	0.431479	0.189098	0.850016	0.992284
DCTN4	53.48315	0.317781	0.428759	0.741164	0.458594	0.992284
KIF4B	0.036757	1.099131	3.149795	0.348953	0.727124	0.992284
CANX	358.8396	-0.06058	0.15659	-0.3869	0.698834	0.992284
HLA-F	74.52918	0.196572	0.488292	0.40257	0.687265	0.992284
HLA-G	3.230859	1.816747	0.776264	2.340373	0.019264	0.992284
HLA-A	2234.187	0.082499	0.340041	0.242614	0.808305	0.992284
HLA-E	1522.653	0.281419	0.235573	1.194614	0.232238	0.992284
HLA-C	1288.865	0.110412	0.296887	0.371897	0.709969	0.992284
HLA-B	3034.767	0.155694	0.300616	0.517917	0.604516	0.992284
HSPA1L	1.952329	-0.50454	0.943963	-0.53449	0.593002	0.992284
HSPA1A	135.005	0.25431	0.497705	0.510966	0.609375	0.992284
HSPA1B	19.87954	0.286914	0.649306	0.441878	0.658577	0.992284
HLA-DRA	4489.866	0.13745	0.201934	0.680667	0.496082	0.992284
HLA-DRB5	306.4554	0.107231	1.36424	0.078601	0.93735	0.992284
HLA-DRB1	1443.337	0.175487	0.51408	0.341361	0.732832	0.992284
HLA-DQA1	274.0971	0.224703	0.302911	0.741809	0.458203	0.992284
HLA-DQB1	407.7632	0.229033	0.393468	0.582088	0.560507	0.992284
HLA-DQA2	107.3975	0.057173	1.988356	0.028754	0.977061	0.992284
HLA-DQB2	0.804465	0.33042	1.443999	0.228823	0.819007	0.992284
HLA-DOB	0.800327	-0.90489	1.39227	-0.64994	0.515733	0.992284
TAP2	15.74432	0.276606	2.902324	0.095305	0.924072	0.992284
TAP1	82.23449	0.255831	0.388479	0.658545	0.510188	0.992284
HLA-DMB	101.9939	0.007647	0.276842	0.027621	0.977964	0.992284
HLA-DMA	721.6301	0.166886	0.192839	0.865416	0.386811	0.992284
HLA-DOA	44.30495	0.339324	0.360406	0.941505	0.346446	0.992284
HLA-DPA1	1887.239	0.097218	0.230315	0.422108	0.672946	0.992284
HLA-DPB1	2182.429	0.15329	0.246011	0.623102	0.533218	0.992284
TAPBP	266.5664	0.064134	0.361953	0.177188	0.859361	0.992284
TREM2	0	NA	NA	NA	NA	NA

DYNC1I1	1.721491	-0.21004	0.97362	-0.21573	0.8292	0.992284
AP1S1	62.27458	0.051065	0.447429	0.114129	0.909135	0.992284
CAPZA2	379.6484	0.210246	0.277957	0.756399	0.44941	0.992284
AP1S2	213.1326	0.133831	0.294224	0.45486	0.64921	0.992284
KIF4A	2.266372	-0.72888	1.059277	-0.6881	0.491392	0.992284
DCTN6	147.969	-0.04252	0.582941	-0.07294	0.941853	0.992284
SH3GL2	0	NA	NA	NA	NA	NA
DCTN3	238.2616	0.147043	0.177386	0.828948	0.407134	0.992284
CLTA	601.0825	0.01671	0.211501	0.079008	0.937026	0.992284
CTSL	0.420578	0.359709	1.864906	0.192883	0.84705	0.992284
CTSV	0	NA	NA	NA	NA	NA
HSPA5	342.4899	-0.52355	0.269333	-1.94388	0.05191	0.992284
AP2A2	38.47079	-0.14468	0.509521	-0.28394	0.776454	0.992284
CTSD	214.2686	0.0473	0.222446	0.212634	0.831612	0.992284
KIF18A	7.782419	-0.3176	0.446521	-0.71128	0.476912	0.992284
TRAF6	15.90894	-0.08309	0.355984	-0.23341	0.815445	0.992284
KLC2	4.898038	-0.41841	0.699379	-0.59826	0.549666	0.992284
CTSF	112.9302	0.346114	0.340361	1.016904	0.309199	0.992284
SPTBN2	2.779703	-0.85612	0.833858	-1.02669	0.304565	0.992284
HSPA8	720.4667	-0.18666	0.482893	-0.38655	0.699092	0.992284
SEC24C	20.77433	0.132017	0.327286	0.40337	0.686676	0.992284
KIF11	9.592776	-0.36907	0.472482	-0.78114	0.434722	0.992284
ACTR1A	81.23577	-0.13336	0.360268	-0.37018	0.711248	0.992284
LAG3	20.05148	0.164724	0.394454	0.4176	0.67624	0.992284
CAPZA3	0	NA	NA	NA	NA	NA
RACGAP1	6.163613	-0.61622	0.657202	-0.93764	0.34843	0.992284
DCTN2	253.0104	0.068389	0.190949	0.358151	0.72023	0.992284
KIF5A	5.03731	0.840213	0.567524	1.480489	0.138743	0.992284
DYNLL1	774.9421	-0.03302	0.791735	-0.0417	0.966736	0.992284
PSME1	845.466	0.004794	0.214987	0.022299	0.982209	0.992284
PSME2	390.9609	-0.19427	0.261021	-0.74426	0.456719	0.992284
SEC23A	47.77436	-0.13746	0.301464	-0.45596	0.648419	0.992284

ACTR10	145.8896	0.143764	0.236345	0.60828	0.543002	0.992284
HSPA2	1.0264	-0.12828	1.065818	-0.12036	0.904201	0.992284
LGMN	0.653724	-1.44942	1.30787	-1.10823	0.267764	0.992284
DYNC1H1	129.8504	0.314121	0.343112	0.915507	0.359926	0.992284
KLC1	39.94403	-0.12779	0.256953	-0.49732	0.618964	0.992284
KIF26A	0.160437	0.505624	2.657908	0.190234	0.849126	0.992284
THBS1	2.140454	0.663092	1.124205	0.589832	0.555303	0.992284
PDIA3	355.9247	-0.17165	0.301123	-0.57005	0.568645	0.992284
B2M	11986.11	0.072098	0.212146	0.33985	0.733969	0.992284
KIF23	3.776172	-0.68718	0.864281	-0.79508	0.426565	0.992284
CIITA	24.52417	-0.43657	0.450703	-0.96865	0.332718	0.992284
DCTN5	40.5256	0.27075	0.434033	0.6238	0.532759	0.992284
KIF22	57.51805	-0.01929	0.296084	-0.06513	0.948067	0.992284
PYCARD	377.8068	0.095	0.307611	0.308833	0.757449	0.992284
DYNC1LI2	66.90095	0.065272	0.28647	0.227849	0.819763	0.992284
AP1G1	43.52534	-0.00768	0.380074	-0.02021	0.983875	0.992284
RILP	51.4378	0.110362	0.36318	0.303876	0.761223	0.992284
AP2B1	93.49147	0.030828	0.255893	0.120473	0.904108	0.992284
PSME3	36.03191	-0.12362	0.49857	-0.24795	0.80417	0.992284
KIF2B	0	NA	NA	NA	NA	NA
DYNLL2	64.97763	-0.03336	0.551553	-0.06048	0.951776	0.992284
CLTC	66.64216	-0.07473	0.290401	-0.25734	0.796914	0.992284
OSBPL1A	81.41769	0.140481	0.454385	0.309166	0.757195	0.992284
KIF3B	10.37058	-0.23726	0.484484	-0.48971	0.624337	0.992284
AP1M2	0	NA	NA	NA	NA	NA
DNM2	71.43428	0.176351	0.381298	0.462502	0.643722	0.992284
CALR	1234.237	-0.43783	0.423749	-1.03323	0.301498	0.992284
AP1M1	40.19376	0.222153	0.332174	0.668784	0.503633	0.992284
IFI30	0.025991	1.187506	3.152058	0.37674	0.706367	0.992284
AP2S1	275.7714	-0.1504	0.267978	-0.56123	0.574639	0.992284
AP2A1	35.40465	-0.04755	0.289161	-0.16444	0.869382	0.992284
AP1B1	49.82374	-0.05673	0.223655	-0.25363	0.799783	0.992284

Supplementary Table 14. Pseudobulk analysis of *HLA-I* and *HLA-II* pathway genes in CMPs from five ET cases.

	baseMean	log2FoldChange	lfcSE	stat	pvalue	padj
CAPZB	7.098171	0.283922	0.622697	0.455955	0.648422	0.987107
KIF2C	0	NA	NA	NA	NA	NA
CAPZA1	5.363836	0.051112	0.756611	0.067554	0.946141	0.987107
CTSS	4.803735	0.394315	0.752222	0.5242	0.600139	0.987107
FCER1G	2.201474	1.167699	1.098777	1.062726	0.287906	0.987107
HSPA6	0	NA	NA	NA	NA	NA
FCGR2B	0.032708	3.068652	3.347389	0.91673	0.359284	0.987107
KIFAP3	0.70085	-0.73495	1.663922	-0.4417	0.658707	0.987107
CTSE	0	NA	NA	NA	NA	NA
ARF1	9.24233	-0.40484	0.580863	-0.69697	0.485823	0.987107
KIF3C	0.025578	3.068652	3.347389	0.91673	0.359284	0.987107
DCTN1	0.970067	0.674538	1.445586	0.46662	0.640772	0.987107
ACTR1B	0.887204	0.785152	1.457639	0.538646	0.590131	0.987107
DYNC1I2	2.037701	-0.39019	1.051715	-0.371	0.710635	0.987107
PIKFYVE	0.87968	-0.2	1.465356	-0.13649	0.891437	0.987107
AP1S3	0.151977	2.875045	3.328461	0.863776	0.387711	0.987107
SEC13	1.571337	0.093879	1.183914	0.079296	0.936798	0.987107
DYNC1LI1	1.724809	0.702022	1.122097	0.625634	0.531555	0.987107
KIF15	0.067434	1.926532	3.311059	0.581848	0.560669	0.987107
RAB7A	4.589333	0.155443	0.786159	0.197725	0.84326	0.987107
AP2M1	7.868515	0.080761	0.593993	0.135963	0.891851	0.987107
SEC31A	4.446191	0.943333	0.786575	1.199293	0.230414	0.987107
CENPE	0.016277	3.068652	3.347389	0.91673	0.359284	0.987107
SEC24B	0.407585	-0.65808	2.066286	-0.31849	0.750116	0.987107
SEC24D	1.385633	1.330418	1.313881	1.012586	0.311258	0.987107
KIF2A	4.809715	0.599411	0.822255	0.728984	0.466011	0.987107
KIF3A	0.444207	2.011807	2.145727	0.937588	0.348456	0.987107
HSPA4	1.555717	-1.08328	1.28169	-0.8452	0.397999	0.987107
SAR1B	0.935268	-0.90348	1.487139	-0.60753	0.543499	0.987107

SEC24A	0.311123	1.861355	2.422428	0.768384	0.442259	0.987107
CD74	40.92307	0.130638	0.362153	0.360727	0.718304	0.987107
DCTN4	0.601353	0.728015	1.782181	0.408496	0.682909	0.987107
KIF4B	0	NA	NA	NA	NA	NA
CANX	18.0707	0.320599	0.452715	0.708171	0.478839	0.987107
HLA-F	2.095127	1.0045	1.162852	0.863825	0.387684	0.987107
HLA-G	0	NA	NA	NA	NA	NA
HLA-A	49.32175	0.245022	0.303126	0.808318	0.418908	0.987107
HLA-E	17.8684	-0.15035	0.512265	-0.2935	0.769143	0.987107
HLA-C	19.56732	-0.54247	0.466188	-1.16363	0.244573	0.987107
HLA-B	61.00178	-0.04001	0.322011	-0.12426	0.901111	0.987107
HSPA1L	0	NA	NA	NA	NA	NA
HSPA1A	2.42059	0.0623	1.08693	0.057317	0.954292	0.987107
HSPA1B	0.498165	2.317072	1.972342	1.174782	0.240082	0.987107
HLA-DRA	42.01908	-0.18408	0.360885	-0.51008	0.609992	0.987107
HLA-DRB5	3.784349	0.153906	1.705938	0.090218	0.928114	0.987107
HLA-DRB1	17.87701	0.144928	0.478264	0.303029	0.761868	0.987107
HLA-DQA1	1.760359	-0.21798	1.1483	-0.18983	0.849442	0.987107
HLA-DQB1	1.799285	-2.2573	1.302481	-1.73308	0.083082	0.987107
HLA-DQA2	0.795224	-0.30029	1.952423	-0.1538	0.877766	0.987107
HLA-DQB2	0	NA	NA	NA	NA	NA
HLA-DOB	0.032555	2.576532	3.337175	0.77207	0.440073	0.987107
TAP2	0.051156	2.246382	3.328126	0.674969	0.499696	0.987107
TAP1	0.696559	-0.0371	1.792864	-0.02069	0.983489	0.992946
HLA-DMB	0.789289	0.399704	1.515632	0.263721	0.791995	0.987107
HLA-DMA	8.905736	-0.23185	0.597698	-0.38791	0.698082	0.987107
HLA-DOA	0.291956	0.931715	2.266389	0.411101	0.680999	0.987107
HLA-DPA1	15.05188	-0.56165	0.516491	-1.08743	0.276848	0.987107
HLA-DPB1	20.13633	-0.00033	0.439051	-0.00075	0.999405	0.999405
TAPBP	4.034888	0.350973	0.804525	0.436249	0.662656	0.987107
TREM2	0	NA	NA	NA	NA	NA
DYNC1I1	0	NA	NA	NA	NA	NA

AP1S1	1.478156	0.609004	1.200828	0.507153	0.612047	0.987107
CAPZA2	3.801366	0.030288	0.923275	0.032804	0.97383	0.99274
AP1S2	2.968128	-0.40461	1.005493	-0.4024	0.687387	0.987107
KIF4A	0.1357	3.51096	3.341668	1.050661	0.293414	0.987107
DCTN6	2.626599	0.224975	1.017161	0.221179	0.824953	0.987107
SH3GL2	0	NA	NA	NA	NA	NA
DCTN3	5.693813	0.686669	0.681085	1.008199	0.313359	0.987107
CLTA	11.45031	-0.03972	0.500018	-0.07945	0.936677	0.987107
CTSL	0	NA	NA	NA	NA	NA
CTSV	0	NA	NA	NA	NA	NA
HSPA5	15.0161	0.116359	0.470151	0.247493	0.804527	0.987107
AP2A2	0.744432	0.648494	1.655202	0.391791	0.695213	0.987107
CTSD	4.273288	-0.48604	0.839831	-0.57873	0.562768	0.987107
KIF18A	0.693831	2.640749	1.774155	1.488455	0.136631	0.987107
TRAF6	0.388043	1.982839	2.118445	0.935988	0.349279	0.987107
KLC2	0.016277	3.068652	3.347389	0.91673	0.359284	0.987107
CTSF	0.383071	1.338166	2.227863	0.60065	0.548073	0.987107
SPTBN2	0.316832	1.762143	2.376961	0.741343	0.458485	0.987107
HSPA8	14.36275	-0.32457	0.562212	-0.57731	0.563729	0.987107
SEC24C	0.355646	0.478766	2.11561	0.226302	0.820967	0.987107
KIF11	0.396628	2.330443	2.245943	1.037623	0.299445	0.987107
ACTR1A	2.204773	0.787817	1.137751	0.692433	0.488665	0.987107
LAG3	0.016277	3.068652	3.347389	0.91673	0.359284	0.987107
CAPZA3	0	NA	NA	NA	NA	NA
RACGAP1	0.265537	2.982536	2.82015	1.057581	0.290247	0.987107
DCTN2	3.017213	-0.06772	0.903561	-0.07495	0.940253	0.987107
KIF5A	0	NA	NA	NA	NA	NA
DYNLL1	21.98354	-0.10575	0.409209	-0.25841	0.796087	0.987107
PSME1	15.36147	-0.17693	0.512455	-0.34526	0.729899	0.987107
PSME2	8.511314	-0.18484	0.616516	-0.29981	0.764324	0.987107
SEC23A	2.250421	0.899147	1.041555	0.863273	0.387987	0.987107
ACTR10	1.993763	0.162256	1.038416	0.156253	0.875833	0.987107

HSPA2	0	NA	NA	NA	NA	NA
LGMN	0.104259	4.232295	3.341668	1.266522	0.205326	0.987107
DYNC1H1	4.437452	0.476642	0.766127	0.622145	0.533847	0.987107
KLC1	0.298569	0.865125	2.397194	0.36089	0.718181	0.987107
KIF26A	0	NA	NA	NA	NA	NA
THBS1	1.013195	3.797725	3.336409	1.138267	0.255009	0.987107
PDIA3	11.8235	-0.05357	0.56948	-0.09407	0.925057	0.987107
B2M	221.6379	-0.01505	0.241462	-0.06232	0.950306	0.987107
KIF23	0.032708	3.068652	3.347389	0.91673	0.359284	0.987107
CIITA	0.974725	1.286045	1.547282	0.831164	0.405881	0.987107
DCTN5	0.440161	1.362413	2.277252	0.598271	0.549659	0.987107
KIF22	1.959768	0.930887	1.050975	0.885736	0.37576	0.987107
PYCARD	12.0197	-0.06601	0.487915	-0.13528	0.89239	0.987107
DYNC1LI2	1.5258	0.476205	1.202231	0.396101	0.692031	0.987107
AP1G1	0.565424	-0.37	1.759256	-0.21031	0.833422	0.987107
RILP	0.873508	0.587813	1.540506	0.381571	0.702779	0.987107
AP2B1	2.285126	0.387287	1.060913	0.365051	0.715073	0.987107
PSME3	1.125851	0.529109	1.379203	0.383633	0.70125	0.987107
KIF2B	0	NA	NA	NA	NA	NA
DYNLL2	0.971691	0.908605	1.512891	0.600575	0.548123	0.987107
CLTC	2.791794	0.332643	0.907837	0.366413	0.714057	0.987107
OSBPL1A	1.901485	0.506822	1.071518	0.472994	0.636217	0.987107
KIF3B	0.111713	1.212447	3.263854	0.371477	0.710282	0.987107
AP1M2	0	NA	NA	NA	NA	NA
DNM2	1.310362	-0.53039	1.301068	-0.40766	0.683525	0.987107
CALR	47.74317	-0.65315	0.37448	-1.74414	0.081135	0.987107
AP1M1	1.47782	1.567927	1.475481	1.062655	0.287938	0.987107
IFI30	0	NA	NA	NA	NA	NA
AP2S1	6.980708	-0.24443	0.620913	-0.39366	0.69383	0.987107
AP2A1	0.342169	-0.40647	2.232317	-0.18209	0.855515	0.987107
AP1B1	1.441595	-0.06574	1.275731	-0.05153	0.958904	0.987107

Supplementary Table 15. Pseudobulk analysis of *HLA-I* and *HLA-II* pathway genes in MEPs from five ET cases.

	baseMean	log2FoldChange	lfcSE	stat	pvalue	padj
CAPZB	811.9683	0.034417	0.141631	0.243008	0.808	0.988825
KIF2C	26.05304	-1.4884	0.724492	-2.05441	0.039937	0.966463
CAPZA1	576.6797	0.095518	0.375508	0.254371	0.799209	0.988825
CTSS	372.7394	0.237235	0.150856	1.572599	0.115812	0.988825
FCER1G	123.8146	-0.05682	0.439778	-0.1292	0.897198	0.988825
HSPA6	1.705371	1.218706	1.056848	1.153152	0.248848	0.988825
FCGR2B	0.557783	0.121078	1.456498	0.083129	0.933749	0.988825
KIFAP3	282.8838	0.382794	0.360077	1.063089	0.287742	0.988825
CTSE	0.186183	0.119168	2.011622	0.05924	0.952761	0.988825
ARF1	1202.92	0.105989	0.194335	0.54539	0.585485	0.988825
KIF3C	21.1961	0.25869	0.444856	0.581515	0.560893	0.988825
DCTN1	107.9738	0.138963	0.165412	0.8401	0.400852	0.988825
ACTR1B	145.2475	0.073089	0.222945	0.327835	0.743036	0.988825
DYNC1I2	289.5899	0.108015	0.188256	0.573766	0.566126	0.988825
PIKFYVE	126.8274	0.26713	0.277129	0.963917	0.335088	0.988825
AP1S3	4.167195	-0.31337	0.767013	-0.40857	0.682859	0.988825
SEC13	158.1496	-0.27511	0.384725	-0.71509	0.474551	0.988825
DYNC1LI1	269.9362	-0.04777	0.155421	-0.30734	0.758585	0.988825
KIF15	31.1745	-1.63753	0.530133	-3.08891	0.002009	0.152094
RAB7A	634.0579	-0.07361	0.455899	-0.16147	0.871724	0.988825
AP2M1	1195.822	0.068566	0.177453	0.386391	0.699207	0.988825
SEC31A	311.964	0.260143	0.233412	1.114521	0.265056	0.988825
CENPE	45.9271	-0.73496	0.467724	-1.57135	0.1161	0.988825
SEC24B	52.88656	-0.08531	0.303774	-0.28084	0.778833	0.988825
SEC24D	52.92592	0.153036	0.473963	0.322886	0.746782	0.988825
KIF2A	548.1408	-0.0482	0.474826	-0.1015	0.919153	0.988825
KIF3A	51.07994	0.320753	0.58931	0.544286	0.586245	0.988825
HSPA4	297.5453	-0.15858	0.206756	-0.76697	0.443098	0.988825
SAR1B	139.3448	-0.06129	0.276984	-0.22129	0.824864	0.988825

SEC24A	31.43042	0.133327	0.314292	0.424214	0.67141	0.988825
CD74	2790.643	-0.03405	0.437264	-0.07788	0.937927	0.988825
DCTN4	95.41146	0.250876	0.261436	0.959607	0.337253	0.988825
KIF4B	0.16624	-0.54948	3.07078	-0.17894	0.857987	0.988825
CANX	864.4631	-0.11539	0.164246	-0.70256	0.482327	0.988825
HLA-F	94.1821	0.362809	0.790636	0.458883	0.646319	0.988825
HLA-G	3.449744	0.549434	1.005337	0.546517	0.584711	0.988825
HLA-A	3508.894	0.1646	0.357624	0.46026	0.64533	0.988825
HLA-E	1861.988	0.307866	0.310191	0.992504	0.320952	0.988825
HLA-C	2411.047	0.173489	0.324496	0.53464	0.592899	0.988825
HLA-B	5424.809	0.104298	0.292429	0.356662	0.721345	0.988825
HSPA1L	2.496357	-0.65367	0.88389	-0.73954	0.459581	0.988825
HSPA1A	172.7175	0.00377	0.419049	0.008997	0.992822	0.992822
HSPA1B	40.81429	0.106966	0.609704	0.175439	0.860735	0.988825
HLA-DRA	3938.201	0.040011	0.238667	0.167643	0.866864	0.988825
HLA-DRB5	343.2015	-0.09411	1.304556	-0.07214	0.942488	0.988825
HLA-DRB1	1343.885	0.017805	0.497617	0.03578	0.971458	0.988825
HLA-DQA1	167.0825	0.100021	0.265354	0.376934	0.706223	0.988825
HLA-DQB1	197.683	0.017786	0.515584	0.034498	0.97248	0.988825
HLA-DQA2	79.3449	-0.02294	2.000848	-0.01146	0.990854	0.992822
HLA-DQB2	0.280173	0.710148	1.96718	0.360998	0.718101	0.988825
HLA-DOB	4.986122	-0.15633	1.13925	-0.13722	0.890858	0.988825
TAP2	26.85668	-0.15591	2.898351	-0.05379	0.957101	0.988825
TAP1	181.4744	0.215144	0.329121	0.653694	0.513309	0.988825
HLA-DMB	61.98056	0.398767	0.361713	1.102442	0.27027	0.988825
HLA-DMA	934.9557	0.049756	0.193715	0.256852	0.797293	0.988825
HLA-DOA	17.3017	0.10502	0.450828	0.232948	0.815801	0.988825
HLA-DPA1	1422.977	0.015697	0.278604	0.056342	0.955069	0.988825
HLA-DPB1	1627.107	0.034813	0.306079	0.113738	0.909446	0.988825
TAPBP	388.7285	0.074047	0.278034	0.266321	0.789992	0.988825
TREM2	0	NA	NA	NA	NA	NA
DYNC1I1	3.369577	-0.97212	0.835082	-1.1641	0.244384	0.988825

AP1S1	159.4662	-0.19482	0.591329	-0.32947	0.741804	0.988825
CAPZA2	1028.511	0.062133	0.230248	0.269855	0.787272	0.988825
AP1S2	336.6246	0.078263	0.2828	0.276743	0.781977	0.988825
KIF4A	15.51363	-1.47891	0.489437	-3.02166	0.002514	0.152094
DCTN6	350.9603	0.065673	0.438959	0.14961	0.881072	0.988825
SH3GL2	0.043764	0.811995	3.116575	0.260541	0.794447	0.988825
DCTN3	559.6287	0.216165	0.1613	1.340149	0.180197	0.988825
CLTA	1539.628	0.01222	0.212976	0.057375	0.954246	0.988825
CTSL	25.46232	0.437384	1.203897	0.363307	0.716376	0.988825
CTSV	0.60281	1.540511	1.422955	1.082614	0.27898	0.988825
HSPA5	845.621	-0.47483	0.283141	-1.67699	0.093544	0.988825
AP2A2	71.98104	0.166639	0.340346	0.489618	0.624404	0.988825
CTSD	286.9602	-0.06479	0.230716	-0.28082	0.77885	0.988825
KIF18A	30.4026	-0.78039	0.339911	-2.29587	0.021683	0.655916
TRAF6	36.18724	0.013622	0.350748	0.038838	0.96902	0.988825
KLC2	8.980641	0.468036	0.554121	0.844646	0.398309	0.988825
CTSF	130.1718	0.447858	0.244917	1.828612	0.067458	0.988825
SPTBN2	50.24012	0.337103	0.380715	0.885445	0.375917	0.988825
HSPA8	2599.266	-0.28977	0.472925	-0.61271	0.540067	0.988825
SEC24C	54.66376	-0.21768	0.221897	-0.98102	0.326585	0.988825
KIF11	49.98723	-0.82201	0.32038	-2.56572	0.010296	0.41528
ACTR1A	202.8012	0.059117	0.376042	0.157208	0.875081	0.988825
LAG3	3.621259	-0.05362	0.984064	-0.05449	0.956548	0.988825
CAPZA3	0	NA	NA	NA	NA	NA
RACGAP1	42.13749	-0.55817	0.496318	-1.12461	0.260753	0.988825
DCTN2	590.0174	0.047976	0.15867	0.302362	0.762376	0.988825
KIF5A	6.909263	0.230769	0.705911	0.32691	0.743736	0.988825
DYNLL1	1982.236	-0.0854	0.735248	-0.11616	0.907529	0.988825
PSME1	1457.847	0.010275	0.156977	0.065458	0.94781	0.988825
PSME2	888.1235	-0.19219	0.355071	-0.54128	0.588312	0.988825
SEC23A	90.1577	-0.07504	0.180976	-0.41466	0.678394	0.988825
ACTR10	345.2396	0.109052	0.173108	0.629969	0.528715	0.988825

HSPA2	3.74855	-0.62554	1.20528	-0.519	0.603762	0.988825
LGMN	2.827241	0.821682	1.104254	0.744106	0.456812	0.988825
DYNC1H1	326.1082	0.175394	0.285179	0.615032	0.538534	0.988825
KLC1	111.1819	0.297743	0.284488	1.046594	0.295287	0.988825
KIF26A	0.146462	0.412879	3.080674	0.134022	0.893385	0.988825
THBS1	15.95834	-0.33773	1.170769	-0.28847	0.772987	0.988825
PDIA3	1114.231	-0.21958	0.285643	-0.76872	0.442058	0.988825
B2M	21277.58	0.067721	0.211721	0.319861	0.749073	0.988825
KIF23	22.71569	-1.1597	0.593421	-1.95427	0.050669	0.988825
CIITA	23.21261	0.26475	0.41385	0.639724	0.522352	0.988825
DCTN5	77.54658	0.123123	0.315336	0.39045	0.696204	0.988825
KIF22	182.1778	-0.30375	0.317827	-0.9557	0.339222	0.988825
PYCARD	603.7711	0.094619	0.423986	0.223165	0.823407	0.988825
DYNC1LI2	177.7405	0.213045	0.331138	0.643372	0.519983	0.988825
AP1G1	100.5026	0.067477	0.36845	0.183138	0.85469	0.988825
RILP	193.65	0.287972	0.20279	1.420051	0.155593	0.988825
AP2B1	212.4885	0.021547	0.179535	0.120013	0.904473	0.988825
PSME3	121.2566	-0.0977	0.445863	-0.21913	0.826549	0.988825
KIF2B	0	NA	NA	NA	NA	NA
DYNLL2	155.4806	-0.05493	0.425355	-0.12913	0.897252	0.988825
CLTC	219.5876	0.034168	0.299838	0.113954	0.909274	0.988825
OSBPL1A	167.9804	-0.14951	0.420591	-0.35548	0.722229	0.988825
KIF3B	28.03689	0.239959	0.310451	0.772935	0.439561	0.988825
AP1M2	0.023253	1.243033	3.133281	0.396719	0.691574	0.988825
DNM2	186.3077	0.097744	0.323467	0.302177	0.762517	0.988825
CALR	2483.231	-0.57082	0.461026	-1.23816	0.215657	0.988825
AP1M1	97.20365	0.13774	0.261829	0.526068	0.598841	0.988825
IFI30	0.148653	1.357932	3.099762	0.438076	0.661331	0.988825
AP2S1	791.7988	-0.1196	0.184833	-0.64709	0.517576	0.988825
AP2A1	101.4671	0.149382	0.164237	0.909551	0.363059	0.988825
AP1B1	116.0323	-0.09774	0.177996	-0.54913	0.582916	0.988825

Supplementary Table 16. Pseubobulk analysis of *HLA-I* and *HLA-II* pathway genes in HSCs from five MF cases.

	baseMean	log2FoldChange	lfcSE	stat	pvalue	padj
CAPZB	423.448	0.095018	0.163631	0.580683	0.561454	0.992284
KIF2C	4.34972	-0.81581	0.878791	-0.92833	0.353237	0.992284
CAPZA1	235.9856	0.035339	0.411583	0.08586	0.931578	0.992284
CTSS	215.7457	0.100075	0.198063	0.505269	0.61337	0.992284
FCER1G	44.08501	0.003418	0.477787	0.007153	0.994293	0.994293
HSPA6	0.037109	1.099131	3.149795	0.348953	0.727124	0.992284
FCGR2B	5.288403	-0.81744	1.205058	-0.67834	0.497558	0.992284
KIFAP3	53.51143	0.033507	0.372669	0.089912	0.928357	0.992284
CTSE	0.14072	-0.32358	3.088314	-0.10478	0.916554	0.992284
ARF1	518.5182	0.085738	0.315018	0.27217	0.785491	0.992284
KIF3C	6.221988	0.284497	0.525478	0.541405	0.588228	0.992284
DCTN1	30.33903	-0.13153	0.285264	-0.46108	0.644739	0.992284
ACTR1B	72.36459	-0.01528	0.223289	-0.06841	0.945457	0.992284
DYNC1I2	117.072	0.263272	0.235383	1.118485	0.26336	0.992284
PIKFYVE	67.79397	0.405094	0.293127	1.381974	0.16698	0.992284
AP1S3	3.466018	-0.37664	0.800464	-0.47053	0.637975	0.992284
SEC13	49.32976	-0.13317	0.413071	-0.32239	0.747157	0.992284
DYNC1LI1	115.7101	0.092199	0.220132	0.418835	0.675337	0.992284
KIF15	4.312099	-0.56002	0.965422	-0.58007	0.561864	0.992284
RAB7A	277.947	0.056243	0.472018	0.119154	0.905154	0.992284
AP2M1	399.6964	-0.04617	0.201423	-0.22922	0.818702	0.992284
SEC31A	130.0647	0.143208	0.211611	0.676754	0.498562	0.992284
CENPE	8.55037	0.022625	0.534358	0.042341	0.966227	0.992284
SEC24B	23.60041	-0.17229	0.353894	-0.48683	0.626377	0.992284
SEC24D	18.80612	0.149892	0.339587	0.441395	0.658927	0.992284
KIF2A	207.638	0.161287	0.393391	0.409992	0.681812	0.992284
KIF3A	20.88279	0.070204	0.449433	0.156206	0.875871	0.992284
HSPA4	104.1967	0.145874	0.259431	0.562284	0.573922	0.992284
SAR1B	55.47064	0.079013	0.251681	0.313941	0.753566	0.992284

SEC24A	12.09511	-0.54723	0.59709	-0.9165	0.359404	0.992284
CD74	4077.052	0.081592	0.431479	0.189098	0.850016	0.992284
DCTN4	53.48315	0.317781	0.428759	0.741164	0.458594	0.992284
KIF4B	0.036757	1.099131	3.149795	0.348953	0.727124	0.992284
CANX	358.8396	-0.06058	0.15659	-0.3869	0.698834	0.992284
HLA-F	74.52918	0.196572	0.488292	0.40257	0.687265	0.992284
HLA-G	3.230859	1.816747	0.776264	2.340373	0.019264	0.992284
HLA-A	2234.187	0.082499	0.340041	0.242614	0.808305	0.992284
HLA-E	1522.653	0.281419	0.235573	1.194614	0.232238	0.992284
HLA-C	1288.865	0.110412	0.296887	0.371897	0.709969	0.992284
HLA-B	3034.767	0.155694	0.300616	0.517917	0.604516	0.992284
HSPA1L	1.952329	-0.50454	0.943963	-0.53449	0.593002	0.992284
HSPA1A	135.005	0.25431	0.497705	0.510966	0.609375	0.992284
HSPA1B	19.87954	0.286914	0.649306	0.441878	0.658577	0.992284
HLA-DRA	4489.866	0.13745	0.201934	0.680667	0.496082	0.992284
HLA-DRB5	306.4554	0.107231	1.36424	0.078601	0.93735	0.992284
HLA-DRB1	1443.337	0.175487	0.51408	0.341361	0.732832	0.992284
HLA-DQA1	274.0971	0.224703	0.302911	0.741809	0.458203	0.992284
HLA-DQB1	407.7632	0.229033	0.393468	0.582088	0.560507	0.992284
HLA-DQA2	107.3975	0.057173	1.988356	0.028754	0.977061	0.992284
HLA-DQB2	0.804465	0.33042	1.443999	0.228823	0.819007	0.992284
HLA-DOB	0.800327	-0.90489	1.39227	-0.64994	0.515733	0.992284
TAP2	15.74432	0.276606	2.902324	0.095305	0.924072	0.992284
TAP1	82.23449	0.255831	0.388479	0.658545	0.510188	0.992284
HLA-DMB	101.9939	0.007647	0.276842	0.027621	0.977964	0.992284
HLA-DMA	721.6301	0.166886	0.192839	0.865416	0.386811	0.992284
HLA-DOA	44.30495	0.339324	0.360406	0.941505	0.346446	0.992284
HLA-DPA1	1887.239	0.097218	0.230315	0.422108	0.672946	0.992284
HLA-DPB1	2182.429	0.15329	0.246011	0.623102	0.533218	0.992284
TAPBP	266.5664	0.064134	0.361953	0.177188	0.859361	0.992284
TREM2	0	NA	NA	NA	NA	NA
DYNC1I1	1.721491	-0.21004	0.97362	-0.21573	0.8292	0.992284

AP1S1	62.27458	0.051065	0.447429	0.114129	0.909135	0.992284
CAPZA2	379.6484	0.210246	0.277957	0.756399	0.44941	0.992284
AP1S2	213.1326	0.133831	0.294224	0.45486	0.64921	0.992284
KIF4A	2.266372	-0.72888	1.059277	-0.6881	0.491392	0.992284
DCTN6	147.969	-0.04252	0.582941	-0.07294	0.941853	0.992284
SH3GL2	0	NA	NA	NA	NA	NA
DCTN3	238.2616	0.147043	0.177386	0.828948	0.407134	0.992284
CLTA	601.0825	0.01671	0.211501	0.079008	0.937026	0.992284
CTSL	0.420578	0.359709	1.864906	0.192883	0.84705	0.992284
CTSV	0	NA	NA	NA	NA	NA
HSPA5	342.4899	-0.52355	0.269333	-1.94388	0.05191	0.992284
AP2A2	38.47079	-0.14468	0.509521	-0.28394	0.776454	0.992284
CTSD	214.2686	0.0473	0.222446	0.212634	0.831612	0.992284
KIF18A	7.782419	-0.3176	0.446521	-0.71128	0.476912	0.992284
TRAF6	15.90894	-0.08309	0.355984	-0.23341	0.815445	0.992284
KLC2	4.898038	-0.41841	0.699379	-0.59826	0.549666	0.992284
CTSF	112.9302	0.346114	0.340361	1.016904	0.309199	0.992284
SPTBN2	2.779703	-0.85612	0.833858	-1.02669	0.304565	0.992284
HSPA8	720.4667	-0.18666	0.482893	-0.38655	0.699092	0.992284
SEC24C	20.77433	0.132017	0.327286	0.40337	0.686676	0.992284
KIF11	9.592776	-0.36907	0.472482	-0.78114	0.434722	0.992284
ACTR1A	81.23577	-0.13336	0.360268	-0.37018	0.711248	0.992284
LAG3	20.05148	0.164724	0.394454	0.4176	0.67624	0.992284
CAPZA3	0	NA	NA	NA	NA	NA
RACGAP1	6.163613	-0.61622	0.657202	-0.93764	0.34843	0.992284
DCTN2	253.0104	0.068389	0.190949	0.358151	0.72023	0.992284
KIF5A	5.03731	0.840213	0.567524	1.480489	0.138743	0.992284
DYNLL1	774.9421	-0.03302	0.791735	-0.0417	0.966736	0.992284
PSME1	845.466	0.004794	0.214987	0.022299	0.982209	0.992284
PSME2	390.9609	-0.19427	0.261021	-0.74426	0.456719	0.992284
SEC23A	47.77436	-0.13746	0.301464	-0.45596	0.648419	0.992284
ACTR10	145.8896	0.143764	0.236345	0.60828	0.543002	0.992284

HSPA2	1.0264	-0.12828	1.065818	-0.12036	0.904201	0.992284
LGMN	0.653724	-1.44942	1.30787	-1.10823	0.267764	0.992284
DYNC1H1	129.8504	0.314121	0.343112	0.915507	0.359926	0.992284
KLC1	39.94403	-0.12779	0.256953	-0.49732	0.618964	0.992284
KIF26A	0.160437	0.505624	2.657908	0.190234	0.849126	0.992284
THBS1	2.140454	0.663092	1.124205	0.589832	0.555303	0.992284
PDIA3	355.9247	-0.17165	0.301123	-0.57005	0.568645	0.992284
B2M	11986.11	0.072098	0.212146	0.33985	0.733969	0.992284
KIF23	3.776172	-0.68718	0.864281	-0.79508	0.426565	0.992284
CIITA	24.52417	-0.43657	0.450703	-0.96865	0.332718	0.992284
DCTN5	40.5256	0.27075	0.434033	0.6238	0.532759	0.992284
KIF22	57.51805	-0.01929	0.296084	-0.06513	0.948067	0.992284
PYCARD	377.8068	0.095	0.307611	0.308833	0.757449	0.992284
DYNC1LI2	66.90095	0.065272	0.28647	0.227849	0.819763	0.992284
AP1G1	43.52534	-0.00768	0.380074	-0.02021	0.983875	0.992284
RILP	51.4378	0.110362	0.36318	0.303876	0.761223	0.992284
AP2B1	93.49147	0.030828	0.255893	0.120473	0.904108	0.992284
PSME3	36.03191	-0.12362	0.49857	-0.24795	0.80417	0.992284
KIF2B	0	NA	NA	NA	NA	NA
DYNLL2	64.97763	-0.03336	0.551553	-0.06048	0.951776	0.992284
CLTC	66.64216	-0.07473	0.290401	-0.25734	0.796914	0.992284
OSBPL1A	81.41769	0.140481	0.454385	0.309166	0.757195	0.992284
KIF3B	10.37058	-0.23726	0.484484	-0.48971	0.624337	0.992284
AP1M2	0	NA	NA	NA	NA	NA
DNM2	71.43428	0.176351	0.381298	0.462502	0.643722	0.992284
CALR	1234.237	-0.43783	0.423749	-1.03323	0.301498	0.992284
AP1M1	40.19376	0.222153	0.332174	0.668784	0.503633	0.992284
IFI30	0.025991	1.187506	3.152058	0.37674	0.706367	0.992284
AP2S1	275.7714	-0.1504	0.267978	-0.56123	0.574639	0.992284
AP2A1	35.40465	-0.04755	0.289161	-0.16444	0.869382	0.992284
AP1B1	49.82374	-0.05673	0.223655	-0.25363	0.799783	0.992284

Supplementary Table 17. Pseudobulk analysis of *HLA-I* and *HLA-II* pathway genes in CMPs from five MF cases.

	baseMean	log2FoldChange	lfcSE	stat	pvalue	padj
CAPZB	7.098171	0.283922	0.622697	0.455955	0.648422	0.987107
KIF2C	0	NA	NA	NA	NA	NA
CAPZA1	5.363836	0.051112	0.756611	0.067554	0.946141	0.987107
CTSS	4.803735	0.394315	0.752222	0.5242	0.600139	0.987107
FCER1G	2.201474	1.167699	1.098777	1.062726	0.287906	0.987107
HSPA6	0	NA	NA	NA	NA	NA
FCGR2B	0.032708	3.068652	3.347389	0.91673	0.359284	0.987107
KIFAP3	0.70085	-0.73495	1.663922	-0.4417	0.658707	0.987107
CTSE	0	NA	NA	NA	NA	NA
ARF1	9.24233	-0.40484	0.580863	-0.69697	0.485823	0.987107
KIF3C	0.025578	3.068652	3.347389	0.91673	0.359284	0.987107
DCTN1	0.970067	0.674538	1.445586	0.46662	0.640772	0.987107
ACTR1B	0.887204	0.785152	1.457639	0.538646	0.590131	0.987107
DYNC1I2	2.037701	-0.39019	1.051715	-0.371	0.710635	0.987107
PIKFYVE	0.87968	-0.2	1.465356	-0.13649	0.891437	0.987107
AP1S3	0.151977	2.875045	3.328461	0.863776	0.387711	0.987107
SEC13	1.571337	0.093879	1.183914	0.079296	0.936798	0.987107
DYNC1LI1	1.724809	0.702022	1.122097	0.625634	0.531555	0.987107
KIF15	0.067434	1.926532	3.311059	0.581848	0.560669	0.987107
RAB7A	4.589333	0.155443	0.786159	0.197725	0.84326	0.987107
AP2M1	7.868515	0.080761	0.593993	0.135963	0.891851	0.987107
SEC31A	4.446191	0.943333	0.786575	1.199293	0.230414	0.987107
CENPE	0.016277	3.068652	3.347389	0.91673	0.359284	0.987107
SEC24B	0.407585	-0.65808	2.066286	-0.31849	0.750116	0.987107
SEC24D	1.385633	1.330418	1.313881	1.012586	0.311258	0.987107
KIF2A	4.809715	0.599411	0.822255	0.728984	0.466011	0.987107
KIF3A	0.444207	2.011807	2.145727	0.937588	0.348456	0.987107
HSPA4	1.555717	-1.08328	1.28169	-0.8452	0.397999	0.987107
SAR1B	0.935268	-0.90348	1.487139	-0.60753	0.543499	0.987107

SEC24A	0.311123	1.861355	2.422428	0.768384	0.442259	0.987107
CD74	40.92307	0.130638	0.362153	0.360727	0.718304	0.987107
DCTN4	0.601353	0.728015	1.782181	0.408496	0.682909	0.987107
KIF4B	0	NA	NA	NA	NA	NA
CANX	18.0707	0.320599	0.452715	0.708171	0.478839	0.987107
HLA-F	2.095127	1.0045	1.162852	0.863825	0.387684	0.987107
HLA-G	0	NA	NA	NA	NA	NA
HLA-A	49.32175	0.245022	0.303126	0.808318	0.418908	0.987107
HLA-E	17.8684	-0.15035	0.512265	-0.2935	0.769143	0.987107
HLA-C	19.56732	-0.54247	0.466188	-1.16363	0.244573	0.987107
HLA-B	61.00178	-0.04001	0.322011	-0.12426	0.901111	0.987107
HSPA1L	0	NA	NA	NA	NA	NA
HSPA1A	2.42059	0.0623	1.08693	0.057317	0.954292	0.987107
HSPA1B	0.498165	2.317072	1.972342	1.174782	0.240082	0.987107
HLA-DRA	42.01908	-0.18408	0.360885	-0.51008	0.609992	0.987107
HLA-DRB5	3.784349	0.153906	1.705938	0.090218	0.928114	0.987107
HLA-DRB1	17.87701	0.144928	0.478264	0.303029	0.761868	0.987107
HLA-DQA1	1.760359	-0.21798	1.1483	-0.18983	0.849442	0.987107
HLA-DQB1	1.799285	-2.2573	1.302481	-1.73308	0.083082	0.987107
HLA-DQA2	0.795224	-0.30029	1.952423	-0.1538	0.877766	0.987107
HLA-DQB2	0	NA	NA	NA	NA	NA
HLA-DOB	0.032555	2.576532	3.337175	0.77207	0.440073	0.987107
TAP2	0.051156	2.246382	3.328126	0.674969	0.499696	0.987107
TAP1	0.696559	-0.0371	1.792864	-0.02069	0.983489	0.992946
HLA-DMB	0.789289	0.399704	1.515632	0.263721	0.791995	0.987107
HLA-DMA	8.905736	-0.23185	0.597698	-0.38791	0.698082	0.987107
HLA-DOA	0.291956	0.931715	2.266389	0.411101	0.680999	0.987107
HLA-DPA1	15.05188	-0.56165	0.516491	-1.08743	0.276848	0.987107
HLA-DPB1	20.13633	-0.00033	0.439051	-0.00075	0.999405	0.999405
TAPBP	4.034888	0.350973	0.804525	0.436249	0.662656	0.987107
TREM2	0	NA	NA	NA	NA	NA
DYNC1I1	0	NA	NA	NA	NA	NA

AP1S1	1.478156	0.609004	1.200828	0.507153	0.612047	0.987107
CAPZA2	3.801366	0.030288	0.923275	0.032804	0.97383	0.99274
AP1S2	2.968128	-0.40461	1.005493	-0.4024	0.687387	0.987107
KIF4A	0.1357	3.51096	3.341668	1.050661	0.293414	0.987107
DCTN6	2.626599	0.224975	1.017161	0.221179	0.824953	0.987107
SH3GL2	0	NA	NA	NA	NA	NA
DCTN3	5.693813	0.686669	0.681085	1.008199	0.313359	0.987107
CLTA	11.45031	-0.03972	0.500018	-0.07945	0.936677	0.987107
CTSL	0	NA	NA	NA	NA	NA
CTSV	0	NA	NA	NA	NA	NA
HSPA5	15.0161	0.116359	0.470151	0.247493	0.804527	0.987107
AP2A2	0.744432	0.648494	1.655202	0.391791	0.695213	0.987107
CTSD	4.273288	-0.48604	0.839831	-0.57873	0.562768	0.987107
KIF18A	0.693831	2.640749	1.774155	1.488455	0.136631	0.987107
TRAF6	0.388043	1.982839	2.118445	0.935988	0.349279	0.987107
KLC2	0.016277	3.068652	3.347389	0.91673	0.359284	0.987107
CTSF	0.383071	1.338166	2.227863	0.60065	0.548073	0.987107
SPTBN2	0.316832	1.762143	2.376961	0.741343	0.458485	0.987107
HSPA8	14.36275	-0.32457	0.562212	-0.57731	0.563729	0.987107
SEC24C	0.355646	0.478766	2.11561	0.226302	0.820967	0.987107
KIF11	0.396628	2.330443	2.245943	1.037623	0.299445	0.987107
ACTR1A	2.204773	0.787817	1.137751	0.692433	0.488665	0.987107
LAG3	0.016277	3.068652	3.347389	0.91673	0.359284	0.987107
CAPZA3	0	NA	NA	NA	NA	NA
RACGAP1	0.265537	2.982536	2.82015	1.057581	0.290247	0.987107
DCTN2	3.017213	-0.06772	0.903561	-0.07495	0.940253	0.987107
KIF5A	0	NA	NA	NA	NA	NA
DYNLL1	21.98354	-0.10575	0.409209	-0.25841	0.796087	0.987107
PSME1	15.36147	-0.17693	0.512455	-0.34526	0.729899	0.987107
PSME2	8.511314	-0.18484	0.616516	-0.29981	0.764324	0.987107
SEC23A	2.250421	0.899147	1.041555	0.863273	0.387987	0.987107
ACTR10	1.993763	0.162256	1.038416	0.156253	0.875833	0.987107

HSPA2	0	NA	NA	NA	NA	NA
LGMN	0.104259	4.232295	3.341668	1.266522	0.205326	0.987107
DYNC1H1	4.437452	0.476642	0.766127	0.622145	0.533847	0.987107
KLC1	0.298569	0.865125	2.397194	0.36089	0.718181	0.987107
KIF26A	0	NA	NA	NA	NA	NA
THBS1	1.013195	3.797725	3.336409	1.138267	0.255009	0.987107
PDIA3	11.8235	-0.05357	0.56948	-0.09407	0.925057	0.987107
B2M	221.6379	-0.01505	0.241462	-0.06232	0.950306	0.987107
KIF23	0.032708	3.068652	3.347389	0.91673	0.359284	0.987107
CIITA	0.974725	1.286045	1.547282	0.831164	0.405881	0.987107
DCTN5	0.440161	1.362413	2.277252	0.598271	0.549659	0.987107
KIF22	1.959768	0.930887	1.050975	0.885736	0.37576	0.987107
PYCARD	12.0197	-0.06601	0.487915	-0.13528	0.89239	0.987107
DYNC1LI2	1.5258	0.476205	1.202231	0.396101	0.692031	0.987107
AP1G1	0.565424	-0.37	1.759256	-0.21031	0.833422	0.987107
RILP	0.873508	0.587813	1.540506	0.381571	0.702779	0.987107
AP2B1	2.285126	0.387287	1.060913	0.365051	0.715073	0.987107
PSME3	1.125851	0.529109	1.379203	0.383633	0.70125	0.987107
KIF2B	0	NA	NA	NA	NA	NA
DYNLL2	0.971691	0.908605	1.512891	0.600575	0.548123	0.987107
CLTC	2.791794	0.332643	0.907837	0.366413	0.714057	0.987107
OSBPL1A	1.901485	0.506822	1.071518	0.472994	0.636217	0.987107
KIF3B	0.111713	1.212447	3.263854	0.371477	0.710282	0.987107
AP1M2	0	NA	NA	NA	NA	NA
DNM2	1.310362	-0.53039	1.301068	-0.40766	0.683525	0.987107
CALR	47.74317	-0.65315	0.37448	-1.74414	0.081135	0.987107
AP1M1	1.47782	1.567927	1.475481	1.062655	0.287938	0.987107
IFI30	0	NA	NA	NA	NA	NA
AP2S1	6.980708	-0.24443	0.620913	-0.39366	0.69383	0.987107
AP2A1	0.342169	-0.40647	2.232317	-0.18209	0.855515	0.987107
AP1B1	1.441595	-0.06574	1.275731	-0.05153	0.958904	0.987107

Supplementary Table 18. Pseudobulk analysis of *HLA-I* and *HLA-II* pathway genes in MEPs from five MF cases.

	baseMean	log2FoldChange	lfcSE	stat	pvalue	padj
CAPZB	811.9683	0.034417	0.141631	0.243008	0.808	0.988825
KIF2C	26.05304	-1.4884	0.724492	-2.05441	0.039937	0.966463
CAPZA1	576.6797	0.095518	0.375508	0.254371	0.799209	0.988825
CTSS	372.7394	0.237235	0.150856	1.572599	0.115812	0.988825
FCER1G	123.8146	-0.05682	0.439778	-0.1292	0.897198	0.988825
HSPA6	1.705371	1.218706	1.056848	1.153152	0.248848	0.988825
FCGR2B	0.557783	0.121078	1.456498	0.083129	0.933749	0.988825
KIFAP3	282.8838	0.382794	0.360077	1.063089	0.287742	0.988825
CTSE	0.186183	0.119168	2.011622	0.05924	0.952761	0.988825
ARF1	1202.92	0.105989	0.194335	0.54539	0.585485	0.988825
KIF3C	21.1961	0.25869	0.444856	0.581515	0.560893	0.988825
DCTN1	107.9738	0.138963	0.165412	0.8401	0.400852	0.988825
ACTR1B	145.2475	0.073089	0.222945	0.327835	0.743036	0.988825
DYNC1I2	289.5899	0.108015	0.188256	0.573766	0.566126	0.988825
PIKFYVE	126.8274	0.26713	0.277129	0.963917	0.335088	0.988825
AP1S3	4.167195	-0.31337	0.767013	-0.40857	0.682859	0.988825
SEC13	158.1496	-0.27511	0.384725	-0.71509	0.474551	0.988825
DYNC1LI1	269.9362	-0.04777	0.155421	-0.30734	0.758585	0.988825
KIF15	31.1745	-1.63753	0.530133	-3.08891	0.002009	0.152094
RAB7A	634.0579	-0.07361	0.455899	-0.16147	0.871724	0.988825
AP2M1	1195.822	0.068566	0.177453	0.386391	0.699207	0.988825
SEC31A	311.964	0.260143	0.233412	1.114521	0.265056	0.988825
CENPE	45.9271	-0.73496	0.467724	-1.57135	0.1161	0.988825
SEC24B	52.88656	-0.08531	0.303774	-0.28084	0.778833	0.988825
SEC24D	52.92592	0.153036	0.473963	0.322886	0.746782	0.988825
KIF2A	548.1408	-0.0482	0.474826	-0.1015	0.919153	0.988825
KIF3A	51.07994	0.320753	0.58931	0.544286	0.586245	0.988825
HSPA4	297.5453	-0.15858	0.206756	-0.76697	0.443098	0.988825
SAR1B	139.3448	-0.06129	0.276984	-0.22129	0.824864	0.988825

SEC24A	31.43042	0.133327	0.314292	0.424214	0.67141	0.988825
CD74	2790.643	-0.03405	0.437264	-0.07788	0.937927	0.988825
DCTN4	95.41146	0.250876	0.261436	0.959607	0.337253	0.988825
KIF4B	0.16624	-0.54948	3.07078	-0.17894	0.857987	0.988825
CANX	864.4631	-0.11539	0.164246	-0.70256	0.482327	0.988825
HLA-F	94.1821	0.362809	0.790636	0.458883	0.646319	0.988825
HLA-G	3.449744	0.549434	1.005337	0.546517	0.584711	0.988825
HLA-A	3508.894	0.1646	0.357624	0.46026	0.64533	0.988825
HLA-E	1861.988	0.307866	0.310191	0.992504	0.320952	0.988825
HLA-C	2411.047	0.173489	0.324496	0.53464	0.592899	0.988825
HLA-B	5424.809	0.104298	0.292429	0.356662	0.721345	0.988825
HSPA1L	2.496357	-0.65367	0.88389	-0.73954	0.459581	0.988825
HSPA1A	172.7175	0.00377	0.419049	0.008997	0.992822	0.992822
HSPA1B	40.81429	0.106966	0.609704	0.175439	0.860735	0.988825
HLA-DRA	3938.201	0.040011	0.238667	0.167643	0.866864	0.988825
HLA-DRB5	343.2015	-0.09411	1.304556	-0.07214	0.942488	0.988825
HLA-DRB1	1343.885	0.017805	0.497617	0.03578	0.971458	0.988825
HLA-DQA1	167.0825	0.100021	0.265354	0.376934	0.706223	0.988825
HLA-DQB1	197.683	0.017786	0.515584	0.034498	0.97248	0.988825
HLA-DQA2	79.3449	-0.02294	2.000848	-0.01146	0.990854	0.992822
HLA-DQB2	0.280173	0.710148	1.96718	0.360998	0.718101	0.988825
HLA-DOB	4.986122	-0.15633	1.13925	-0.13722	0.890858	0.988825
TAP2	26.85668	-0.15591	2.898351	-0.05379	0.957101	0.988825
TAP1	181.4744	0.215144	0.329121	0.653694	0.513309	0.988825
HLA-DMB	61.98056	0.398767	0.361713	1.102442	0.27027	0.988825
HLA-DMA	934.9557	0.049756	0.193715	0.256852	0.797293	0.988825
HLA-DOA	17.3017	0.10502	0.450828	0.232948	0.815801	0.988825
HLA-DPA1	1422.977	0.015697	0.278604	0.056342	0.955069	0.988825
HLA-DPB1	1627.107	0.034813	0.306079	0.113738	0.909446	0.988825
TAPBP	388.7285	0.074047	0.278034	0.266321	0.789992	0.988825
TREM2	0	NA	NA	NA	NA	NA
DYNC1I1	3.369577	-0.97212	0.835082	-1.1641	0.244384	0.988825

AP1S1	159.4662	-0.19482	0.591329	-0.32947	0.741804	0.988825
CAPZA2	1028.511	0.062133	0.230248	0.269855	0.787272	0.988825
AP1S2	336.6246	0.078263	0.2828	0.276743	0.781977	0.988825
KIF4A	15.51363	-1.47891	0.489437	-3.02166	0.002514	0.152094
DCTN6	350.9603	0.065673	0.438959	0.14961	0.881072	0.988825
SH3GL2	0.043764	0.811995	3.116575	0.260541	0.794447	0.988825
DCTN3	559.6287	0.216165	0.1613	1.340149	0.180197	0.988825
CLTA	1539.628	0.01222	0.212976	0.057375	0.954246	0.988825
CTSL	25.46232	0.437384	1.203897	0.363307	0.716376	0.988825
CTSV	0.60281	1.540511	1.422955	1.082614	0.27898	0.988825
HSPA5	845.621	-0.47483	0.283141	-1.67699	0.093544	0.988825
AP2A2	71.98104	0.166639	0.340346	0.489618	0.624404	0.988825
CTSD	286.9602	-0.06479	0.230716	-0.28082	0.77885	0.988825
KIF18A	30.4026	-0.78039	0.339911	-2.29587	0.021683	0.655916
TRAF6	36.18724	0.013622	0.350748	0.038838	0.96902	0.988825
KLC2	8.980641	0.468036	0.554121	0.844646	0.398309	0.988825
CTSF	130.1718	0.447858	0.244917	1.828612	0.067458	0.988825
SPTBN2	50.24012	0.337103	0.380715	0.885445	0.375917	0.988825
HSPA8	2599.266	-0.28977	0.472925	-0.61271	0.540067	0.988825
SEC24C	54.66376	-0.21768	0.221897	-0.98102	0.326585	0.988825
KIF11	49.98723	-0.82201	0.32038	-2.56572	0.010296	0.41528
ACTR1A	202.8012	0.059117	0.376042	0.157208	0.875081	0.988825
LAG3	3.621259	-0.05362	0.984064	-0.05449	0.956548	0.988825
CAPZA3	0	NA	NA	NA	NA	NA
RACGAP1	42.13749	-0.55817	0.496318	-1.12461	0.260753	0.988825
DCTN2	590.0174	0.047976	0.15867	0.302362	0.762376	0.988825
KIF5A	6.909263	0.230769	0.705911	0.32691	0.743736	0.988825
DYNLL1	1982.236	-0.0854	0.735248	-0.11616	0.907529	0.988825
PSME1	1457.847	0.010275	0.156977	0.065458	0.94781	0.988825
PSME2	888.1235	-0.19219	0.355071	-0.54128	0.588312	0.988825
SEC23A	90.1577	-0.07504	0.180976	-0.41466	0.678394	0.988825
ACTR10	345.2396	0.109052	0.173108	0.629969	0.528715	0.988825

HSPA2	3.74855	-0.62554	1.20528	-0.519	0.603762	0.988825
LGMN	2.827241	0.821682	1.104254	0.744106	0.456812	0.988825
DYNC1H1	326.1082	0.175394	0.285179	0.615032	0.538534	0.988825
KLC1	111.1819	0.297743	0.284488	1.046594	0.295287	0.988825
KIF26A	0.146462	0.412879	3.080674	0.134022	0.893385	0.988825
THBS1	15.95834	-0.33773	1.170769	-0.28847	0.772987	0.988825
PDIA3	1114.231	-0.21958	0.285643	-0.76872	0.442058	0.988825
B2M	21277.58	0.067721	0.211721	0.319861	0.749073	0.988825
KIF23	22.71569	-1.1597	0.593421	-1.95427	0.050669	0.988825
CIITA	23.21261	0.26475	0.41385	0.639724	0.522352	0.988825
DCTN5	77.54658	0.123123	0.315336	0.39045	0.696204	0.988825
KIF22	182.1778	-0.30375	0.317827	-0.9557	0.339222	0.988825
PYCARD	603.7711	0.094619	0.423986	0.223165	0.823407	0.988825
DYNC1LI2	177.7405	0.213045	0.331138	0.643372	0.519983	0.988825
AP1G1	100.5026	0.067477	0.36845	0.183138	0.85469	0.988825
RILP	193.65	0.287972	0.20279	1.420051	0.155593	0.988825
AP2B1	212.4885	0.021547	0.179535	0.120013	0.904473	0.988825
PSME3	121.2566	-0.0977	0.445863	-0.21913	0.826549	0.988825
KIF2B	0	NA	NA	NA	NA	NA
DYNLL2	155.4806	-0.05493	0.425355	-0.12913	0.897252	0.988825
CLTC	219.5876	0.034168	0.299838	0.113954	0.909274	0.988825
OSBPL1A	167.9804	-0.14951	0.420591	-0.35548	0.722229	0.988825
KIF3B	28.03689	0.239959	0.310451	0.772935	0.439561	0.988825
AP1M2	0.023253	1.243033	3.133281	0.396719	0.691574	0.988825
DNM2	186.3077	0.097744	0.323467	0.302177	0.762517	0.988825
CALR	2483.231	-0.57082	0.461026	-1.23816	0.215657	0.988825
AP1M1	97.20365	0.13774	0.261829	0.526068	0.598841	0.988825
IFI30	0.148653	1.357932	3.099762	0.438076	0.661331	0.988825
AP2S1	791.7988	-0.1196	0.184833	-0.64709	0.517576	0.988825
AP2A1	101.4671	0.149382	0.164237	0.909551	0.363059	0.988825
AP1B1	116.0323	-0.09774	0.177996	-0.54913	0.582916	0.988825
